# The impact of machine learning in predicting risk of violence: A systematic review

**DOI:** 10.3389/fpsyt.2022.1015914

**Published:** 2022-12-01

**Authors:** Giovanna Parmigiani, Benedetta Barchielli, Simona Casale, Toni Mancini, Stefano Ferracuti

**Affiliations:** ^1^Department of Human Neurosciences, Sapienza University of Rome, Rome, Italy; ^2^Department of Dynamic and Clinical Psychology, and Health Studies, Sapienza University of Rome, Rome, Italy; ^3^Department of Computer Science, Sapienza University of Rome, Rome, Italy

**Keywords:** artificial intelligence, machine learning, forensic setting, clinical setting, violence assessment

## Abstract

**Background:**

Inpatient violence in clinical and forensic settings is still an ongoing challenge to organizations and practitioners. Existing risk assessment instruments show only moderate benefits in clinical practice, are time consuming, and seem to scarcely generalize across different populations. In the last years, machine learning (ML) models have been applied in the study of risk factors for aggressive episodes. The objective of this systematic review is to investigate the potential of ML for identifying risk of violence in clinical and forensic populations.

**Methods:**

Following Preferred Reporting Items for Systematic Review and Meta-Analyses (PRISMA) guidelines, a systematic review on the use of ML techniques in predicting risk of violence of psychiatric patients in clinical and forensic settings was performed. A systematic search was conducted on Medline/Pubmed, CINAHL, PsycINFO, Web of Science, and Scopus. Risk of bias and applicability assessment was performed using Prediction model Risk Of Bias ASsessment Tool (PROBAST).

**Results:**

We identified 182 potentially eligible studies from 2,259 records, and 8 papers were included in this systematic review. A wide variability in the experimental settings and characteristics of the enrolled samples emerged across studies, which probably represented the major cause for the absence of shared common predictors of violence found by the models learned. Nonetheless, a general trend toward a better performance of ML methods compared to structured violence risk assessment instruments in predicting risk of violent episodes emerged, with three out of eight studies with an AUC above 0.80. However, because of the varied experimental protocols, and heterogeneity in study populations, caution is needed when trying to quantitatively compare (e.g., in terms of AUC) and derive general conclusions from these approaches. Another limitation is represented by the overall quality of the included studies that suffer from objective limitations, difficult to overcome, such as the common use of retrospective data.

**Conclusion:**

Despite these limitations, ML models represent a promising approach in shedding light on predictive factors of violent episodes in clinical and forensic settings. Further research and more investments are required, preferably in large and prospective groups, to boost the application of ML models in clinical practice.

**Systematic review registration:**

[www.crd.york.ac.uk/prospero/], identifier [CRD42022310410].

## Introduction

Violent behavior in clinical psychiatric and forensic settings is a major issue for health sectors, with effects on the well-being of both patients and psychiatric staff (1), together with economic consequences associated with trauma, staff illness, and potential lawsuit by victims (2). Iozzino et al., in a meta-analysis investigating data from 35 sites around the world, found that 14–20% of patients tend to engage at least once into violent behavior during inpatient treatment (3). Being a victim of physical aggression has been reported by 70% of staff in forensic psychiatry settings (4).

Several risk factors of violent behavior have been identified and have been grouped into “static” and “dynamic” (5). The term “static risk factors” refers to characteristics that are stable over time, such as age, gender, family history, traumatic experiences, or offenses during childhood. They are useful during risk assessment to predict violence in the long term. On the contrary, the term “dynamic risk factors” refers to those aspects that can change and may represent a target for intervention (for example, psychiatric symptoms, misuse of alcohol or other substances, and non-adherence to treatment). Dynamic risk factors tend to predict violent behavior in the short-term. Consequently, both static and dynamic factors should be evaluated during risk assessment and be employed to develop strategies to prevent or minimize the impact of violent behavior (5).

In managing patient violence, an important aspect is to correctly assess the presence of prospective risk of violent behavior. The reliability of clinical judgment alone has been widely questioned for several limitations, such as poor inter-rater reliability among evaluators, confirmation bias, and the tendency to human error (6). To overcome this issues, structured violence risk assessment tools have been developed, among which the most commonly used are the Violence Risk Appraisal Guide (7), Structured Assessment of Violence Risk in Youth (8), and Historical Clinical Risk Management-20 (9). They are based both on static and dynamic risk factors and show a predictive validity surpassing that of unstructured clinical judgments, with a good median performance (between 0.70 and 0.74) in predicting violent behavior (10). Nevertheless, the use of these instruments in clinical practice resent from several limitations, such as the long time needed to perform a structured assessment (which may require hours) and the finding that just a small subset of risk factors can generalize to different populations.

In the last years, a growing interest emerged in the use of artificial intelligence (AI), mainly machine learning (ML) techniques, to improve accuracy, objectivity, transparency, and reliability in clinical decision making. In the mental health area, ML has been applied to predict therapeutic outcomes in depression (11) and suicide in civil (12) and military subjects (13). A technique which is referred to as multi-voxel pattern analysis (MVPA) (14) has been used to identify patterns of brain activity or structures that reliably predict disease onset (15) or distinguish treatment responders from non-responders (16). Finally, ML has been employed to investigate the risk factors for aggressive episodes both in clinical (17) and forensic settings (18).

To the best of our knowledge, no systematic review evaluated the performance of ML models for predicting aggression in clinical psychiatric and forensic patients. Therefore, we conducted a systematic review to investigate the potential of ML for identifying risk of violence and to explore the performance measures of these models for predicting aggression and/or violent behavior in clinical and forensic populations. The correct identification and prediction of aggressive episodes has, in fact, important implications for the prevention of violent incidents and the treatment of violent patients.

### Machine learning

Machine learning is a branch of AI aimed at making a computer able to automatically learn a general model from available data (19). In particular, *supervised* ML is typically used to learn a general correspondence from observations (i.e., values for a set of input variables, or features) to an outcome (e.g., a value for a given output variable).

The learning task is performed starting from a dataset (called *training set*), whose entries define values for the input features as well as for the outcome variable (aka *ground truth*). Supervised ML algorithms try to learn a *model* (a mathematical function) that predicts the value for the outcome variable for *any possible* assignment of values to the input features. By trying to keep the learned model as simple as possible (according to the famous Ockham’s razor principle), ML algorithms attempt to capture hidden patterns in the input dataset and to *generalize* from it.

A plethora of different algorithms and model types have been proposed in the ML literature, among which: decision trees, random forests, naïve Bayes, gradient boosting machines, support vector machines, neural networks and ensembles thereof, as well as many variations and combinations of techniques.

To evaluate the quality of a learned model, a validation procedure is typically included, which compute suitable performance measures on an independent dataset. When the outcome variable can assume only two values, e.g., violent/non-violent, typical performance measures are: *accuracy* (the ratio of correct predictions), which can be broken down into *sensitivity* and *specificity* (the ratios of correctly predicted true positives and true negatives, respectively). The latter measures are often synthesized into a single value, the Area Under (the Receiver Operator Characteristic) Curve (AUC-ROC, or simply AUC) which ranges from 0 to 1. Higher AUC values denote overall better discrimination ability. Especially when the available data is limited, one of the most common validation techniques used is the *k*-fold cross-validation (*k*-CV), which consists in randomly splitting the available dataset into *k* ≥ 2 slices. Each slice is considered in turn as the validation set for a model learned from the other *k−*1 slices (training set), and average performance of the *k* learned models is computed. Typical values for *k* are 5 or 10. When *k* is 2, the algorithm reduces to the basic 50–50% cross-validation (2-CV), where a single model is learned from (randomly selected) half dataset and evaluated against the other half. On the other extreme, when *k* equals the number of data entries, the algorithm is called leave-one-out cross-validation (LOOCV).

## Materials and methods

This systematic review was conducted in accordance with the Preferred Reporting Items for Systematic Review and Meta-Analyses (PRISMA) guidelines (20).

### Literature search

We used a systematic search strategy to identify articles relevant to our review. A two-step literature search was conducted on 12 February 2022. Firstly, the Medline/Pubmed, CINAHL, PsycINFO, Web of Science, and Scopus databases were searched, with the following string: (“artificial intelligence” or “machine learning” or “deep learning”) AND (“aggression” or “violence” or “assault”).

As a second step, two investigators (SC and BB) implemented the search through a manual inspection of the reference lists of the retrieved papers. Abstracts of articles identified through these two steps were then screened for eligibility, and the remaining articles were assessed for eligibility based on a full-text reading. When discrepancies emerged, a third author (GP) was consulted, and eventually, Delphi rounds with all other authors were performed. The protocol for this review has been registered in the international prospective register of systematic reviews (PROSPERO registration number CRD42022310410).

### Inclusion and exclusion criteria

Articles were included if dealing with the use of ML techniques in predicting risk of violence. Articles written in languages other than English, Italian, or Spanish, reviews, and those whose full text was unavailable even after contacting the corresponding author were excluded.

### Data extraction (selection and coding)

Two reviewers independently, in duplicate, screened titles and abstracts to determine whether the retrieved studies met the above-outlined inclusion criteria.

For studies apparently meeting inclusion criteria or where a decision could not be made from the title and/or abstract alone, full texts were obtained, for a detailed review against inclusion criteria. Two reviewers independently assessed the eligibility of these full-texts in our study. When discrepancies emerged, these were resolved by an initial discussion with a third reviewer, and possibly, with Delphi rounds, until complete consensus was reached.

To extract data from the included articles, a standardized form was used, to assist in study quality and evidence synthesis. Data points extracted from the studies have been guided by the CHecklist for critical Appraisal and data extraction for systematic Reviews of prediction Modeling Studies (CHARMS) checklist (21) and Transparent Reporting of a multivariable prediction model for Individual Prognosis Or Diagnosis (TRIPOD) guidelines (22). Extracted information included: the focus of the study, sample characteristics, ML approach type and validation method used, performance measures of the model, features examined, type of setting (forensic or clinical), and authors conclusions, as well as information required for assessment of the Risk of Bias (RoB). Two reviewers independently, in duplicate, completed data extraction, and a third reviewer was consulted when needed.

### Quality evaluation

Risk of Bias and applicability assessment was performed using Prediction model Risk Of Bias ASsessment Tool (PROBAST) (23), by two reviewers independently, in duplicate, with a third reviewer to manage any disagreements. PROBAST is a tool designed to assess studies that develop, validate, or update (for example, extend) multivariable prediction models for diagnosis or prognosis. It includes 20 signaling questions across 4 domains: (a) *Participants*: deals with potential biases associated with the selection of participants and data sources used; (b) *Predictors*: assesses potential sources of bias from the definition and measurement of the candidate predictors; (c) *Outcome*: evaluates the methods and timing for the definition of the outcome; and (d) *Analysis*: analyses the statistical methods employed to develop and validate the model, such as study size, handling of continuous predictors and missing data, selection of predictors, and model performance measures.

An overall assessment of RoB is determined by a ranking system of *low*, *high*, or *unclear*. Disagreements were resolved through Delphi rounds until full consensus was reached.

## Results

**FIGURE 1:**
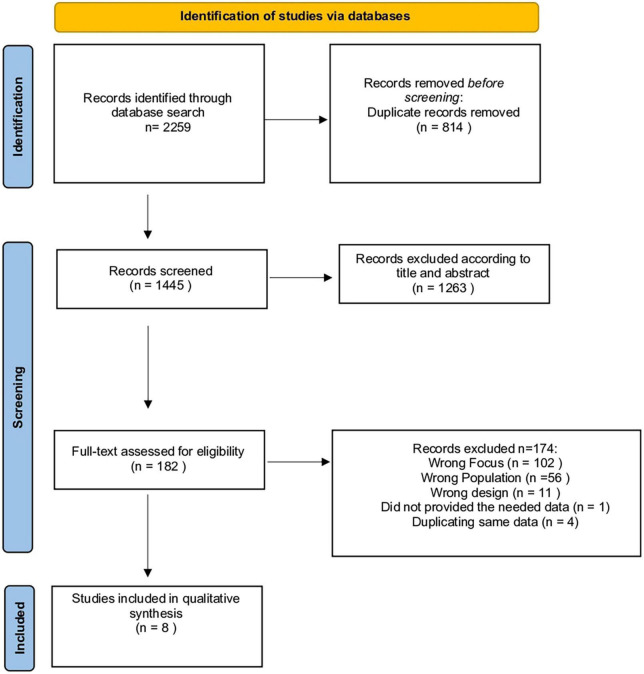
Prisma flow diagram.

We identified 182 potentially eligible studies from 2,259 records obtained from the selected databases. After reviewing the full content of the articles, 174 of them were excluded for several reasons: 102 did not investigate the use of ML models in predicting risk of aggression/violent behavior, 56 examined a different population, 11 were editorials or reviews, 1 did not provide the needed data even after their authors were contacted, and 4 contained duplicate data. The process of identifying eligible studies is outlined in Figure 1. For the list of the excluded studies see the Supplementary Data file.

### Study characteristics

The characteristics of the included studies and ML models are summarized in Tables 1, 2. One study was conducted in the United States (17), two in China (18, 24), two in Canada (25, 26), two in Netherlands (27, 28), and one in Switzerland (29). We classified the studies in terms of the size of their samples into: *small* [<100 data entries, one study (18)], *medium* [200–900, three studies (24, 25, 29)], and *large* [≥1,000, four studies (17, 26–28)].

**TABLE 1:** Studies using ML models for predicting risk of aggression and/or violence.

References	Focus of the study	Sample	ML approach type	Main finding
**Forensic setting**				
Gou et al. (18)	Identification of violent patients with schizophrenia	75 psychiatric patients with schizophrenia	LASSO + support vector machine (voting)	Alterations in the prefrontal-temporal cognitive circuit and striatum reward system, hostility, psychopathy, and the overall score on the HCR-20 scale, had a fair predictive value for identifying violent patients *via* a cumulative effect.
Kirchebner et al. (29)	Analyze the impact of accumulation and type of stressor on committing an offense in patients with schizophrenia spectrum disorders	370 forensic patients with schizophrenia spectrum disorders	Support vector machine, logistic regression, *k*-nearest neighbors, trees	Coercive psychiatric treatment, unemployment, and separation from caregivers in childhood were related to violent offending.
Watts et al. (26)	To develop a machine learning model to predict the type of criminal offense committed by forensic patients	1,240 forensic patients	Random forest, elastic net, support vector machine	Impairments in impulse control, lack of current sources of income, substance abuse, and the presence of aggression distinguished between psychiatric patients who have committed sexual, non-violent, and violent criminal offenses.
**Clinical setting**				
Lu et al. (24)	To identify psychosocial factors predictive of aggression in psychiatric patients with drug addiction	896 psychiatric patients with drug addiction	Gradient boosted regression trees	Interpersonal trust, psychological security, psychological capital, parental conflict and alexithymia are predictive of aggression.
Menger et al. (27)	Predicting violence incidents during psychiatric admission	2,521 psychiatric admissions from 1,796 unique patients	(Recurrent, convolutional) neural network, naive Bayes, support vector machine, decision tree	The best result is obtained by combining document embeddings with a recurrent neural network.
Menger et al. (28)	Identifying inpatients who show violent behavior during the first 4 weeks of admission	4,128 psychiatric patients	Support vector machine	Several terms, such as aggressive, angry, verbal, threatening, and irritated, can directly be associated with violence.
Suchting et al. (17)	Predicting patient aggressive events in a psychiatric hospital	29,841 psychiatric patients	Penalized generalized linear modeling, random forest, gradient boosting machine, deep neural networks	The strongest predictors of aggressive events included homelessness, having been convicted of assault, and having witnessed abuse.
Wang et al. (25)	To develop a predictive model to identify patients affected by schizophrenia with violent tendencies	275 patients affected by schizophrenia	LASSO, elastic net, random forest, gradient boosted regression trees, support vector machine, support vector machine with radial basis function kernels	Random forest model performed marginally better than other algorithms.

LASSO: least absolute shrinkage and selection operator.

**TABLE 2:** Features and predictors of the ML models.

References	Features	Outcome	Best performing algorithm	Validation	AUC	Accuracy (%)	Sensitivity (%)	Specificity (%)	Predictors
Gou et al. (18)	Education BPRS-4 activation BPRS-5 hostility PCL-SV total score HCR-20 total score BIS-11 total score Gray matter volume Regional homogeneity Fractional anisotropy	**Violent** = the Modified Overt Aggression Scale (MOAS) a score of ≥3 for item 4 (physical aggression scale) of the MOAS; **Non-violent** = a score of <2 for item 4 of the MOAS and were free of any severe aggressive act against property and/or themselves.	LASSO regression + support vector machine	LOOCV	0.95	90.67	90.91	90.48	Education BPRS-5: hostility PCL-SV total score HCR-20 total score Gray matter volume Regional homogeneity Fractional anisotropy
Kirchebner et al. (29)	**Stressors in childhood/youth** Bullying Separation/divorce of caregivers Impairment of the parent–child relationship Physical abuse by the caregiver Sexual abuse by the caregiver Poverty Separation from caregiver Rejection/being ignored by the caregiver active devaluation by the caregiver Poor parenting methods Violent physical illness of the patient Failure in school **Stressors in adulthood** Unemployment (at time of offense) homelessness Conflicts in the workplace Social isolation Violent victimization **Psychiatric stressors** Coercive psychiatric treatment At least three previous hospitalizations Compulsory psychiatric placement Positive symptoms during criminal offense	*Violent offense = homicide and attempted homicide, assault, rape, robbery, arson, and child abuse; **Non-violent offense** = threat, theft, damage to property, minor sexual offenses (e.g., exhibitionism), drug offenses, illegal gun possession, and other minor offenses (e.g., triggering false alarms or emergency brakes)	Boosted classification trees	5-CV	0.83	77	80.49	71.19	Social isolation in adulthood Coercive psychiatric treatment Unemployment (at time of offense) Separation from the family/caregivers in the patient’s childhood/youth Failure in school
Lu et al. (24)**	Drug Craving Scale (DCS) Buss-Warren Aggression Questionnaire Revised in China (BWAQ-RC) Impulsivity Scale Security Questionnaire (SQ) Positive Psychological Capital Questionnaire (PPCQ) Toronto Alexithymia Scale (TAS-20) Children’s Perception of Inter-parental Conflict Scale (CPIC) Interpersonal Trust Scales (ITS) Year of birth	Not clearly defined	Gradient boosted regression trees	5-CV and out-of-sample testing techniques	–	–	–	–	Interpersonal trust (ITS) Psychological security (PPCQ) Psychological capital (PPCQ) Parental conflict (CPIC) Alexithymia (TAS-20)
Menger et al. (27)	**Doctor textual notes** with information, such as patient history, current treatment (e.g., types of medication and therapy), and changes therein **Nurse textual notes** with information on the current wellbeing and activities of a patient	**Violent incidents** = incidents concerned violence from patients directed at staff or at other patients, including both verbal and physical aggression in the first 30 days after admission	Recurrent neural network	5-CV	0.79	–	–	–	–
Menger et al. (28)***	The 1,000 most frequent terms in the clinical notes	**Violent incident** = all threatening and violent behavior of a verbal or physical nature directed at another person	Support vector machine	5-CV	0.76	–	33.4–33.6	93.5–94.7	**Site 1:** the terms aggressive, reacts, and offered generalize **Site 2:** the terms verbal, threatening, and aggression
Suchting et al. (17)	**328 predictor variables** among which full demographic profile, patient vitals (i.e., height, weight, and blood pressure), a comprehensive psychosocial assessment, including histories of early development, education, military service, vocation/work, medical status, psychiatric status, drug/substance use and treatment, nicotine/tobacco use and counseling, abuse (victim or perpetrated physical/verbal/emotional/sexual abuse), legal status, marital status, religious beliefs, financial status, and living situation. Sleep habits, pain status, patient behavior during interview, a risk assessment, and evaluation of patient mood (*via* the Affective Disorders Rating Scale. General appearance (i.e., hygiene), musculoskeletal system, speech pattern, thought processes and content, perception, depression, affect, insight, judgment, skin integrity, head trauma, suicidal/homicidal/assault ideation, deterioration in function, chemical dependency, hallucinations, and delusions.	**Aggressive event** = it is coded into the hospital medical record following any episode of uncontrolled verbal or physical aggression that required intervention by and assistance from additional hospital staff to manage the event	Penalized generalized linear modeling	5-CV	0.78	–	–	–	Current living situation (homeless) Legal history – assault conviction Abuse history – witness (other) Abuse history – perpetrated (other) Age
Wang et al. (25)	**28 predictive variables** among which age, sex, age of onset of psychosis, number of previous psychiatric hospitalizations, comorbid diagnoses of lifetime alcohol, drug, and marijuana abuse or dependence, family histories of psychosis, mood disorders, suicide, ethnicity, primary language, religious identity, age of immigration, childhood trauma and five-factor personality traits from the NEO Five Factor Inventory (NEO-FFI).	Not clearly defined	Random forest	5-CV	0.63	62	32	80	–
Watts et al. (26)*	**138 variables** among which adverse events in childhood, income, housing, comorbidities, family history, prescribed medications, substance use, and presumed indicators of risk. Variables were transformed *via* one-hot encoding into new binary variables. This resulted in 156 candidate features.	Patients were divided into **violent**, **non-violent** and **sexual offenses** according to the most recent criminal offense for which they were found not criminally responsible. In cases where multiple crimes were committed, patients were divided according to the most serious offense committed	Elastic net	10-CV	0.88	80.34	83.26	77.42	*Sexual vs. violent offenses*: paraphilia; previous sexual conviction; dementia/cognitive disorder; living off family support; female support
					0.78	68.79	69.84	67.74	*Sexual vs. non-violent offenses*: paraphilia; schizoaffective disorder; female gender; history of sexual aggression against others; impulse control disorder
					–	–	–	–	*Sexual vs. all offenses*: previous absolute discharge; previous sexual convictions; female gender; anti-androgen medication; cluster A personality disorder

BPRS, Brief Psychiatric Rating Scale; PCL-SV, Psychopathy Checklist-Screening Version; HCR-20, Historical, Clinical and Risk Management-20; BIS-11, Barratt Impulsiveness Scale-11.

*Based on Swiss law.

**Five most important variables by a variable importance plot.

***The top three terms with highest within–data set generalizability (ratio).

Three studies focused on the predictive model of aggression specifically in patients affected by schizophrenia spectrum disorders: two of them in a forensic setting (18, 29), and one in a clinical setting (25). One study enrolled patients affected by drug addiction (24). Three studies used information of psychiatric patients from Electronic Health Records (EHR) (17, 27, 28), and one analyzed retrospective information of patients from 10 forensic psychiatry facilities (26). Only one study (28) used an independent cohort to validate the ML model, while the remaining ones used only internal cross-validation (17, 18, 24–27, 29). Three studies out of eight reported ML algorithms with AUC above 0.80 (18, 26, 29), which is an indication of good discrimination ability. No studies reported data about pre-processing procedures, namely data preparation and curation.

### Quality evaluation

Table 3 summarizes the different aspects concerning the methodological quality of the studies included in our review.

**TABLE 3:** Quality assessment through Prediction model Risk Of Bias ASsessment Tool (PROBAST).

References	Risk of bias (RoB)				Applicability			Overall	
			
	Participants	Predictors	Outcome	Analysis	Participants	Predictors	Outcome	RoB	Applicability
Gou et al. (18)	+	−	+	−	−	+	+	−	−
Kirchebner et al. (29)	−	+	+	+	−	+	+	−	−
Lu et al. (24)	−	?	?	−	−	+	−	−	−
Menger et al. (27)	−	+	+	+	+	+	+	−	+
Menger et al. (28)	−	+	+	+	+	+	+	−	+
Suchting et al. (17)	−	+	+	+	+	+	+	−	+
Wang et al. (25)	+	+	−	+	−	+	+	−	−
Watts et al. (26)	−	+	+	+	+	+	+	−	+

“+” indicates low RoB/low concern regarding applicability; “−” indicates high RoB/high concern regarding applicability; “?” indicates unclear RoB/unclear concern regarding applicability.

Regarding RoB, seven out of eight studies scored high in RoB on participant selection for the following reasons: five used data from existing sources, such as EHR (17, 25–28), or medical records (29) collected for different purposes and without a protocol; one did not perform a consecutive recruitment of patients (24). One study scored high in RoB on predictors, because assessment of predictors was not made without knowledge of outcome data (18), and one scored unclear in the predictors domain because it was not clear if assessment of predictors was made without knowledge of outcome data (24). One study scored unclear in the outcome session because authors did not clearly define when and how they measured the outcome (24). One study scored high in RoB in the outcome session, because authors did not clearly define how they measured the outcome (25). Two studies scored high in RoB in the analysis session: one because there were too many predictors and a small number of patients with the outcome event (18); one because they did not provide information on number of participants with the outcome, nor on performance measure of the model (24).

Regarding applicability, four out of eight studies scored high in RoB on participant selection because they evaluated only patients affected by schizophrenia spectrum disorders (18, 25, 29) or drug addiction (24), and consequently the sample was not fully representative of the population specified in our review question, composed by psychiatric patients in clinical and forensic settings. One study scored high in applicability in the outcome domain because it was not clear at what point and how the outcome was determined (24).

## Discussion

This systematic review on ML techniques for predicting risk of violent episodes in psychiatric patients showed a general trend toward competitive performance. All the studies, in fact, reported quite high AUC values, with values ranging from 0.63 to 0.95 [three studies report AUC above 0.80 (18, 26, 29)]. Overall, it seems that ML-based approaches have the potential to (or already might) outperform the predictive validity of current violence risk assessment tools, whose benefit in clinical practice seems to be moderate (30). However, although, to our knowledge, this is the first review to analyze the performance of ML models for prediction of violence, we must be very careful when trying to quantitatively compare (e.g., in terms of AUC) and derive general conclusions from the approaches presented here (or a ranking thereof). In fact, the eight considered studies are based on different experimental protocols and focus on different clinical and forensic populations, such as patients affected by schizophrenia spectrum disorders, drug addiction, and general psychiatric disorders. This wide variability in the experimental settings and characteristics of the enrolled samples is likely to be a major cause for the absence of shared common predictors of violence found by the models learned.

Predictors of violent episodes in forensic inpatients affected by schizophrenia spectrum disorders can in fact be quite different from those present in patients affected by personality disorder hospitalized in a psychiatric ward.

Regarding the forensic setting, for example, Gou et al. (18), found education, hostility, PCL-SV total score, HCR-20 total score, and dysfunction in cortical-subcortical circuits to be associated with a higher risk of violence in patients with schizophrenia. Kirchebner et al. (29), in a forensic sample of 370 patients affected by schizophrenia, emphasized the role of social isolation in adulthood, coercive psychiatric treatment, unemployment at time of offense, separation from the family/caregivers in the patient’s childhood/youth and failure in school, as life stressors involved in the development of violent offending. Watts et al. (26) in a sample of 1,240 forensic patients found that impairments in impulse control, lack of current sources of income, substance abuse, and the presence of aggression distinguished between psychiatric patients who have committed sexual, non-violent, and violent criminal offenses.

In the clinical setting, in patients affected by drug addiction, a high level of interpersonal trust, psychological security and psychological capital were protective factors, while a high level of parental conflict and alexithymia were predictive of a high level of aggression (24). Menger et al. (28), by analyzing 4,128 EHR of clinical psychiatric patients, found that specific terms found in textual clinical notes (such as *aggressive*, *reacts*, and *threatening*) were predictive of violent episodes occurrence. Finally, Suchting et al. (17) evaluated 29,841 EHR of clinical psychiatric patients and found that being homeless, having been convicted of assault, and having witnessed abuse were the strongest predictors of patient aggressive events.

Such variability is not surprising, given the high heterogeneity of the employed populations, the different features used to learn models [some of them being automatically extracted from textual notes *via* natural language processing techniques, e.g., Menger et al. (28)], and the different definitions of the outcome variable (violent episodes). In addition, a wide variation was noted regarding sensitivity (the ratio of correctly predicted positives) which ranged from 32 to 90.91%, while specificity (or the ratio of correctly predicted negatives) ranged from 67.7 to 94.7%. We deem that such variability may stem from the heterogeneity and representativeness of data. Another characteristic that deserves attention when developing a prediction model is sample size which depends also on the representativeness of data, and best practices for its definition have been proposed (31). All the studies included in our review showed a wide heterogeneity among sample size and were classified into three groups: *small* sample size [<100 entries, one study (18)], *medium* sample size [200–900 entries, three studies (24, 25, 29)], and *large* sample size [≥1,000 entries, four studies (17, 26–28)]. All this hinders the possibility to accurately compare the various approaches and makes the current findings preliminary rather than conclusive. Indeed, it was not possible to conduct quantitative analyses and comparisons of the findings across different studies. The overall quality of the included studies, in our opinion, suffers from objective limitations, difficult to overcome, such as the common use of retrospective data. Although, on one hand, this allows researchers to recruit many participants, on the other leads to a high RoB, because the learning process is driven by information collected for other purposes. Only one study (28) performed an external validation, thus checking for a generalizability of the model to data from a different site. No study, however, incorporated the learned model into a decision support software system to be used as a guide in clinical and forensic practice. Finally, ML itself has known limitations: in particular, since data used as training set may be incomplete, noisy, or subject to systematic bias, the learned models might yield erroneous or biased predictions. Overall, this constitutes a limitation to the development of a decision support software systems that could be useful to predict violent episodes in patients affected by psychiatric disorders, independently from the forensic or general psychiatric setting. For this reason, the majority of studies using ML are focusing on more personalized diagnostic and treatment approaches, with a general trend toward different prediction tools designed for various settings and subgroups of patients.

With these caveats in mind, we believe that the findings from this systematic review demonstrate that ML is a promising approach and can become a valuable addition is studying predictive factors of violent episodes in clinical and forensic settings. The advantages of ML are numerous: these techniques can offer objective, data-based assessments that by standardizing the decisional process can avoid evaluation errors linked to the subjectivity and questionable reliability of clinical and forensic assessments. We deem that ML methods, employed in combination with the clinical interview and traditional psychometric tools, will represent in the future a valuable and reliable aid in clinical and forensic decision making. Patients’ aggression, in fact, is still an ongoing challenge to organizations and practitioners. Aggressive episodes can lead to physical and psychological trauma to other patients, staff, and visitors. More investments and research are required, preferably in large and prospective groups, to boost the application of ML in clinical practice. This will not only increase our comprehension of the characteristics of people at risk of becoming aggressive, but could also be informative for organizations and practitioners to developing training and support strategies for the management of violence in clinical and forensic settings.

## Data availability statement

The original contributions presented in this study are included in the article/Supplementary material, further inquiries can be directed to the corresponding author.

## Author contributions

GP, TM, and SF: conceptualization. GP, SC, and BB: design and methodology and conduction of the study. GP and BB: analysis and interpretation. GP: writing—original draft preparation. BB, SC, and TM: writing—review and editing. SF: supervision. All authors contributed to the article and approved the submitted version.
