# Supplementary material for: The impact of machine learning in predicting risk of violence: A systematic review
Source: Front Psychiatry. 2022 Dec 1;13:1015914. doi: 10.3389/fpsyt.2022.1015914 (PMC9751313; doi:10.3389/fpsyt.2022.1015914)
Supplement: Supplementary file 1 [file Data_Sheet_1.docx]

Supplementary Material

# Search strategies and retrieved records from each electronic database

**1. PubMed**

Date of search: 12/02/2022

Search strategy:

("artificial intelligence" or "machine learning" or "deep learning") AND ("aggression" or "violence" or "assault")

n findings: 204

**2. Web of Science**

Date of search: 12/02/2022

Search strategy:

("artificial intelligence" or "machine learning" or "deep learning") AND ("aggression" or "violence" or "assault")

n findings: 758

**3. CINAHL**

Date of search: 12/02/2022

Search strategy:

("artificial intelligence" or "machine learning" or "deep learning") AND ("aggression" or "violence" or "assault")

n findings: 55

**4. PsycINFO**

Date of search: 12/02/2022

Search strategy:

("artificial intelligence" or "machine learning" or "deep learning") AND ("aggression" or "violence" or "assault")

n findings: 159

**5. Scopus**

Date of search: 12/02/2022

Search strategy:

( TITLE-ABS-KEY ( "artificial intelligence" OR "machine learning" OR "deep learning" ) ) AND ( TITLE-ABS-KEY ( "aggression" OR "violence" OR "assault" ) )

n findings: 1083

# Excluded studies

| **Study** | **Reason for exclusion** |
| --- | --- |
| 1. Abdullah, S., Nibir, F. I., Salam, S., Dey, A., Alam, M. A., & Reza, M. T. (2020, December). Intelligent Crime Investigation Assistance Using Machine Learning Classifiers on Crime and Victim Information. In 2020 23rd International Conference on Computer and Information Technology (ICCIT) (pp. 1-4). IEEE. | Wrong Population |
| 1. Adesola, F., Azeta, A., Oni, A., Chidozie, F., & Azeta, V. Predicting Violent Crime Occurrence: An Evaluation of Decision Tree Model. | Wrong Population |
| 1. Agarwal, P., Sharma, M., & Chandra, S. (2019, August). Comparison of machine learning approaches in the prediction of terrorist attacks. In 2019 Twelfth International Conference on Contemporary Computing (IC3) (pp. 1-7). IEEE. | Wrong Population |
| 1. Al-Garadi, M. A., Hussain, M. R., Khan, N., Murtaza, G., Nweke, H. F., Ali, I., ... & Gani, A. (2019). Predicting cyberbullying on social media in the big data era using machine learning algorithms: review of literature and open challenges. IEEE Access, 7, 70701-70718. | Wrong Focus |
| 1. An Empirical Analysis of Machine Learning Algorithms for Crime Prediction Using Stacked Generalization: An Ensemble Approach | Wrong Focus |
| 1. Anastasopoulos, L. J., & Williams, J. R. (2019). A scalable machine learning approach for measuring violent and peaceful forms of political protest participation with social media data. Plos one, 14(3), e0212834. | Wrong Focus |
| 1. Anderson, N. E., Harenski, K. A., Harenski, C. L., Koenigs, M. R., Decety, J., Calhoun, V. D., & Kiehl, K. A. (2019). Machine learning of brain gray matter differentiates sex in a large forensic sample. Human brain mapping, 40(5), 1496-1506. | Wrong Focus |
| 1. Aydogan, A. F., & Shashidhar, N. (2021, June). Prevention Pre-Violence in E-Labs with Machine Learning: PVE. In 2021 9th International Symposium on Digital Forensics and Security (ISDFS) (pp. 1-5). IEEE. | Wrong Focus |
| 1. Baćak, V., & Kennedy, E. H. (2019). Principled machine learning using the super learner: an application to predicting prison violence. Sociological Methods & Research, 48(3), 698-721. | Wrong Focus |
| 1. Bagozzi, B. E., & Koren, O. (2017, December). Using machine learning methods to identify atrocity perpetrators. In 2017 IEEE International Conference on Big Data (Big Data) (pp. 3042-3051). IEEE. | Wrong Focus |
| 1. Ballesteros, M. F., Sumner, S. A., Law, R., Wolkin, A., & Jones, C. (2020). Advancing injury and violence prevention through data science. Journal of safety research, 73, 189-193. | Wrong Population |
| 1. Bappee, F. K. (2017, May). Identification and classification of alcohol-related violence in nova scotia using machine learning paradigms. In Canadian Conference on Artificial Intelligence (pp. 421-425). Springer, Cham. | Wrong Population |
| 1. Barzman, D., & Ni, Y. (2021). 2.1 Using Artificial Intelligence to Identify Risk of School Violence. Journal of the American Academy of Child & Adolescent Psychiatry, 60(10), S144-S145. | Wrong Population |
| 1. Barzman, D., Ni, Y., Griffey, M., Bachtel, A., Lin, K., Jackson, H., ... & DelBello, M. (2018). Automated risk assessment for school violence: a pilot study. Psychiatric quarterly, 89(4), 817-828. | Wrong Population |
| 1. Berk, R. A. (2021). Predictive Policing, and Risk Assessment for Law Enforcement. Annu. Rev. Criminol, 4(209), 37. | Wrong Focus |
| 1. Berk, R. A., & Sorenson, S. B. (2020). Algorithmic approach to forecasting rare violent events: An illustration based in intimate partner violence perpetration. Criminology & Public Policy, 19(1), 213-233. | Wrong Population |
| 1. Berk, R. A., Sorenson, S. B., & Barnes, G. (2016). Forecasting domestic violence: A machine learning approach to help inform arraignment decisions. Journal of empirical legal studies, 13(1), 94-115. | Wrong Population |
| 1. Berk, R., & Elzarka, A. A. (2020). Almost politically acceptable criminal justice risk assessment. Criminology & Public Policy, 19(4), 1231-1257. | Wrong Focus |
| 1. Berly, A., Manaouil, C., & Dervaux, A. (2020). L’intelligence artificielle peut-elle aider à estimer le risque de récidive dans les comportements violents?. Médecine & Droit, 2020(163), 105-109. | Wrong Population |
| 1. Bernecker, S. L., Rosellini, A. J., Nock, M. K., Chiu, W. T., Gutierrez, P. M., Hwang, I., ... & Kessler, R. C. (2018). Improving risk prediction accuracy for new soldiers in the US Army by adding self-report survey data to administrative data. BMC psychiatry, 18(1), 1-12. | Wrong Population |
| 1. Bex, F., & Hovestad, B. (2016, August). An argumentative-narrative risk assessment model. In 2016 European Intelligence and Security Informatics Conference (EISIC) (pp. 176-179). IEEE. | Wrong Focus |
| 1. Bhalla, S., & Kumar Singh, R. (2021). Exploration of Crime Detection Using Deep Learning. In Innovations in Cyber Physical Systems (pp. 297-304). Springer, Singapore. | Wrong Design |
| 1. Bilewicz, M., Tempska, P., Leliwa, G., Dowgiałło, M., Tańska, M., Urbaniak, R., & Wroczyński, M. (2021). Artificial intelligence against hate: Intervention reducing verbal aggression in the social network environment. Aggressive behavior, 47(3), 260-266. | Wrong Focus |
| 1. Blair, R. A., Blattman, C., & Hartman, A. (2017). Predicting local violence: Evidence from a panel survey in Liberia. Journal of Peace Research, 54(2), 298-312. | Wrong Design |
| 1. Bosse, T., Gerritsen, C., & de Man, J. (2016). An intelligent system for aggression de-escalation training. In ECAI 2016 (pp. 1805-1811). IOS Press. | Wrong Focus |
| 1. Bowen, D. A., Mercer Kollar, L. M., Wu, D. T., Fraser, D. A., Flood, C. E., Moore, J. C., ... & Sumner, S. A. (2018). Ability of crime, demographic and business data to forecast areas of increased violence. International journal of injury control and safety promotion, 25(4), 443-448. | Wrong Focus |
| 1. Burtsev, M. S. (2005, September). Artificial life meets anthropology: A case of aggression in primitive societies. In European Conference on Artificial Life (pp. 655-664). Springer, Berlin, Heidelberg. | Wrong Focus |
| 1. Canton Croda, R. M., & Gibaja Romero, D. E. (2018). Development of injuries prevention policies in mexico: A big data approach. | Wrong Focus |
| 1. Cederman, L. E., & Weidmann, N. B. (2017). Predicting armed conflict: Time to adjust our expectations?. Science, 355(6324), 474-476. | Wrong Focus |
| 1. Chancellor, S., Sumner, S. A., David-Ferdon, C., Ahmad, T., & De Choudhury, M. (2021). Suicide risk and protective factors in online support forum posts: annotation scheme development and validation study. JMIR mental health, 8(11), e24471. | Wrong Population |
| 1. Chen, I. Y., Alsentzer, E., Park, H., Thomas, R., Gosangi, B., Gujrathi, R., & Khurana, B. (2020). Intimate partner violence and injury prediction from radiology reports. In BIOCOMPUTING 2021: Proceedings of the Pacific Symposium (pp. 55-66). | Wrong Focus |
| 1. Chimeno, S. G., Fernández, J. D., Sánchez, S. M., Ramón, P. P., Ospina, Ó. M. S., Muñoz, M. V., & Hernández, A. G. (2018, June). Domestic Violence Prevention System. In International Symposium on Distributed Computing and Artificial Intelligence (pp. 10-14). Springer, Cham. | Wrong Focus |
| 1. Choi, J. (2022). Identifying Important Factors to Prevent Juvenile Delinquency among Male and Female Adolescents: an Exploratory Analysis Using the LASSO Regression Algorithm in the Korean Children and Youth Panel Survey (KCYPS). Child Indicators Research, 1-36. | Wrong Population |
| 1. Chudova, N. V., & Panov, A. I. (2017). Causal Inference in Psychological Data in the Case of Aggression. Scientific and Technical Information Processing, 44(6), 424-429. | Wrong Focus |
| 1. Chukhrii, I., Zaplatynska, A., Komar, T., Melnyk, Y., Liesnichenko, N., & Nemash, L. (2021). Neuropsychological Approach to the Problem of Aggressive Manifestations of Personality. BRAIN. Broad Research in Artificial Intelligence and Neuroscience, 12(3), 11-24. | Wrong Design |
| 1. Clancy, K., Chudzik, J., Snowden, A. J., & Guha, S. (2022). Reconciling data-driven crime analysis with human-centered algorithms. Cities, 124, 103604. | Wrong Focus |
| 1. Cockerill, R. G. (2020). Ethics Implications of the Use of Artificial Intelligence in Violence Risk Assessment. The journal of the American Academy of Psychiatry and the Law, 48(3), 345-349. | Wrong Focus |
| 1. Constantinou, A. C., Freestone, M., Marsh, W., & Coid, J. (2015). Causal inference for violence risk management and decision support in forensic psychiatry. Decision Support Systems, 80, 42-55. | Wrong Focus |
| 1. Contardo, P., Sernani, P., Falcionelli, N., & Dragoni, A. F. (2021). Deep Learning for Law Enforcement: A Survey About Three Application Domains. In RTA-CSIT (pp. 36-45). | Wrong Population |
| 1. Cope, L. M., Ermer, E., Gaudet, L. M., Steele, V. R., Eckhardt, A. L., Arbabshirani, M. R., ... & Kiehl, K. A. (2014). Abnormal brain structure in youth who commit homicide. Neuroimage: clinical, 4, 800-807. | Wrong Focus |
| 1. Coppersmith, G. A. (2008). A computational investigation into maladaptive aggression. Northeastern University. | Wrong Focus |
| 1. da Silva, A. R. C., de Paula Júnior, I. C., da Silva, T. L. C., de Macêdo, J. A. F., & Silva, W. C. P. (2020, November). Prediction of crime location in a brazilian city using regression techniques. In 2020 IEEE 32nd International Conference on Tools with Artificial Intelligence (ICTAI) (pp. 331-336). IEEE. | Wrong Population |
| 1. Danielsen, A. A., Fenger, M. H. J., Østergaard, S. D., Nielbo, K. L., & Mors, O. (2019). Predicting mechanical restraint of psychiatric inpatients by applying machine learning on electronic health data. Acta Psychiatrica Scandinavica, 140(2), 147-157. | Wrong Focus |
| 1. Dankert, H., Wang, L., Hoopfer, E. D., Anderson, D. J., & Perona, P. (2009). Automated monitoring and analysis of social behavior in Drosophila. Nature methods, 6(4), 297-303. | Wrong Population |
| 1. Dorsey, S. (2020). Machine Learning and Security Studies (Doctoral dissertation, Duke University). | Wrong Focus |
| 1. Durães, D., Marcondes, F. S., Gonçalves, F., Fonseca, J., Machado, J., & Novais, P. (2020, June). Detection violent behaviors: a survey. In International Symposium on Ambient Intelligence (pp. 106-116). Springer, Cham. | Wrong Focus |
| 1. Fitzpatrick, D. J., Gorr, W. L., & Neill, D. B. (2019). Keeping score: Predictive analytics in policing. Annual Review of Criminology, 2(1), 473-491. | Wrong Focus |
| 1. Fridman, N., Kaminka, G. A., & Zilka, A. (2011, May). Towards qualitative reasoning for policy decision support in demonstrations. In International Conference on Autonomous Agents and Multiagent Systems (pp. 19-34). Springer, Berlin, Heidelberg. | Wrong Focus |
| 1. Fridman, N., Zilberstein, T., & Kaminka, G. A. (2011, March). Predicting demonstrations’ violence level using qualitative reasoning. In International Conference on Social Computing, Behavioral-Cultural Modeling, and Prediction (pp. 42-50). Springer, Berlin, Heidelberg. | Wrong Focus |
| 1. Ganesh, R. S. (2021, January). Prediction based on social media dataset using CNN-LSTM to classify the accurate Aggression level. In 2021 International Conference on Computer Communication and Informatics (ICCCI) (pp. 1-4). IEEE. | Wrong Population |
| 1. Garb, H. N., & Wood, J. M. (2019). Methodological advances in statistical prediction. Psychological assessment, 31(12), 1456. | Wrong Focus |
| 1. Gardner-Hoag, J., Novack, M., Parlett-Pelleriti, C., Stevens, E., Dixon, D., & Linstead, E. (2021). Unsupervised machine learning for identifying challenging behavior profiles to explore cluster-based treatment efficacy in children with autism spectrum disorder: Retrospective data analysis study. JMIR Medical Informatics, 9(6), e27793. | Wrong Population |
| 1. Giannini, A. (2021). Lombroso 2.0: On AI and Predictions of Dangerousness in Criminal Justice\|. RIDP, 92(1), 179. | Wrong Focus |
| 1. Goin, D. E., Rudolph, K. E., & Ahern, J. (2018). Predictors of firearm violence in urban communities: a machine-learning approach. Health & place, 51, 61-67. | Wrong Focus |
| 1. Goldstick, J. E., Carter, P. M., Walton, M. A., Dahlberg, L. L., Sumner, S. A., Zimmerman, M. A., & Cunningham, R. M. (2017). Development of the SaFETy score: a clinical screening tool for predicting future firearm violence risk. Annals of internal medicine, 166(10), 707-714. | Wrong Population |
| 1. Goodwin, N. L., Nilsson, S. R., & Golden, S. A. (2020). Rage Against the Machine: Advancing the study of aggression ethology via machine learning. Psychopharmacology, 237(9), 2569-2588. | Wrong Population |
| 1. Goswami, R., Dufort, P., Tartaglia, M. C., Green, R. E., Crawley, A., Tator, C. H., ... & Davis, K. D. (2016). Frontotemporal correlates of impulsivity and machine learning in retired professional athletes with a history of multiple concussions. Brain structure and function, 221(4), 1911-1925. | Wrong Population |
| 1. Greenawald, B., Liu, Y., Wert, G., Al Boni, M., & Brown, D. E. (2018, April). A comparison of language-dependent and language-independent models for violence prediction. In 2018 Systems and Information Engineering Design Symposium (SIEDS) (pp. 260-265). IEEE. | Wrong Focus |
| 1. Griffey, M. F., Barzman, D. H., Ni, Y., Bachtel, A., Dighamber, S., Sorter, M. T., ... & Lin, K. (2018, October). Development of Artificial Intelligence to Predict School Violence. In 65th Annual Meeting. AACAP. | Wrong Population |
| 1. Griffith, J. J., Meyer, D., Maguire, T., Ogloff, J. R., & Daffern, M. (2021). A clinical decision support system to prevent aggression and reduce restrictive practices in a forensic mental health service. Psychiatric services, 72(8), 885-890. | Wrong Focus |
| 1. Günther, M. P., Kirchebner, J., & Lau, S. (2020). Identifying direct coercion in a high risk subgroup of offender patients with schizophrenia via machine learning algorithms. Frontiers in psychiatry, 11, 415. | Wrong Focus |
| 1. Gupta, J., Koprinska, I., & Patrick, J. (2015). Automated classification of clinical incident types. eather Gr, 87. | Wrong Focus |
| 1. Haarsma, G., Davenport, S., White, D. C., Ormachea, P. A., Sheena, E., & Eagleman, D. M. (2020). Assessing risk among correctional community probation populations: Predicting reoffense with mobile neurocognitive assessment software. Frontiers in Psychology, 10, 2926. | Wrong Population |
| 1. Hamilton, Z., Neuilly, M. A., Lee, S., & Barnoski, R. (2015). Isolating modeling effects in offender risk assessment. Journal of Experimental Criminology, 11(2), 299-318. | Wrong Population |
| 1. Hammami, S. M., & Alhammami, M. (2020). Vision-based system model for detecting violence against children. MethodsX, 7, 100744. | Wrong Focus |
| 1. HASHIM, R. T., HAREF, Q. M., MAJEED, H. S., & NSAIF, W. S. (2019). Identify and analyze the relationship between the Proportions of secretions of testosterone hormones to. International Journal of Pharmaceutical Research, 11(4). | Wrong Population |
| 1. He, J., & Zheng, H. (2021). Prediction of crime rate in urban neighborhoods based on machine learning. Engineering Applications of Artificial Intelligence, 106, 104460. | Wrong Population |
| 1. Hegre, H., Allansson, M., Basedau, M., Colaresi, M., Croicu, M., Fjelde, H., ... & Vestby, J. (2019). ViEWS: A political violence early-warning system. Journal of peace research, 56(2), 155-174. | Wrong Focus |
| 1. Hofmann, L. A., Lau, S., & Kirchebner, J. (2022). Advantages of Machine Learning in Forensic Psychiatric Research—Uncovering the Complexities of Aggressive Behavior in Schizophrenia. Applied Sciences, 12(2), 819. | Duplicating same data |
| 1. Hogan, N. R., Davidge, E. Q., & Corabian, G. (2021). On the ethics and practicalities of artificial intelligence, risk assessment, and race. Journal of the American Academy of Psychiatry and the Law. | Wrong Focus |
| 1. Homan, C. M., Schrading, J. N., Ptucha, R. W., Cerulli, C., & Alm, C. O. (2020). Quantitative methods for analyzing intimate partner violence in microblogs: observational study. Journal of medical internet research, 22(11), e15347. | Wrong Focus |
| 1. Hong, W., Kennedy, A., Burgos-Artizzu, X. P., Zelikowsky, M., Navonne, S. G., Perona, P., & Anderson, D. J. (2015). Automated measurement of mouse social behaviors using depth sensing, video tracking, and machine learning. Proceedings of the National Academy of Sciences, 112(38), E5351-E5360. | Wrong Focus |
| 1. Hossain, M. M., Asadullah, M., Rahaman, A., Miah, M. S., Hasan, M. Z., Paul, T., & Hossain, M. A. (2021). Prediction on domestic violence in bangladesh during the covid-19 outbreak using machine learning methods. Applied System Innovation, 4(4), 77. | Wrong Focus |
| 1. Hotzy, F., Theodoridou, A., Hoff, P., Schneeberger, A. R., Seifritz, E., Olbrich, S., & Jäger, M. (2018). Machine learning: An approach in identifying risk factors for coercion compared to binary logistic regression. Frontiers in psychiatry, 9, 258. | Wrong Focus |
| 1. Hu, H., Huang, X., Suhaim, M. A., & Zhang, H. (2021). Comparison of compression estimations under the penalty functions of different violent crimes on campus through deep learning and linear spatial autoregressive models. Applied Mathematics and Nonlinear Sciences. | Wrong Focus |
| 1. Huber, D. A., Lau, S., Sonnweber, M., Günther, M. P., & Kirchebner, J. (2020). Exploring similarities and differences of non-European migrants among forensic patients with schizophrenia. International Journal of Environmental Research and Public Health, 17(21), 7922. | Duplicating same data |
| 1. Hunt, X., Tomlinson, M., Sikander, S., Skeen, S., Marlow, M., du Toit, S., & Eisner, M. (2020). Artificial intelligence, big data, and mHealth: The frontiers of the prevention of violence against children. Frontiers in artificial intelligence, 3, 543305. | Wrong Focus |
| 1. Ingram, K. M., Espelage, D. L., Davis, J. P., & Merrin, G. J. (2020). Family violence, sibling, and peer aggression during adolescence: associations with behavioral health outcomes. Frontiers in psychiatry, 26. | Wrong Population |
| 1. Jahangiri, A., Rakha, H., & Dingus, T. A. (2016). Red-light running violation prediction using observational and simulator data. Accident Analysis & Prevention, 96, 316-328. | Wrong Focus |
| 1. Jatobá, A., Bellas, H. C., Koster, I., Burns, C. M., Vidal, M. C. R., Grecco, C. H. S., & de Carvalho, P. V. R. (2018). Supporting decision-making in patient risk assessment using a hierarchical fuzzy model. Cognition, Technology & Work, 20(3), 477-488. | Wrong Focus |
| 1. Jung, J. S., Park, S. J., Kim, E. Y., Na, K. S., Kim, Y. J., & Kim, K. G. (2019). Prediction models for high risk of suicide in Korean adolescents using machine learning techniques. PLoS one, 14(6), e0217639. | Wrong Population |
| 1. Kaati, L., Shrestha, A., & Sardella, T. (2016, December). Identifying warning behaviors of violent lone offenders in written communication. In 2016 IEEE 16th international conference on data mining workshops (ICDMW) (pp. 1053-1060). IEEE. | Wrong Population |
| 1. Kadar, C., & Pletikosa, I. (2018). Mining large-scale human mobility data for long-term crime prediction. EPJ Data Science, 7(1), 1-27. | Wrong Population |
| 1. Karystianis, G., Cabral, R. C., Han, S. C., Poon, J., & Butler, T. (2021). Utilizing text mining, data linkage and deep learning in police and health records to predict future offenses in family and domestic violence. Frontiers in digital health, 3, 602683. | Wrong Focus |
| 1. Kaur, B., Ahuja, L., & Kumar, V. (2019, February). Crime against women: analysis and prediction using data mining techniques. In 2019 International Conference on Machine Learning, Big Data, Cloud and Parallel Computing (COMITCon) (pp. 194-196). IEEE. | Wrong Design |
| 1. Kazdin, A. E. (2018). Developing treatments for antisocial behavior among children: controlled trials and uncontrolled tribulations. Perspectives on psychological science, 13(5), 634-650. | Wrong Population |
| 1. Kessler, R. C., Warner, C. H., Ivany, C., Petukhova, M. V., Rose, S., Bromet, E. J., ... & Army STARRS Collaborators. (2015). Predicting suicides after psychiatric hospitalization in US Army soldiers: the Army Study to Assess Risk and Resilience in Servicemembers (Army STARRS). JAMA psychiatry, 72(1), 49-57. | Wrong Focus |
| 1. Khan, S. S., Zhu, T., Ye, B., Mihailidis, A., Iaboni, A., Newman, K., ... & Martin, L. S. (2017, November). Daad: A framework for detecting agitation and aggression in people living with dementia using a novel multi-modal sensor network. In 2017 IEEE International Conference on Data Mining Workshops (ICDMW) (pp. 703-710). IEEE. | Wrong Design |
| 1. Khatun, M. R., Ayon, S. I., Hossain, M. R., & Alam, M. J. (2021). Data mining technique to analyse and predict crime using crime categories and arrest records. Indonesian Journal of Electrical Engineering and Computer Science, 22(2), 1052-1060. | Wrong Population |
| 1. Khurana, B., Seltzer, S. E., Kohane, I. S., & Boland, G. W. (2020). Making the ‘invisible’visible: transforming the detection of intimate partner violence. BMJ quality & safety, 29(3), 241-244. | Wrong Focus |
| 1. Kianmehr, K., & Alhajj, R. (2006, March). Crime Hot-spots prediction using support vector machine. In IEEE International Conference on Computer Systems and Applications, 2006. (pp. 952-959). IEEE Computer Society. | Wrong Focus |
| 1. Kirchebner, J., Günther, M. P., & Lau, S. (2020). Identifying influential factors distinguishing recidivists among offender patients with a diagnosis of schizophrenia via machine learning algorithms. Forensic science international, 315, 110435. | Duplicating same data |
| 1. Kirchebner, J., Lau, S., & Sonnweber, M. (2021). Escape and absconding among offenders with schizophrenia spectrum disorder–an explorative analysis of characteristics. BMC psychiatry, 21(1), 1-11. | Wrong Focus |
| 1. Kupilik, M., & Witmer, F. (2018). Spatio-temporal violent event prediction using Gaussian process regression. Journal of Computational Social Science, 1(2), 437-451. | Wrong Population |
| 1. Laqueur, H. S. (2016). Explaining, Measuring, and Predicting the Criminal Behavior of High Rate Offenders (Doctoral dissertation, UC Berkeley). | Wrong Focus |
| 1. Laqueur, H. S., & Wintemute, G. J. (2020). Identifying high‐risk firearm owners to prevent mass violence. Criminology & Public Policy, 19(1), 109-127. | Wrong Focus |
| 1. Le Glaz, A., Haralambous, Y., Kim-Dufor, D. H., Lenca, P., Billot, R., Ryan, T. C., ... & Lemey, C. (2021). Machine learning and natural language processing in mental health: systematic review. Journal of Medical Internet Research, 23(5), e15708. | Wrong Focus |
| 1. Lefter, I., Rothkrantz, L. J., Burghouts, G., Yang, Z., & Wiggers, P. (2011, September). Addressing multimodality in overt aggression detection. In International Conference on Text, Speech and Dialogue (pp. 25-32). Springer, Berlin, Heidelberg. | Wrong Focus |
| 1. Liu, Y., Wert, G., Greenawald, B., Al Boni, M., & Brown, D. E. (2018). Predicting Violent Behavior using Language Agnostic Models. In KDIR (pp. 100-107). | Wrong Population |
| 1. Liu, Y., Zhao, S., Yue, X., Muthu, B., & Kumar, R. L. (2021). AI-based framework for risk estimation in workplace. Aggression and Violent Behavior, 101616 | Wrong Focus |
| 1. Lyu, J., & Zhang, J. (2019). BP neural network prediction model for suicide attempt among Chinese rural residents. Journal of affective disorders, 246, 465-473. | Wrong Design |
| 1. Magnowski, S. R., Kick, D., Cook, J., & Kay, B. (2020). Algorithmic Prediction of Restraint and Seclusion in an Inpatient Child and Adolescent Psychiatric Population. Journal of the American Psychiatric Nurses Association, 1078390320971014. | Wrong Focus |
| 1. Mahmood, N., & Khan, M. U. G. (2022). Prediction of Extremist Behaviour and Suicide Bombing from Terrorism Contents Using Supervised Learning. CMC-COMPUTERS MATERIALS & CONTINUA, 70(3), 4411-4428. | Wrong Focus |
| 1. Malone, I. (2022). Recurrent Neural Networks for Conflict Forecasting. International Interactions, 1-20. | Wrong Focus |
| 1. McDougal, L., Dehingia, N., Bhan, N., Singh, A., McAuley, J., & Raj, A. (2021). Opening closed doors: using machine learning to explore factors associated with marital sexual violence in a cross-sectional study from India. BMJ open, 11(12), e053603. | Wrong Focus |
| 1. Mhlanga, F. S., Perry, E. L., & Kirchner, R. (2014, July). Toward a predictive model ecosystem for interpersonal violence (WIP). In Proceedings of the 2014 Summer Simulation Multiconference (pp. 1-8). | Wrong Focus |
| 1. Miron, M., Tolan, S., Gómez, E., & Castillo, C. (2021). Evaluating causes of algorithmic bias in juvenile criminal recidivism. Artificial Intelligence and Law, 29(2), 111-147. | Wrong Population |
| 1. Mishra, R., Park, C., Atique, M., Najafi, B., Barba-Villalobos, G., Nguyen, J., ... & Calarge, C. (2021, December). Artificial Intelligence-Based Prediction of Aggressive Behavior in Children With Attention Deficit Hyperactivity Disorder. In neuropsychopharmacology (vol. 46, no. suppl 1, pp. 136-136). campus, 4 crinan st, london, n1 9xw, England: Springernature. | Wrong Population |
| 1. Moraru, C., Radulescu, I. D., Voinea, A. I., Dobri, M. L., Gabriela, R. Z., & Nechita, P. (2021). Latent Aggression and Impulsive Manifestations of the Psychiatric Patient. Clinical, Legal and Ethical Aspects. BRAIN. Broad Research in Artificial Intelligence and Neuroscience, 11(3Sup1), 220-230. | Wrong Design |
| 1. Morris, N. P. (2021). Digital technologies and coercion in psychiatry. Psychiatric services, 72(3), 302-310. | Wrong Focus |
| 1. Mueller, H., & Rauh, C. (2018). Reading between the lines: Prediction of political violence using newspaper text. American Political Science Review, 112(2), 358-375. | Wrong Population |
| 1. Nabi, R. M., Saeed, S. A. M., & Haron, H. (2020). Artificial intelligence techniques and external factors used in crime forecasting in violence and property: A review. J. Comput. Sci, 16(2), 167-182. | Wrong Focus |
| 1. Nagar, A. R., Bhat, M. R., Sneha Priya, K., & Rajeshwari, K. (2021). A Holistic Study on Approaches to Prevent Sexual Harassment on Twitter. In Machine Learning for Predictive Analysis (pp. 77-85). Springer, Singapore. | Wrong Focus |
| 1. Naifeh, J. A., Mash, H. B. H., Stein, M. B., Fullerton, C. S., Kessler, R. C., & Ursano, R. J. (2019). The Army Study to Assess Risk and Resilience in Servicemembers (Army STARRS): progress toward understanding suicide among soldiers. Molecular psychiatry, 24(1), 34-48. | Wrong Focus |
| 1. Ni, Y., Barzman, D., Bachtel, A., Griffey, M., Osborn, A., & Sorter, M. (2020). Finding warning markers: leveraging natural language processing and machine learning technologies to detect risk of school violence. International journal of medical informatics, 139, 104137. | Wrong Population |
| 1. Obagbuwa, I. C., & Abidoye, A. P. (2021). South Africa crime visualization, trends analysis, and prediction using machine learning linear regression technique. Applied Computational Intelligence and Soft Computing, 2021. | Wrong Population |
| 1. Odintsova, V. V., Roetman, P. J., Ip, H. F., Pool, R., Van der Laan, C. M., Tona, K. D., ... & Boomsma, D. I. (2019). Genomics of human aggression: current state of genome-wide studies and an automated systematic review tool. Psychiatric genetics, 29(5), 170-190. | Wrong Focus |
| 1. Ohayon, M. M. (1995). Mental disorders and violence: building a knowledge base system for predicting future dangerousness. American Journal of Forensic Psychiatry, 16(4), 47-71. | Wrong Focus |
| 1. Ozkan, T., Clipper, S. J., Piquero, A. R., Baglivio, M., & Wolff, K. (2020). Predicting sexual recidivism. Sexual Abuse, 32(4), 375-399. | Wrong Population |
| 1. Özyirmidokuz, E. K., & Kaya, Y. (2014). Decision Tree Induction of Emotional Violence against Women. In Proceedings of INTCESS14-International Conference on Education and Social Sciences, 3-5 February 2014-Istanbul, Turkey (pp. 847-856). | Wrong Focus |
| 1. Park, H., Gujrathi, R., Gosangi, B., Thomas, R., Cai, T., Chen, I., ... & Khurana, B. (2022). Longitudinal imaging history in early identification of intimate partner violence. European radiology, 32(4), 2824-2836. | Wrong Focus |
| 1. Paul, C., Sahoo, D., & Bora, P. (2020). Aggression in social media: Detection using machine learning algorithms. International Journal of Scientific and Technology Research, 9(04). | Wrong Focus |
| 1. Pelham, W. E., Petras, H., & Pardini, D. A. (2020). Can machine learning improve screening for targeted delinquency prevention programs?. Prevention science, 21(2), 158-170. | Wrong Focus |
| 1. Perry, C. (2013). Machine learning and conflict prediction: a use case. Stability: International Journal of Security and Development, 2(3), 56. | Wrong Focus |
| 1. Petering, R., Barr, N., Srivastava, A., Onasch-Vera, L., Thompson, N., & Rice, E. (2021). Examining impacts of a peer-based mindfulness and yoga intervention to reduce interpersonal violence among young adults experiencing homelessness. Journal of the Society for Social Work and Research, 12(1), 41-57. | Wrong Focus |
| 1. Pham, G. N., Tran, T. V., Nguyen, H. T., Nguyen, P. H., & Le, B. N. Risk Detection Solution on Road Based on Image Processing and Deep Learning. | Wrong Focus |
| 1. Pinals, D. A. (2021). Violence risk assessment in clinical settings: enduring challenges and evolving lessons. Harvard review of psychiatry, 29(1), 90-93. | Did not provided the needed data |
| 1. Poldrack, R. A., Monahan, J., Imrey, P. B., Reyna, V., Raichle, M. E., Faigman, D., & Buckholtz, J. W. (2018). Predicting violent behavior: what can neuroscience add?. Trends in cognitive sciences, 22(2), 111-123. | Wrong Design |
| 1. Pries, L. K., Lage-Castellanos, A., Delespaul, P., Kenis, G., Luykx, J. J., Lin, B. D., ... & Guloksuz, S. (2019). Estimating exposome score for schizophrenia using predictive modeling approach in two independent samples: the results from the EUGEI study. Schizophrenia Bulletin, 45(5), 960-965. | Wrong Focus |
| 1. Qin, J., Shen, H., Zeng, L. L., Gao, K., Luo, Z., & Hu, D. (2019). Dissociating individual connectome traits using low-rank learning. Brain research, 1722, 146348. | Wrong Focus |
| 1. Quigg, Z., Hughes, K., & Bellis, M. A. (2012). Data sharing for prevention: a case study in the development of a comprehensive emergency department injury surveillance system and its use in preventing violence and alcohol-related harms. Injury Prevention, 18(5), 315-320. | Wrong Focus |
| 1. Quijano-Sánchez, L., Liberatore, F., Rodríguez-Lorenzo, G., Lillo, R. E., & González-Álvarez, J. L. (2021). A twist in Intimate Partner Violence Risk Assessment Tools: Gauging the contribution of exogenous and historical variables. Knowledge-Based Systems, 234, 107586. | Wrong Focus |
| 1. Rahman, M. P., Hoque, A. M. I., Ahmed, M. F., Iftekhirul, I., Alam, A., & Hossain, N. (2021, August). Bangladesh Crime Reports Analysis and Prediction. In 2021 International Conference on Software Engineering & Computer Systems and 4th International Conference on Computational Science and Information Management (ICSECS-ICOCSIM) (pp. 453-458). IEEE. | Wrong Focus |
| 1. Raj, A., Dehingia, N., Singh, A., McAuley, J., & McDougal, L. (2021). Machine learning analysis of non-marital sexual violence in India. EClinicalMedicine, 39, 101046. | Wrong Focus |
| 1. Redfern, J., Sidorov, K., Rosin, P. L., Corcoran, P., Moore, S. C., & Marshall, D. (2020). Association of violence with urban points of interest. PloS one, 15(9), e0239840. | Wrong Focus |
| 1. Rhodes, W. (2013). Machine learning approaches as a tool for effective offender risk prediction. Criminology & Pub. Pol'y, 12, 507. | Wrong Focus |
| 1. Rodríguez-Rodríguez, I., Rodríguez, J. V., Pardo-Quiles, D. J., Heras-González, P., & Chatzigiannakis, I. (2020). Modeling and forecasting gender-based violence through machine learning techniques. Applied Sciences, 10(22), 8244. | Wrong Population |
| 1. Roglio, V. S., Borges, E. N., Rabelo-da-Ponte, F. D., Ornell, F., Scherer, J. N., Schuch, J. B., ... & Kessler, F. H. P. (2020). Prediction of attempted suicide in men and women with crack-cocaine use disorder in Brazil. PloS one, 15(5), e0232242. | Wrong Focus |
| 1. Rosellini, A. J., Monahan, J., Street, A. E., Heeringa, S. G., Hill, E. D., Petukhova, M., ... & Kessler, R. C. (2016). Predicting non-familial major physical violent crime perpetration in the US Army from administrative data. Psychological medicine, 46(2), 303-316. | Wrong Population |
| 1. Rosellini, A. J., Monahan, J., Street, A. E., Hill, E. D., Petukhova, M., Reis, B. Y., ... & Kessler, R. C. (2017). Using administrative data to identify US Army soldiers at high-risk of perpetrating minor violent crimes. Journal of psychiatric research, 84, 128-136. | Wrong Population |
| 1. Rosellini, A. J., Stein, M. B., Benedek, D. M., Bliese, P. D., Chiu, W. T., Hwang, I., ... & Kessler, R. C. (2018). Predeployment predictors of psychiatric disorder‐symptoms and interpersonal violence during combat deployment. Depression and anxiety, 35(11), 1073-1080. | Wrong Population |
| 1. Sánchez-Medina, A. J., Galván-Sánchez, I., & Fernández-Monroy, M. (2020). Applying artificial intelligence to explore sexual cyberbullying behaviour. Heliyon, 6(1), e03218. | Wrong Focus |
| 1. Santamaría-García, H., Baez, S., Aponte-Canencio, D. M., Pasciarello, G. O., Donnelly-Kehoe, P. A., Maggiotti, G., ... & Ibáñez, A. (2021). Uncovering social-contextual and individual mental health factors associated with violence via computational inference. Patterns, 2(2), 100176. | Wrong Population |
| 1. Santos, F., Durães, D., Marcondes, F. S., Lange, S., Machado, J., & Novais, P. (2021, October). Efficient violence detection using transfer learning. In Practical Applications of Agents and Multi-Agent Systems (pp. 65-75). Springer, Cham. | Wrong Design |
| 1. Savitha, N.J., Vedhitha, S., Usmi Mukherjee, Riya Francis, Reena (2020). Cyber bullying prediction for safe environment in social media. Journal of Green Engineering (JGE), (10), 9. | Wrong Population |
| 1. Schiliro, F., Beheshti, A., & Moustafa, N. (2020, September). A novel cognitive computing technique using convolutional networks for automating the criminal investigation process in policing. In Proceedings of SAI Intelligent Systems Conference (pp. 528-539). Springer, Cham. | Wrong Focus |
| 1. Senior, M., Burghart, M., Yu, R., Kormilitzin, A., Liu, Q., Vaci, N., ... & Fazel, S. (2020). Identifying Predictors of Suicide in Severe Mental Illness: A Feasibility Study of a Clinical Prediction Rule (Ox ford M ental I llness and S uicide Tool or OxMIS). Frontiers in psychiatry, 11, 268. | Wrong Focus |
| 1. Shapiro, A. (2017). Reform predictive policing. Nature, 541(7638), 458-460. | Wrong Focus |
| 1. Sil, R., Saha, D., & Roy, A. (2020, December). A study on argument-based analysis of legal model. In International Conference on Innovations in Bio-Inspired Computing and Applications (pp. 449-457). Springer, Cham. | Wrong Focus |
| 1. Skeem, J. L., Manchak, S., & Montoya, L. (2017). Comparing public safety outcomes for traditional probation vs specialty mental health probation. JAMA psychiatry, 74(9), 942-948. | Wrong Focus |
| 1. Sonnweber, M., Lau, S., & Kirchebner, J. (2021). Violent and non-violent offending in patients with schizophrenia: Exploring influences and differences via machine learning. Comprehensive psychiatry, 107, 152238. | Duplicating same data |
| 1. Sorella, S., Vellani, V., Siugzdaite, R., Feraco, P., & Grecucci, A. (2022). Structural and functional brain networks of individual differences in trait anger and anger control: An unsupervised machine learning study. European Journal of Neuroscience, 55(2), 510-527. | Wrong Population |
| 1. Spasojevic, S., Nogas, J., Iaboni, A., Ye, B., Mihailidis, A., Wang, A., ... & Khan, S. S. (2021). A pilot study to detect agitation in people living with dementia using multi-modal sensors. Journal of Healthcare Informatics Research, 5(3), 342-358. | Wrong Focus |
| 1. Spivak, B. L., & Shepherd, S. M. (2020). Machine learning and forensic risk assessment: new frontiers. The Journal of Forensic Psychiatry & Psychology, 31(4), 571-581. | Wrong Focus |
| 1. Suchting, R., Gowin, J. L., Green, C. E., Walss-Bass, C., & Lane, S. D. (2018). Genetic and psychosocial predictors of aggression: variable selection and model building with component-wise gradient boosting. Frontiers in behavioral neuroscience, 12, 89. | Wrong Population |
| 1. Tajeddin, M. (2020, May). Predicting Aggressive Responsive Behaviour Among People with Dementia. In Canadian Conference on Artificial Intelligence (pp. 562-565). Springer, Cham. | Wrong Design |
| 1. Tang, T., Song, H., Jaramillo, B., & Baron, J. (2020). Probability learned neural model for human behavior analysis based on language cognition. Aggression and Violent Behavior, 101536. | Wrong Focus |
| 1. Tao, H., Rahman, M. A., Al-Saffar, A., Zhang, R., Salih, S. Q., Zain, J. M., & Al-Hajri, A. A. M. (2021). Security robot for the prevention of workplace violence using the Non-linear Adaptive Heuristic Mathematical Model. Work, 68(3), 853-861. | Wrong Focus |
| 1. Thaipisutikul, T., Tuarob, S., Pongpaichet, S., Amornvatcharapong, A., & Shih, T. K. (2021, January). Automated classification of criminal and violent activities in Thailand from online news articles. In 2021 13th International Conference on Knowledge and Smart Technology (KST) (pp. 170-175). IEEE. | Wrong Focus |
| 1. Tollenaar, N., & van der Heijden, P. G. (2013). Which method predicts recidivism best?: a comparison of statistical, machine learning and data mining predictive models. Journal of the Royal Statistical Society: Series A (Statistics in Society), 176(2), 565-584. | Wrong Population |
| 1. Torres-Carrión, P., Reátegui, R., Valdiviezo, P., Bustamante, B., & Vaca, S. (2019, December). Application of techniques based on Artificial Intelligence for predicting the consumption of drugs and substances. A Systematic Mapping Review. In International Conference on Applied Technologies (pp. 39-52). Springer, Cham. | Wrong Focus |
| 1. Tortora, L., Meynen, G., Bijlsma, J., Tronci, E., & Ferracuti, S. (2020). Neuroprediction and ai in forensic psychiatry and criminal justice: A neurolaw perspective. Frontiers in psychology, 11, 220. | Wrong Design |
| 1. Tripathi, G., Singh, K., & Vishwakarma, D. K. (2021). Applied convolutional neural network framework for tagging healthcare systems in crowd protest environment. Mathematical Biosciences and Engineering, 18(6), 8727-8757. | Wrong Focus |
| 1. Ullah, F. U. M., Ullah, A., Muhammad, K., Haq, I. U., & Baik, S. W. (2019). Violence detection using spatiotemporal features with 3D convolutional neural network. Sensors, 19(11), 2472. | Wrong Focus |
| 1. Van Le, D., Montgomery, J., Kirkby, K. C., & Scanlan, J. (2018). Risk prediction using natural language processing of electronic mental health records in an inpatient forensic psychiatry setting. *Journal of biomedical informatics*, *86*, 49-58. | Wrong Design |
| 1. van ‘t Wout, E., Pieringer, C., Torres Irribarra, D., Asahi, K., & Larroulet, P. (2021). Machine learning for policing: a case study on arrests in Chile. Policing and society, 31(9), 1036-1050. | Wrong Population |
| 1. Veiga Simão, A. M., Costa Ferreira, P., Pereira, N., Oliveira, S., Paulino, P., Rosa, H., ... & Trancoso, I. (2021). Prosociality in cyberspace: Developing emotion and behavioral regulation to decrease aggressive communication. Cognitive Computation, 13(3), 736-750. | Wrong Population |
| 1. Vivar Vera, J. (2021). La sentencia penal, el juez y el algoritmo:¿ Las nuevas tecnologías serán nuestros próximos jueces?. Revista chilena de derecho y tecnología, 10(1), 231-269. | Wrong Focus |
| 1. Weller, O., Sagers, L., Hanson, C., Barnes, M., Snell, Q., & Tass, E. S. (2021). Predicting suicidal thoughts and behavior among adolescents using the risk and protective factor framework: A large-scale machine learning approach. Plos one, 16(11), e0258535. | Wrong Population |
| 1. Wijenayake, S., Graham, T., & Christen, P. (2018, June). A decision tree approach to predicting recidivism in domestic violence. In Pacific-Asia Conference on Knowledge Discovery and Data Mining (pp. 3-15). Springer, Cham. | Wrong Population |
| 1. Xue, J., Chen, J., Chen, C., Hu, R., & Zhu, T. (2020). The hidden pandemic of family violence during COVID-19: unsupervised learning of tweets. Journal of medical Internet research, 22(11), e24361. | Wrong Population |
| 1. Yin, X., Ma, D., Zhu, K., & Li, D. (2021). Identifying intentional injuries among children and adolescents based on Machine Learning. PLoS one, 16(1), e0245437. | Wrong Population |
| 1. Zeng, J., Ustun, B., & Rudin, C. (2017). Interpretable classification models for recidivism prediction. Journal of the Royal Statistical Society: Series A (Statistics in Society), 180(3), 689-722. | Wrong Population |
| 1. Zhou, J., Li, Z., Ma, J. J., & Jiang, F. (2020). Exploration of the hidden influential factors on crime activities: a big data approach. IEEE Access, 8, 141033-141045. | Wrong Focus |

# Probast Quality

**Supplemental Data File 3**

Quality Evaluation **PROBAST – First evaluator**

(Prediction model study Risk Of Bias Assessment Tool)

Published in Annals of Internal Medicine (freely available):

1. [PROBAST: A Tool to Assess the Risk of Bias and Applicability of Prediction Model Studies](https://annals.org/aim/fullarticle/2719961/probast-tool-assess-risk-bias-applicability-prediction-model-studies)
2. [PROBAST: A Tool to Assess Risk of Bias and Applicability of Prediction Model Studies: Explanation and Elaboration](https://annals.org/aim/fullarticle/2719962/probast-tool-assess-risk-bias-applicability-prediction-model-studies-explanation)

| **What does PROBAST assess?**  PROBAST assesses both the *risk of bias* and *concerns regarding applicability* of a study that evaluates (develops, validates or updates) a multivariable diagnostic or prognostic prediction model. It is designed to assess primary studies included in a systematic review.  *Bias* occurs if systematic flaws or limitations in the design, conduct or analysis of a primary study distort the results. For the purpose of prediction modelling studies, we have defined *risk of bias* to occur when shortcomings in the study design, conduct or analysis lead to systematically distorted estimates of a model’s predictive performance or to an inadequate model to address the research question. Model predictive performance is typically evaluated using calibration, discrimination and sometimes classification measures, and these are likely inaccurately estimated in studies with high risk of bias. *Applicability* refers to the extent to which the prediction model from the primary study matches your systematic review question, for example in terms of the participants, predictors or outcome of interest.  A primary study may include the development and/or validation or update of more than one prediction model. A PROBAST assessment should be completed for each distinct model that is developed, validated or updated (extended) for making individualised predictions. Where a publication assesses multiple prediction models, only complete a PROBAST assessment for those models that meet the inclusion criteria for your systematic review. Please note that subsequent use of the term “model” includes derivatives of models, such as simplified risk scores, nomograms, or recalibrations of models.  PROBAST is not designed for all multivariable diagnostic or prognostic studies. For example, studies using multivariable models to identify predictors associated with an outcome but not attempting to develop a model for making individualised predictions are not covered by PROBAST.  PROBAST includes four steps.   \| **Step** \| **Task** \| **When to complete** \| \| --- \| --- \| --- \| \| **1** \| Specify your systematic review question(s) \| Once per systematic review \| \| **2** \| Classify the type of prediction model evaluation \| Once for each model of interest in each publication being assessed, for each relevant outcome \| \| **3** \| Assess risk of bias and applicability \| Once for each development and validation of each distinct prediction model in a publication \| \| **4** \| Overall judgment \| Once for each development and validation of each distinct prediction model in a publication \|   If this is your first time using PROBAST, we strongly recommend reading the detailed explanation and elaboration (E&E, see link above) paper and to check the examples on www.probast.org |
| --- | --- | --- | --- | --- | --- | --- | --- | --- | --- | --- | --- | --- | --- | --- | --- |

**Step 1: Specify your systematic review question**

| State your systematic review question to facilitate the assessment of the applicability of the evaluated models to your question. *The following table should be completed once per systematic review.* |
| --- |

| **Criteria** | **Specify your systematic review question** |
| --- | --- |
| *Intended use of model:* | ML models for predicting risk of aggression and/or violence |
| ***Participants*** *including selection criteria and setting:* | Psychiatric patients in clinical and forensic settings |
| ***Predictors*** *(used in prediction modelling), including types of predictors (e.g. history, clinical examination, biochemical markers, imaging tests), time of measurement, specific measurement issues (e.g., any requirements/ prohibitions for specialized equipment):* | Clinical, sociodemographic and historical characteristics |
| *Outcome to be predicted:* | Violent episode |

**Step 2: Classify the type of prediction model evaluation**

| Use the following table to classify the evaluation as model development, model validation or model update, or combination. Different signalling questions apply for different types of prediction model evaluation. If the evaluation does not fit one of these classifications then PROBAST should not be used. |
| --- |

| **Classify the evaluation based on its aim** | | | |
| --- | --- | --- | --- |
| **Type of prediction study** | **PROBAST boxes to complete** | **Tick as appropriate** | **Definition for type of prediction model study** |
| Development only | Development | 🗹 | Prediction model development without external validation. These studies may include internal validation methods, such as bootstrapping and cross-validation techniques. |
| Development and validation | Development and validation | X | Prediction model development combined with external validation in other participants in the same article. |
| Validation only | Validation | X | External validation of existing (previously developed) model in other participants. |

| *This table should be completed once for each publication being assessed and for each relevant outcome in your review.* | |  |
| --- | --- | --- |
| **Publication reference** | Gau et al. Identification of violent patients with schizophrenia using a hybrid machine learning approach at the individual level | |
| **Models of interest** | Combination of specific multimodal neuroimaging and clinical data in ML analysis | |
| **Outcome of interest** | Risk of violence in patients with schizophrenia | |

**Step 3: Assess risk of bias and applicability**

| PROBAST is structured as four key domains. Each domain is judged for risk of bias (low, high or unclear) and includes signalling questions to help make judgements. Signalling questions are rated as yes (Y), probably yes (PY), probably no (PN), no (N) or no information (NI). All signalling questions are phrased so that “yes” indicates absence of bias. Any signalling question rated as “no” or “probably no” flags the potential for bias; you will need to use your judgement to determine whether the domain should be rated as “high”, “low” or “unclear” risk of bias. The guidance document contains further instructions and examples on rating signalling questions and risk of bias for each domain.  The first three domains are also rated for concerns regarding applicability (low/ high/ unclear) to your review question defined above.  *Complete all domains separately for each evaluation of a distinct model. Shaded boxes indicate where signalling questions do not apply and should not be answered.* |
| --- |

| **DOMAIN 1: Participants** | | | |
| --- | --- | --- | --- |
| **A. Risk of Bias** | | | |
| *Describe the sources of data and criteria for participant selection:*  Participants with schizophrenia who were violent offenders (*n* = 42) were recruited from November 2011 to November 2020 from the forensic psychiatry department of the Second Xiangya Hospital of Central South University. Non-violent patients with schizophrenia (*n* = 32) were recruited from the general psychiatric wards of the psychiatry department.  All the patients met the inclusion criteria: (1) male; (2) aged between 18 and 50 years; (3) with an IQ higher than 70; (4) with a diagnosis of schizophrenia confirmed using the International Classification of Diseases Version 10 (ICD-10). Exclusion criteria included comorbidity with any other diagnosis according to the ICD-10 diagnostic criteria, such as intellectual disability, major neurological disorders, history of head trauma (loss of consciousness for more than 5 min), and any MRI contraindication | | | |
|  | | Dev | Val |
| - 1. Were appropriate data sources used, e.g. cohort, RCT or nested case-control study data? | | PY |  |
| - 1. Were all inclusions and exclusions of participants appropriate? | | Y |  |
| **Risk of bias introduced by selection of participants** | **RISK:**  *(low/ high/ unclear)* | **Low** |  |
| *Rationale of bias rating:* | | | |
| 1.1 PY because no consecutive patients recruited. | | | |
| **B. Applicability** | | | |
| *Describe included participants, setting and dates:*  Participants with schizophrenia who were violent offenders (*n* = 42) were recruited from November 2011 to November 2020 from the forensic psychiatry department of the Second Xiangya Hospital of Central South University. non-violent patients with schizophrenia (*n* = 32) were recruited from the general psychiatric wards of the psychiatry department | | | |
| **Concern that the included participants and setting do not match the review question** | **CONCERN:**  *(low/ high/ unclear)* | **High** |  |
| *Rationale of applicability rating:* | | | |
| Included patients appear not fully representative of the population specified in the review question (general psychiatric and forensic patients, and not only affected by schizophrenia). Applicability may be affected by geographical and healthcare setting as patients were all from two single forensic and psychiatric ward in the the Second Xiangya Hospital of Central South University, China. | | | |

| **DOMAIN 2: Predictors** | | | |
| --- | --- | --- | --- |
| **A. Risk of Bias** | | | |
| *List and describe predictors included in the final model, e.g. definition and timing of assessment:*  The multimodal prediction model combined neuroimaging and sociodemographic-clinical predictors of violence:  Education  BPRS-4 activation  BPRS-5 hostility  PCL-SV total score  HCR-20 total score  BIS-11 total score  Grey Matter Volume  Regional Homogeneity  Fractional Anisotropy  The Leave-One-Out Cross Validation (LOOCV) method was adopted. Each sample was in turn used as the test data, and the remaining samples were used as the training set in the LOOCV scheme. | | | |
|  | | Dev | Val |
| - 1. Were predictors defined and assessed in a similar way for all participants? | | Y |  |
| - 1. Were predictor assessments made without knowledge of outcome data? | | N |  |
| - 1. Are all predictors available at the time the model is intended to be used? | | Y |  |
| **Risk of bias introduced by predictors or their assessment** | **RISK:**  *(low/ high/ unclear)* | **High** |  |
| *Rationale of bias rating:*  We assigned 2.2 = N, because the group was already dived into violent and nonviolent patients affected by schizophrenia (The Modified Overt Aggression Scale - MOAS was used to quantify the overall degree of violence around 1 month before the assessment) | | | |
| **B. Applicability** | | | |
| Concern that the definition, assessment or timing of predictors in the model do not match the review question | **CONCERN:**  *(low/ high/ unclear)* | **Low** |  |
| *Rationale of applicability rating:*  Included predictors appear representative of the predictors specified in the review question. | | | |

| **DOMAIN 3: Outcome** | | | |
| --- | --- | --- | --- |
| **A. Risk of Bias** | | | |
| *Describe the outcome, how it was defined and determined, and the time interval between predictor assessment and outcome determination:*  Outcome: “Violent offenders” referred to those who had committed violent crimes, including killing or assaulting other people (interpersonal violence), and were undergoing forensic psychiatric evaluation. The Modified Overt Aggression Scale (MOAS) was used to quantify the overall degree of violence around 1 month before the assessment. The forensic patients had a score of ≥ 3 for item 4 (physical aggression scale); while the non-violent patients had a score of < 2 for item 4 and were free of any severe aggressive act against property and/or themselves. | | | |
|  | | Dev | Val |
| - 1. Was the outcome determined appropriately? | | Y |  |
| - 1. Was a pre-specified or standard outcome definition used? | | Y |  |
| - 1. Were predictors excluded from the outcome definition? | | Y |  |
| - 1. Was the outcome defined and determined in a similar way for all participants? | | Y |  |
| - 1. Was the outcome determined without knowledge of predictor information? | | Y |  |
| - 1. Was the time interval between predictor assessment and outcome determination appropriate? | | Y |  |
| **Risk of bias introduced by the outcome or its determination** | **RISK:**  *(low/ high/ unclear)* | **Low** |  |
| *Rationale of bias rating:*  No issues emerged | | | |
| **B. Applicability** | | | |
| *At what time point was the outcome determined:*  1 month before the assessment  *If a composite outcome was used, describe the relative frequency/distribution of each contributing outcome:*  Not a composite outcome | | | |
| **Concern that the outcome, its definition, timing or determination do not match the review question** | **CONCERN:**  *(low/ high/ unclear)* | **Low** |  |
| *Rationale of applicability rating:*  Outcome is fitting with the review question | | | |

| **DOMAIN 4: Analysis** | | | |
| --- | --- | --- | --- |
| **Risk of Bias** | | | |
| *Describe numbers of participants, number of candidate predictors, outcome events and events per candidate predictor:*  75 participants, 9 predictors, 42 violent offenders | | | |
| *Describe how the model was developed (for example in regards to modelling technique (e.g. survival or logistic modelling), predictor selection, and risk group definition):*  In order to achieve a superior prediction performance, fusion at both feature level and decision-making level were applied to obtain the most relevant individualized information of multi-level features. At the feature fusion level, a multi-stage feature fusion framework was constructed. At the decision-making fusion level, the three-modality fusion features and sociodemographic-clinical features consisting of the scores on the BPRS hostility, the PCL-SV and HCR-20 total scores and education years were combined using a soft voting method to form a precise prediction model for violence in schizophrenia  Finally, we examined the capability of the model by combining the neuroimaging and sociodemographic-clinical features using a soft voting method, which suggested that the classifier was superior to other models, with an ACC of 90.67%, SEN of 90.91%, SPE of 90.48%, and AUC of 0.95 | | | |
| *Describe whether and how the model was validated, either internally (e.g. bootstrapping, cross validation, random split sample) or externally (e.g. temporal validation, geographical validation, different setting, different type of participants):*  In order to better evaluate the classification performance of this classifier, the Leave-One-Out Cross Validation (LOOCV) method was adopted. Each sample was in turn used as the test data, and the remaining samples were used as the training set in the LOOCV scheme. | | | |
| *Describe the performance measures of the model, e.g. (re)calibration, discrimination, (re)classification, net benefit, and whether they were adjusted for optimism:*  Four metrics (i.e., accuracy, sensitivity, specificity and area under the receiver operating characteristic (ROC) curve [AUC]) were further calculated to fairly measure the classification performance of the corresponding method | | | |
| *Describe any participants who were excluded from the analysis:*  One patient was excluded because he was comorbid with substance abuse | | | |
| *Describe missing data on predictors and outcomes as well as methods used for missing data:*  Authors did not describe how the delt with missing data (N = 7 patients) | | | |
|  | | Dev | Val |
| - 1. Were there a reasonable number of participants with the outcome? | | PN |  |
| - 1. Were continuous and categorical predictors handled appropriately? | | Y |  |
| - 1. Were all enrolled participants included in the analysis? | | Y |  |
| - 1. Were participants with missing data handled appropriately? | | NI |  |
| - 1. Was selection of predictors based on univariable analysis avoided? | | Y |  |
| - 1. Were complexities in the data (e.g. censoring, competing risks, sampling of controls) accounted for appropriately? | | NI |  |
| - 1. Were relevant model performance measures evaluated appropriately? | | Y |  |
| - 1. Were model overfitting and optimism in model performance accounted for? | | Y |  |
| - 1. Do predictors and their assigned weights in the final model correspond to the results from multivariable analysis? | | NI |  |
| **Risk of bias introduced by the analysis** | **RISK:**  *(low/ high/ unclear)* | **High** |  |
| *Rationale of bias rating:*  At least 9 different candidate predictors considered but only 42 participants within an event | | | |

**Step 4: Overall assessment**

| Use the following tables to reach overall judgements about risk of bias and concerns regarding applicability of the prediction model evaluation (development and/or validation) across all assessed domains.  *Complete for each evaluation of a distinct model.*   \| **Reaching an overall judgement about risk of bias of the prediction model evaluation** \| \| \| --- \| --- \| \| **Low risk of bias** \| If all domains were rated low risk of bias.  If a prediction model was developed without any external validation, and it was rated as low risk of bias for all domains, consider downgrading to **high risk of bias**. Such a model can only be considered as low risk of bias, if the development was based on a very large data set and included some form of internal validation. \| \| **High risk of bias** \| If at least one domain is judged to be at **high risk of bias**. \| \| **Unclear risk of bias** \| If an unclear risk of bias was noted in at least one domain and it was low risk for all other domains. \|  \| **Reaching an overall judgement about applicability of the prediction model evaluation** \| \| \| --- \| --- \| \| **Low concerns regarding applicability** \| If low concerns regarding applicability for all domains, the prediction model evaluation is judged to have **low concerns regarding applicability**. \| \| **High concerns regarding applicability** \| If high concerns regarding applicability for at least one domain, the prediction model evaluation is judged to have **high concerns regarding applicability**. \| \| **Unclear concerns regarding applicability** \| If unclear concerns (but no “high concern”) regarding applicability for at least one domain, the prediction model evaluation is judged to have **unclear concerns regarding applicability** overall. \| |
| --- | --- | --- | --- | --- | --- | --- | --- | --- | --- | --- | --- | --- | --- | --- | --- | --- |

| **Overall judgement about risk of bias and applicability of the prediction model evaluation** | | |
| --- | --- | --- |
| **Overall judgement of risk of bias** | **RISK:**  *(low/ high/ unclear)* | **High** |
| *Summary of sources of potential bias:*  Predictor assessment was made knowing the outcome data, and the sample was too small for too many predictors | | |
| **Overall judgement of applicability** | **CONCERN:**  *(low/ high/ unclear)* | **High** |
| *Summary of applicability concerns:*  Included patients appear not fully representative of the population specified in the review question | | |

**PROBAST – First evaluator**

(Prediction model study Risk Of Bias Assessment Tool)

Published in Annals of Internal Medicine (freely available):

1. [PROBAST: A Tool to Assess the Risk of Bias and Applicability of Prediction Model Studies](https://annals.org/aim/fullarticle/2719961/probast-tool-assess-risk-bias-applicability-prediction-model-studies)
2. [PROBAST: A Tool to Assess Risk of Bias and Applicability of Prediction Model Studies: Explanation and Elaboration](https://annals.org/aim/fullarticle/2719962/probast-tool-assess-risk-bias-applicability-prediction-model-studies-explanation)

| **What does PROBAST assess?**  PROBAST assesses both the *risk of bias* and *concerns regarding applicability* of a study that evaluates (develops, validates or updates) a multivariable diagnostic or prognostic prediction model. It is designed to assess primary studies included in a systematic review.  *Bias* occurs if systematic flaws or limitations in the design, conduct or analysis of a primary study distort the results. For the purpose of prediction modelling studies, we have defined *risk of bias* to occur when shortcomings in the study design, conduct or analysis lead to systematically distorted estimates of a model’s predictive performance or to an inadequate model to address the research question. Model predictive performance is typically evaluated using calibration, discrimination and sometimes classification measures, and these are likely inaccurately estimated in studies with high risk of bias. *Applicability* refers to the extent to which the prediction model from the primary study matches your systematic review question, for example in terms of the participants, predictors or outcome of interest.  A primary study may include the development and/or validation or update of more than one prediction model. A PROBAST assessment should be completed for each distinct model that is developed, validated or updated (extended) for making individualised predictions. Where a publication assesses multiple prediction models, only complete a PROBAST assessment for those models that meet the inclusion criteria for your systematic review. Please note that subsequent use of the term “model” includes derivatives of models, such as simplified risk scores, nomograms, or recalibrations of models.  PROBAST is not designed for all multivariable diagnostic or prognostic studies. For example, studies using multivariable models to identify predictors associated with an outcome but not attempting to develop a model for making individualised predictions are not covered by PROBAST.  PROBAST includes four steps.   \| **Step** \| **Task** \| **When to complete** \| \| --- \| --- \| --- \| \| **1** \| Specify your systematic review question(s) \| Once per systematic review \| \| **2** \| Classify the type of prediction model evaluation \| Once for each model of interest in each publication being assessed, for each relevant outcome \| \| **3** \| Assess risk of bias and applicability \| Once for each development and validation of each distinct prediction model in a publication \| \| **4** \| Overall judgment \| Once for each development and validation of each distinct prediction model in a publication \|   If this is your first time using PROBAST, we strongly recommend reading the detailed explanation and elaboration (E&E, see link above) paper and to check the examples on www.probast.org |
| --- | --- | --- | --- | --- | --- | --- | --- | --- | --- | --- | --- | --- | --- | --- | --- |

**Step 1: Specify your systematic review question**

| State your systematic review question to facilitate the assessment of the applicability of the evaluated models to your question. *The following table should be completed once per systematic review.* |
| --- |

| **Criteria** | **Specify your systematic review question** |
| --- | --- |
| *Intended use of model:* | ML models for predicting risk of aggression and/or violence |
| ***Participants*** *including selection criteria and setting:* | Psychiatric patients in clinical and forensic settings |
| ***Predictors*** *(used in prediction modelling), including types of predictors (e.g. history, clinical examination, biochemical markers, imaging tests), time of measurement, specific measurement issues (e.g., any requirements/ prohibitions for specialized equipment):* | Clinical, sociodemographic and historical characteristics |
| *Outcome to be predicted:* | Violent episode |

**Step 2: Classify the type of prediction model evaluation**

| Use the following table to classify the evaluation as model development, model validation or model update, or combination. Different signalling questions apply for different types of prediction model evaluation. If the evaluation does not fit one of these classifications then PROBAST should not be used. |
| --- |

| **Classify the evaluation based on its aim** | | | |
| --- | --- | --- | --- |
| **Type of prediction study** | **PROBAST boxes to complete** | **Tick as appropriate** | **Definition for type of prediction model study** |
| Development only | Development | 🗹 | Prediction model development without external validation. These studies may include internal validation methods, such as bootstrapping and cross-validation techniques. |
| Development and validation | Development and validation | X | Prediction model development combined with external validation in other participants in the same article. |
| Validation only | Validation | X | External validation of existing (previously developed) model in other participants. |

| *This table should be completed once for each publication being assessed and for each relevant outcome in your review.* | |  |
| --- | --- | --- |
| **Publication reference** | Kirchebner et al. Stress, Schizophrenia, and Violence: A Machine Learning Approach | |
| **Models of interest** | Support vector machine, trees, logistic regression | |
| **Outcome of interest** | Study risk factors that lead to violent offenses | |

**Step 3: Assess risk of bias and applicability**

| PROBAST is structured as four key domains. Each domain is judged for risk of bias (low, high or unclear) and includes signalling questions to help make judgements. Signalling questions are rated as yes (Y), probably yes (PY), probably no (PN), no (N) or no information (NI). All signalling questions are phrased so that “yes” indicates absence of bias. Any signalling question rated as “no” or “probably no” flags the potential for bias; you will need to use your judgement to determine whether the domain should be rated as “high”, “low” or “unclear” risk of bias. The guidance document contains further instructions and examples on rating signalling questions and risk of bias for each domain.  The first three domains are also rated for concerns regarding applicability (low/ high/ unclear) to your review question defined above.  *Complete all domains separately for each evaluation of a distinct model. Shaded boxes indicate where signalling questions do not apply and should not be answered.* |
| --- |

| **DOMAIN 1: Participants** | | | |
| --- | --- | --- | --- |
| **A. Risk of Bias** | | | |
| *Describe the sources of data and criteria for participant selection:*  The sample comprised offenders (*n* = 370) who were hospitalized in the Centre for Inpatient Forensic Therapy at the Zurich University Hospital of Psychiatry with a diagnosis of a SSD including schizophrenia, schizoaffective disorder, and delusional disorder given by their psychiatrist at discharge. The data were collected from the inpatients’ medical records from 1982 to 2016. | | | |
|  | | Dev | Val |
| - 1. Were appropriate data sources used, e.g. cohort, RCT or nested case-control study data? | | N |  |
| - 1. Were all inclusions and exclusions of participants appropriate? | | NI |  |
| **Risk of bias introduced by selection of participants** | **RISK:**  *(low/ high/ unclear)* | **High** |  |
| *Rationale of bias rating:* | | | |
| 1.1 is N because the design is retrospective, consequently data were collected for other purposes. | | | |
| **B. Applicability** | | | |
| *Describe included participants, setting and dates:*  The sample comprised offenders (n = 370) who were hospitalized in the Centre for Inpatient Forensic Therapy at the Zurich University Hospital of Psychiatry with a diagnosis of a SSD including schizophrenia, schizoaffective disorder, and delusional disorder given by their psychiatrist at discharge | | | |
| **Concern that the included participants and setting do not match the review question** | **CONCERN:**  *(low/ high/ unclear)* | **High** |  |
| *Rationale of applicability rating:* | | | |
| Included patients appear not fully representative of the population specified in the review question (general psychiatric and forensic patients, and not only affected by schizophrenia spectrum disorders. Applicability may be affected by geographical and healthcare setting as patients were all from two single forensic and psychiatric ward in in the Centre for Inpatient Forensic Therapy at the Zurich University Hospital of Psychiatry, Switzerland. | | | |

| **DOMAIN 2: Predictors** | | | |
| --- | --- | --- | --- |
| **A. Risk of Bias** | | | |
| *List and describe predictors included in the final model, e.g. definition and timing of assessment:*  For selecting predictors, authors closely followed the existing literature on the GST and the stressors discussed by Agnew. They classified the stressors into three categories: childhood and adolescence, adulthood, and psychiatric stressors (PS).  **Stressors in childhood/youth**  bullying  separation/divorce of caregivers  impairment of the parent–child relationship  physical abuse by the caregiver  sexual abuse by the caregiver  poverty  separation from caregiver  rejection/being ignored by the caregiver active devaluation by the caregiver  poor parenting methods  violent physical illness of the patient  failure in school  **Stressors in adulthood**  Unemployment (at time of offense) homelessness  conflicts in the workplace  social isolation  violent victimization  **Psychiatric stressors**  coercive psychiatric treatment  at least three previous hospitalizations  compulsory psychiatric placement  positive symptoms during criminal offense | | | |
|  | | Dev | Val |
| - 1. Were predictors defined and assessed in a similar way for all participants? | | Y |  |
| - 1. Were predictor assessments made without knowledge of outcome data? | | Y |  |
| - 1. Are all predictors available at the time the model is intended to be used? | | Y |  |
| **Risk of bias introduced by predictors or their assessment** | **RISK:**  *(low/ high/ unclear)* | **Low** |  |
| *Rationale of bias rating:*  No issues emerged | | | |
| **B. Applicability** | | | |
| Concern that the definition, assessment or timing of predictors in the model do not match the review question | **CONCERN:**  *(low/ high/ unclear)* | **Low** |  |
| *Rationale of applicability rating:*  Included predictors appear representative of the predictors specified in the review question. | | | |

| **DOMAIN 3: Outcome** | | | |
| --- | --- | --- | --- |
| **A. Risk of Bias** | | | |
| *Describe the outcome, how it was defined and determined, and the time interval between predictor assessment and outcome determination:*  The outcome—severity of the index offense—was dichotomized into (a) violent offense and (b) nonviolent offense.  The following offenses were considered as violent based on Swiss law: homicide and attempted homicide, assault, rape, robbery, arson, and child abuse. The category nonviolent offense included threat, theft, damage to property, minor sexual offenses (e.g., exhibitionism), drug offenses, illegal gun possession, and other minor offenses (e.g., triggering false alarms or emergency brakes). | | | |
|  | | Dev | Val |
| - 1. Was the outcome determined appropriately? | | Y |  |
| - 1. Was a pre-specified or standard outcome definition used? | | Y |  |
| - 1. Were predictors excluded from the outcome definition? | | Y |  |
| - 1. Was the outcome defined and determined in a similar way for all participants? | | Y |  |
| - 1. Was the outcome determined without knowledge of predictor information? | | NI |  |
| - 1. Was the time interval between predictor assessment and outcome determination appropriate? | | Y |  |
| **Risk of bias introduced by the outcome or its determination** | **RISK:**  *(low/ high/ unclear)* | **Low** |  |
| *Rationale of bias rating:*  No issues emerged | | | |
| **B. Applicability** | | | |
| *At what time point was the outcome determined:*  After collecting predictors  *If a composite outcome was used, describe the relative frequency/distribution of each contributing outcome:*  Not a composite outcome | | | |
| **Concern that the outcome, its definition, timing or determination do not match the review question** | **CONCERN:**  *(low/ high/ unclear)* | **Low** |  |
| *Rationale of applicability rating:*  Outcome is fitting with the review question | | | |

| **DOMAIN 4: Analysis** | | | |
| --- | --- | --- | --- |
| **Risk of Bias** | | | |
| *Describe numbers of participants, number of candidate predictors, outcome events and events per candidate predictor:*  The sample comprised offenders (n = 370) with a diagnosis of a SSD including schizophrenia, schizoaffective disorder, and delusional disorder given by their psychiatrist at discharge  21 predictors  294 patients (79.7%) had committed a violent index offense | | | |
| *Describe how the model was developed (for example in regards to modelling technique (e.g. survival or logistic modelling), predictor selection, and risk group definition):*  Algorithm selection and performance testing were conducted using MATLAB (The MathWorks, 2012). Binary logistic regression and evaluation of the variable importance via the selected algorithm were both performed in R Studio version 1.1.383.  To identify stressors that have explanatory power regarding offense severity, a supervised ML with the outcome variable violent offense/nonviolent offense and all 21 possible predictor variables to identify the most accurate algorithm (logistic regression, support vector machine [SVM], trees, and KNN algorithms) was performed. | | | |
| *Describe whether and how the model was validated, either internally (e.g. bootstrapping, cross validation, random split sample) or externally (e.g. temporal validation, geographical validation, different setting, different type of participants):*  The training set was divided into five equal-sized subsets, with one subset being used to train a model and the other four being used to evaluate the accuracy of the learned model (fivefold cross-validation). The model showing the best predictive accuracy was chosen for further analysis. | | | |
| *Describe the performance measures of the model, e.g. (re)calibration, discrimination, (re)classification, net benefit, and whether they were adjusted for optimism:*  The goodness of fit was assessed using the receiver operating characteristic (ROC) curve method. In addition, the specificity and sensitivity, positive predictive value (PPV), and negative predictive value (NPV) were calculated. | | | |
| *Describe any participants who were excluded from the analysis:*  One patient showed missing data on his index offense and was therefore excluded from the study | | | |
| *Describe missing data on predictors and outcomes as well as methods used for missing data:*  No information | | | |
|  | | Dev | Val |
| - 1. Were there a reasonable number of participants with the outcome? | | Y |  |
| - 1. Were continuous and categorical predictors handled appropriately? | | Y |  |
| - 1. Were all enrolled participants included in the analysis? | | N |  |
| - 1. Were participants with missing data handled appropriately? | | Y |  |
| - 1. Was selection of predictors based on univariable analysis avoided? | | Y |  |
| - 1. Were complexities in the data (e.g. censoring, competing risks, sampling of controls) accounted for appropriately? | | PY |  |
| - 1. Were relevant model performance measures evaluated appropriately? | | Y |  |
| - 1. Were model overfitting and optimism in model performance accounted for? | | NI |  |
| - 1. Do predictors and their assigned weights in the final model correspond to the results from multivariable analysis? | | PY |  |
| **Risk of bias introduced by the analysis** | **RISK:**  *(low/ high/ unclear)* | **Low** |  |
| *Rationale of bias rating:*  The frequency of missing data was low, therefore the risk of bias was still considered low. No other problematic issues are present with respect to this domain. | | | |

**Step 4: Overall assessment**

| Use the following tables to reach overall judgements about risk of bias and concerns regarding applicability of the prediction model evaluation (development and/or validation) across all assessed domains.  *Complete for each evaluation of a distinct model.*   \| **Reaching an overall judgement about risk of bias of the prediction model evaluation** \| \| \| --- \| --- \| \| **Low risk of bias** \| If all domains were rated low risk of bias.  If a prediction model was developed without any external validation, and it was rated as low risk of bias for all domains, consider downgrading to **high risk of bias**. Such a model can only be considered as low risk of bias, if the development was based on a very large data set and included some form of internal validation. \| \| **High risk of bias** \| If at least one domain is judged to be at **high risk of bias**. \| \| **Unclear risk of bias** \| If an unclear risk of bias was noted in at least one domain and it was low risk for all other domains. \|  \| **Reaching an overall judgement about applicability of the prediction model evaluation** \| \| \| --- \| --- \| \| **Low concerns regarding applicability** \| If low concerns regarding applicability for all domains, the prediction model evaluation is judged to have **low concerns regarding applicability**. \| \| **High concerns regarding applicability** \| If high concerns regarding applicability for at least one domain, the prediction model evaluation is judged to have **high concerns regarding applicability**. \| \| **Unclear concerns regarding applicability** \| If unclear concerns (but no “high concern”) regarding applicability for at least one domain, the prediction model evaluation is judged to have **unclear concerns regarding applicability** overall. \| |
| --- | --- | --- | --- | --- | --- | --- | --- | --- | --- | --- | --- | --- | --- | --- | --- | --- |

| **Overall judgement about risk of bias and applicability of the prediction model evaluation** | | |
| --- | --- | --- |
| **Overall judgement of risk of bias** | **RISK:**  *(low/ high/ unclear)* | **High** |
| *Summary of sources of potential bias:*  The design is retrospective, consequently data were collected for other purposes. | | |
| **Overall judgement of applicability** | **CONCERN:**  *(low/ high/ unclear)* | **High** |
| *Summary of applicability concerns:*  Included patients appear not fully representative of the population specified in the review question | | |

**PROBAST – First evaluator**

(Prediction model study Risk Of Bias Assessment Tool)

Published in Annals of Internal Medicine (freely available):

1. [PROBAST: A Tool to Assess the Risk of Bias and Applicability of Prediction Model Studies](https://annals.org/aim/fullarticle/2719961/probast-tool-assess-risk-bias-applicability-prediction-model-studies)
2. [PROBAST: A Tool to Assess Risk of Bias and Applicability of Prediction Model Studies: Explanation and Elaboration](https://annals.org/aim/fullarticle/2719962/probast-tool-assess-risk-bias-applicability-prediction-model-studies-explanation)

| **What does PROBAST assess?**  PROBAST assesses both the *risk of bias* and *concerns regarding applicability* of a study that evaluates (develops, validates or updates) a multivariable diagnostic or prognostic prediction model. It is designed to assess primary studies included in a systematic review.  *Bias* occurs if systematic flaws or limitations in the design, conduct or analysis of a primary study distort the results. For the purpose of prediction modelling studies, we have defined *risk of bias* to occur when shortcomings in the study design, conduct or analysis lead to systematically distorted estimates of a model’s predictive performance or to an inadequate model to address the research question. Model predictive performance is typically evaluated using calibration, discrimination and sometimes classification measures, and these are likely inaccurately estimated in studies with high risk of bias. *Applicability* refers to the extent to which the prediction model from the primary study matches your systematic review question, for example in terms of the participants, predictors or outcome of interest.  A primary study may include the development and/or validation or update of more than one prediction model. A PROBAST assessment should be completed for each distinct model that is developed, validated or updated (extended) for making individualised predictions. Where a publication assesses multiple prediction models, only complete a PROBAST assessment for those models that meet the inclusion criteria for your systematic review. Please note that subsequent use of the term “model” includes derivatives of models, such as simplified risk scores, nomograms, or recalibrations of models.  PROBAST is not designed for all multivariable diagnostic or prognostic studies. For example, studies using multivariable models to identify predictors associated with an outcome but not attempting to develop a model for making individualised predictions are not covered by PROBAST.  PROBAST includes four steps.   \| **Step** \| **Task** \| **When to complete** \| \| --- \| --- \| --- \| \| **1** \| Specify your systematic review question(s) \| Once per systematic review \| \| **2** \| Classify the type of prediction model evaluation \| Once for each model of interest in each publication being assessed, for each relevant outcome \| \| **3** \| Assess risk of bias and applicability \| Once for each development and validation of each distinct prediction model in a publication \| \| **4** \| Overall judgment \| Once for each development and validation of each distinct prediction model in a publication \|   If this is your first time using PROBAST, we strongly recommend reading the detailed explanation and elaboration (E&E, see link above) paper and to check the examples on [www.probast.org](http://www.probast.org) |
| --- | --- | --- | --- | --- | --- | --- | --- | --- | --- | --- | --- | --- | --- | --- | --- |

**Step 1: Specify your systematic review question**

| State your systematic review question to facilitate the assessment of the applicability of the evaluated models to your question. *The following table should be completed once per systematic review.* |
| --- |

| **Criteria** | **Specify your systematic review question** |
| --- | --- |
| *Intended use of model:* | ML models for predicting risk of aggression and/or violence |
| ***Participants*** *including selection criteria and setting:* | Psychiatric patients in clinical and forensic settings |
| ***Predictors*** *(used in prediction modelling), including types of predictors (e.g. history, clinical examination, biochemical markers, imaging tests), time of measurement, specific measurement issues (e.g., any requirements/ prohibitions for specialized equipment):* | Clinical, sociodemographic and historical characteristics |
| *Outcome to be predicted:* | Violent episode |

**Step 2: Classify the type of prediction model evaluation**

| Use the following table to classify the evaluation as model development, model validation or model update, or combination. Different signalling questions apply for different types of prediction model evaluation. If the evaluation does not fit one of these classifications then PROBAST should not be used. |
| --- |

| **Classify the evaluation based on its aim** | | | |
| --- | --- | --- | --- |
| **Type of prediction study** | **PROBAST boxes to complete** | **Tick as appropriate** | **Definition for type of prediction model study** |
| Development only | Development | 🗹 | Prediction model development without external validation. These studies may include internal validation methods, such as bootstrapping and cross-validation techniques. |
| Development and validation | Development and validation | X | Prediction model development combined with external validation in other participants in the same article. |
| Validation only | Validation | X | External validation of existing (previously developed) model in other participants. |

| *This table should be completed once for each publication being assessed and for each relevant outcome in your review.* | |  |
| --- | --- | --- |
| **Publication reference** | Lu et al. 2021. Psychosocial Factors Predict the Level of Aggression of People with Drug Addiction: A Machine Learning Approach | |
| **Models of interest** | Gradient Boosting Regression Tree (GBRT), | |
| **Outcome of interest** | To identify the relevant psychosocial factors that can predict the aggression in people with drug addiction | |

**Step 3: Assess risk of bias and applicability**

| PROBAST is structured as four key domains. Each domain is judged for risk of bias (low, high or unclear) and includes signalling questions to help make judgements. Signalling questions are rated as yes (Y), probably yes (PY), probably no (PN), no (N) or no information (NI). All signalling questions are phrased so that “yes” indicates absence of bias. Any signalling question rated as “no” or “probably no” flags the potential for bias; you will need to use your judgement to determine whether the domain should be rated as “high”, “low” or “unclear” risk of bias. The guidance document contains further instructions and examples on rating signalling questions and risk of bias for each domain.  The first three domains are also rated for concerns regarding applicability (low/ high/ unclear) to your review question defined above.  *Complete all domains separately for each evaluation of a distinct model. Shaded boxes indicate where signalling questions do not apply and should not be answered.* |
| --- |

| **DOMAIN 1: Participants** | | | |
| --- | --- | --- | --- |
| **A. Risk of Bias** | | | |
| *Describe the sources of data and criteria for participant selection:*  The participants in this study were recruited from Zengcheng Compulsory Isolated Detoxification Center in Guangdong province, southern China, using cluster sampling. A total of 896 participants (Mean*age* = 38.30, SD = 8.38) participated in the study.  People who meet the following criteria are not eligible for the study: Unable to understand the terms in the questionnaire and Leaving more than 30% items uncompleted. | | | |
|  | | Dev | Val |
| - 1. Were appropriate data sources used, e.g. cohort, RCT or nested case-control study data? | | PN |  |
| - 1. Were all inclusions and exclusions of participants appropriate? | | N |  |
| **Risk of bias introduced by selection of participants** | **RISK:**  *(low/ high/ unclear)* | **High** |  |
| *Rationale of bias rating:*  Cluster sampling is prone to high sampling error. Exclusion criteria: Unable to understand the terms in the questionnaire and Leaving more than 30% items uncompleted. | | | |
| 30% of missing is too high | | | |
| **B. Applicability** | | | |
| *Describe included participants, setting and dates:*  The participants in this study were recruited from Zengcheng Compulsory Isolated Detoxification Center in Guangdong province, southern China, using cluster sampling. A total of 896 participants (Meanage = 38.30, SD = 8.38) participated in the study. People who meet the following criteria are not eligible for the study: Unable to understand the terms in the questionnaire and Leaving more than 30% items uncompleted | | | |
| **Concern that the included participants and setting do not match the review question** | **CONCERN:**  *(low/ high/ unclear)* | **High** |  |
| *Rationale of applicability rating:* | | | |
| Included patients appear not fully representative of the population specified in the review question (general psychiatric and forensic patients, and not only affected by drug addiction). | | | |

| **DOMAIN 2: Predictors** | | | |
| --- | --- | --- | --- |
| **A. Risk of Bias** | | | |
| *List and describe predictors included in the final model, e.g. definition and timing of assessment:*  Drug craving scale (DCS)  Buss-Warren Aggression Questionnaire Revised in China (BWAQ-RC)  Impulsivity scale  Security Questionnaire (SQ)  Positive Psychological Capital Questionnaire (PPCQ)  Toronto Alexithymia Scale (TAS-20)  Children’s Perception of Inter-parental Conflict Scale (CPIC)  Interpersonal Trust Scales (ITS)  Year of birth | | | |
|  | | Dev | Val |
| - 1. Were predictors defined and assessed in a similar way for all participants? | | Y |  |
| - 1. Were predictor assessments made without knowledge of outcome data? | | NI |  |
| - 1. Are all predictors available at the time the model is intended to be used? | | Y |  |
| **Risk of bias introduced by predictors or their assessment** | **RISK:**  *(low/ high/ unclear)* | **Unclear** |  |
| *Rationale of bias rating:*  It is not clear how and when they defined the outcome | | | |
| **B. Applicability** | | | |
| Concern that the definition, assessment or timing of predictors in the model do not match the review question | **CONCERN:**  *(low/ high/ unclear)* | **Low** |  |
| *Rationale of applicability rating:*  Predictors are fitting with the review question | | | |

| **DOMAIN 3: Outcome** | | | |
| --- | --- | --- | --- |
| **A. Risk of Bias** | | | |
| *Describe the outcome, how it was defined and determined, and the time interval between predictor assessment and outcome determination:*  It is not clear how they defined patients’ aggression. | | | |
|  | | Dev | Val |
| - 1. Was the outcome determined appropriately? | | NI |  |
| - 1. Was a pre-specified or standard outcome definition used? | | NI |  |
| - 1. Were predictors excluded from the outcome definition? | | N |  |
| - 1. Was the outcome defined and determined in a similar way for all participants? | | NI |  |
| - 1. Was the outcome determined without knowledge of predictor information? | | NI |  |
| - 1. Was the time interval between predictor assessment and outcome determination appropriate? | | NI |  |
| **Risk of bias introduced by the outcome or its determination** | **RISK:**  *(low/ high/ unclear)* | **Unclear** |  |
| *Rationale of bias rating:*  The definition of the outcome was not clear | | | |
| **B. Applicability** | | | |
| *At what time point was the outcome determined:*  It is not clear  *If a composite outcome was used, describe the relative frequency/distribution of each contributing outcome:*  No composite outcome | | | |
| **Concern that the outcome, its definition, timing or determination do not match the review question** | **CONCERN:**  *(low/ high/ unclear)* | **High** |  |
| *Rationale of applicability rating:*  It is not clear at what point and how was determined the outcome | | | |

| **DOMAIN 4: Analysis** | | | |
| --- | --- | --- | --- |
| **Risk of Bias** | | | |
| Describe numbers of participants, number of candidate predictors, outcome events and events per candidate predictor:  The participants in this study were recruited from Zengcheng Compulsory Isolated Detoxification Center in Guangdong province, southern China, using cluster sampling. A total of 896 participants (Meanage = 38.30, SD = 8.38) participated in the study.  Not specified the exact number of predictors nor the number of outcome events | | | |
| *Describe how the model was developed (for example in regards to modelling technique (e.g. survival or logistic modelling), predictor selection, and risk group definition):*  The present study utilizes the Gradient Boosting Regression Tree (GBRT), a machine learning algorithm, to predict aggression with several psychosocial factors as well as Factor Importance to assess the relative importance of a specific feature according to the predicting contribution of the target variable. | | | |
| *Describe whether and how the model was validated, either internally (e.g. bootstrapping, cross validation, random split sample) or externally (e.g. temporal validation, geographical validation, different setting, different type of participants):*  A fivefold cross-validation or out-of-sample testing techniques was used to estimate the accuracy of the predictive model | | | |
| *Describe the performance measures of the model, e.g. (re)calibration, discrimination, (re)classification, net benefit, and whether they were adjusted for optimism:*  No information | | | |
| *Describe any participants who were excluded from the analysis:*  No information on the exact number. People who meet the following criteria were not eligible for the study: Unable to understand the terms in the questionnaire and Leaving more than 30% items uncompleted. | | | |
| *Describe missing data on predictors and outcomes as well as methods used for missing data:*  No information on missing data number. The authors stated that missing values of the included data were replaced with averages | | | |
|  | | Dev | Val |
| - 1. Were there a reasonable number of participants with the outcome? | | NI |  |
| - 1. Were continuous and categorical predictors handled appropriately? | | NI |  |
| - 1. Were all enrolled participants included in the analysis? | | Y |  |
| - 1. Were participants with missing data handled appropriately? | | Y |  |
| - 1. Was selection of predictors based on univariable analysis avoided? | | Y |  |
| - 1. Were complexities in the data (e.g. censoring, competing risks, sampling of controls) accounted for appropriately? | | NI |  |
| - 1. Were relevant model performance measures evaluated appropriately? | | N |  |
| - 1. Were model overfitting and optimism in model performance accounted for? | | NI |  |
| - 1. Do predictors and their assigned weights in the final model correspond to the results from multivariable analysis? | | NI |  |
| **Risk of bias introduced by the analysis** | **RISK:**  *(low/ high/ unclear)* | **High** |  |
| *Rationale of bias rating:*  No information on number of participants with outcome, nor performance measures of the model | | | |

**Step 4: Overall assessment**

| Use the following tables to reach overall judgements about risk of bias and concerns regarding applicability of the prediction model evaluation (development and/or validation) across all assessed domains.  *Complete for each evaluation of a distinct model.*   \| **Reaching an overall judgement about risk of bias of the prediction model evaluation** \| \| \| --- \| --- \| \| **Low risk of bias** \| If all domains were rated low risk of bias.  If a prediction model was developed without any external validation, and it was rated as low risk of bias for all domains, consider downgrading to **high risk of bias**. Such a model can only be considered as low risk of bias, if the development was based on a very large data set and included some form of internal validation. \| \| **High risk of bias** \| If at least one domain is judged to be at **high risk of bias**. \| \| **Unclear risk of bias** \| If an unclear risk of bias was noted in at least one domain and it was low risk for all other domains. \|  \| **Reaching an overall judgement about applicability of the prediction model evaluation** \| \| \| --- \| --- \| \| **Low concerns regarding applicability** \| If low concerns regarding applicability for all domains, the prediction model evaluation is judged to have **low concerns regarding applicability**. \| \| **High concerns regarding applicability** \| If high concerns regarding applicability for at least one domain, the prediction model evaluation is judged to have **high concerns regarding applicability**. \| \| **Unclear concerns regarding applicability** \| If unclear concerns (but no “high concern”) regarding applicability for at least one domain, the prediction model evaluation is judged to have **unclear concerns regarding applicability** overall. \| |
| --- | --- | --- | --- | --- | --- | --- | --- | --- | --- | --- | --- | --- | --- | --- | --- | --- |

| **Overall judgement about risk of bias and applicability of the prediction model evaluation** | | |
| --- | --- | --- |
| **Overall judgement of risk of bias** | **RISK:**  *(low/ high/ unclear)* | **High** |
| *Summary of sources of potential bias:*  30% of missing is too high  It is not clear how and when they defined the outcome  No information on number of participants with outcome, nor performance measures of the model | | |
| **Overall judgement of applicability** | **CONCERN:**  *(low/ high/ unclear)* | **High** |
| *Summary of applicability concerns:*  Included patients appear not fully representative of the population specified in the review question | | |

**PROBAST – First evaluator**

(Prediction model study Risk Of Bias Assessment Tool)

Published in Annals of Internal Medicine (freely available):

1. [PROBAST: A Tool to Assess the Risk of Bias and Applicability of Prediction Model Studies](https://annals.org/aim/fullarticle/2719961/probast-tool-assess-risk-bias-applicability-prediction-model-studies)
2. [PROBAST: A Tool to Assess Risk of Bias and Applicability of Prediction Model Studies: Explanation and Elaboration](https://annals.org/aim/fullarticle/2719962/probast-tool-assess-risk-bias-applicability-prediction-model-studies-explanation)

| **What does PROBAST assess?**  PROBAST assesses both the *risk of bias* and *concerns regarding applicability* of a study that evaluates (develops, validates or updates) a multivariable diagnostic or prognostic prediction model. It is designed to assess primary studies included in a systematic review.  *Bias* occurs if systematic flaws or limitations in the design, conduct or analysis of a primary study distort the results. For the purpose of prediction modelling studies, we have defined *risk of bias* to occur when shortcomings in the study design, conduct or analysis lead to systematically distorted estimates of a model’s predictive performance or to an inadequate model to address the research question. Model predictive performance is typically evaluated using calibration, discrimination and sometimes classification measures, and these are likely inaccurately estimated in studies with high risk of bias. *Applicability* refers to the extent to which the prediction model from the primary study matches your systematic review question, for example in terms of the participants, predictors or outcome of interest.  A primary study may include the development and/or validation or update of more than one prediction model. A PROBAST assessment should be completed for each distinct model that is developed, validated or updated (extended) for making individualised predictions. Where a publication assesses multiple prediction models, only complete a PROBAST assessment for those models that meet the inclusion criteria for your systematic review. Please note that subsequent use of the term “model” includes derivatives of models, such as simplified risk scores, nomograms, or recalibrations of models.  PROBAST is not designed for all multivariable diagnostic or prognostic studies. For example, studies using multivariable models to identify predictors associated with an outcome but not attempting to develop a model for making individualised predictions are not covered by PROBAST.  PROBAST includes four steps.   \| **Step** \| **Task** \| **When to complete** \| \| --- \| --- \| --- \| \| **1** \| Specify your systematic review question(s) \| Once per systematic review \| \| **2** \| Classify the type of prediction model evaluation \| Once for each model of interest in each publication being assessed, for each relevant outcome \| \| **3** \| Assess risk of bias and applicability \| Once for each development and validation of each distinct prediction model in a publication \| \| **4** \| Overall judgment \| Once for each development and validation of each distinct prediction model in a publication \|   If this is your first time using PROBAST, we strongly recommend reading the detailed explanation and elaboration (E&E, see link above) paper and to check the examples on www.probast.org |
| --- | --- | --- | --- | --- | --- | --- | --- | --- | --- | --- | --- | --- | --- | --- | --- |

**Step 1: Specify your systematic review question**

| State your systematic review question to facilitate the assessment of the applicability of the evaluated models to your question. *The following table should be completed once per systematic review.* |
| --- |

| **Criteria** | **Specify your systematic review question** |
| --- | --- |
| *Intended use of model:* | ML models for predicting risk of aggression and/or violence |
| ***Participants*** *including selection criteria and setting:* | Psychiatric patients in clinical and forensic settings |
| ***Predictors*** *(used in prediction modelling), including types of predictors (e.g. history, clinical examination, biochemical markers, imaging tests), time of measurement, specific measurement issues (e.g., any requirements/ prohibitions for specialized equipment):* | Clinical, sociodemographic and historical characteristics |
| *Outcome to be predicted:* | Violent episode |

**Step 2: Classify the type of prediction model evaluation**

| Use the following table to classify the evaluation as model development, model validation or model update, or combination. Different signalling questions apply for different types of prediction model evaluation. If the evaluation does not fit one of these classifications then PROBAST should not be used. |
| --- |

| **Classify the evaluation based on its aim** | | | |
| --- | --- | --- | --- |
| **Type of prediction study** | **PROBAST boxes to complete** | **Tick as appropriate** | **Definition for type of prediction model study** |
| Development only | Development | 🗹 | Prediction model development without external validation. These studies may include internal validation methods, such as bootstrapping and cross-validation techniques. |
| Development and validation | Development and validation | X | Prediction model development combined with external validation in other participants in the same article. |
| Validation only | Validation | X | External validation of existing (previously developed) model in other participants. |

| *This table should be completed once for each publication being assessed and for each relevant outcome in your review.* | |  |
| --- | --- | --- |
| **Publication reference** | Menger et al. 2018. Comparing Deep Learning and Classical Machine Learning Approaches for Predicting Inpatient Violence Incidents from Clinical Text | |
| **Models of interest** | Recurrent Neural Network, Convolutional Neural Network, Neural Network, Naive Bayes, Support Vector Machine, Decision Tree | |
| **Outcome of interest** | Inpatients violent incidents | |

**Step 3: Assess risk of bias and applicability**

| PROBAST is structured as four key domains. Each domain is judged for risk of bias (low, high or unclear) and includes signalling questions to help make judgements. Signalling questions are rated as yes (Y), probably yes (PY), probably no (PN), no (N) or no information (NI). All signalling questions are phrased so that “yes” indicates absence of bias. Any signalling question rated as “no” or “probably no” flags the potential for bias; you will need to use your judgement to determine whether the domain should be rated as “high”, “low” or “unclear” risk of bias. The guidance document contains further instructions and examples on rating signalling questions and risk of bias for each domain.  The first three domains are also rated for concerns regarding applicability (low/ high/ unclear) to your review question defined above.  *Complete all domains separately for each evaluation of a distinct model. Shaded boxes indicate where signalling questions do not apply and should not be answered.* |
| --- |

| **DOMAIN 1: Participants** | | | |
| --- | --- | --- | --- |
| **A. Risk of Bias** | | | |
| *Describe the sources of data and criteria for participant selection:*  A relevant dataset for the prediction task was obtained from the Psychiatry Department of the University Medical Center Utrecht (UMCU) in The Netherlands. Admissions from all six units between 2013 and 2016 were included in the dataset, resulting in a total of 2521 admissions from 1796 unique patients, including readmissions and transfers between different units  In the relevant time period, a total of 1267 violent incidents were reported. After excluding incidents that did not involve a patient that was admitted at the time of the incident (n = 19), for example incidents that involved visitors rather than the patient or incidents that happened after dismissal of a patient, a total of 1248 incidents remained. | | | |
|  | | Dev | Val |
| - 1. Were appropriate data sources used, e.g. cohort, RCT or nested case-control study data? | | N |  |
| - 1. Were all inclusions and exclusions of participants appropriate? | | Y |  |
| **Risk of bias introduced by selection of participants** | **RISK:**  *(low/ high/ unclear)* | **High** |  |
| *Rationale of bias rating:* | | | |
| 1.1 is N because the design is retrospective, consequently data were collected for other purposes. | | | |
| **B. Applicability** | | | |
| *Describe included participants, setting and dates:*  A relevant dataset for the prediction task was obtained from the Psychiatry Department of the University Medical Center Utrecht (UMCU) in The Netherlands. Admissions from all six units between 2013 and 2016 were included in the dataset, resulting in a total of 2521 admissions from 1796 unique patients, including readmissions and transfers between different units | | | |
| **Concern that the included participants and setting do not match the review question** | **CONCERN:**  *(low/ high/ unclear)* | **Low** |  |
| *Rationale of applicability rating:* | | | |
| Participants and setting seem to fit review question | | | |

| **DOMAIN 2: Predictors** | | | |
| --- | --- | --- | --- |
| **A. Risk of Bias** | | | |
| *List and describe predictors included in the final model, e.g. definition and timing of assessment:*  Much of the clinically relevant information was entered into the EHR in free text format, either by psychiatrists or nurses. These text entries typically contained between 100 and 500 words and were respectively referred to as doctor notes and nurse notes  The complete corpus of doctor and nurse notes (i.e., all notes written before, during or after admission) in the same time period was also made available, totaling 1,015,931 doctor and nurse notes combined | | | |
|  | | Dev | Val |
| - 1. Were predictors defined and assessed in a similar way for all participants? | | PY |  |
| - 1. Were predictor assessments made without knowledge of outcome data? | | Y |  |
| - 1. Are all predictors available at the time the model is intended to be used? | | Y |  |
| **Risk of bias introduced by predictors or their assessment** | **RISK:**  *(low/ high/ unclear)* | **Low** |  |
| *Rationale of bias rating:*  No issues emerged | | | |
| **B. Applicability** | | | |
| Concern that the definition, assessment or timing of predictors in the model do not match the review question | **CONCERN:**  *(low/ high/ unclear)* | **Low** |  |
| *Rationale of applicability rating:*  Included predictors appear representative of the predictors specified in the review question. | | | |

| **DOMAIN 3: Outcome** | | | |
| --- | --- | --- | --- |
| **A. Risk of Bias** | | | |
| *Describe the outcome, how it was defined and determined, and the time interval between predictor assessment and outcome determination:*  **Violent incidents** = incidents concerned violence from patients directed at staff or  at other patients, including both verbal and physical aggression in the first 30 days after admission | | | |
|  | | Dev | Val |
| - 1. Was the outcome determined appropriately? | | Y |  |
| - 1. Was a pre-specified or standard outcome definition used? | | Y |  |
| - 1. Were predictors excluded from the outcome definition? | | Y |  |
| - 1. Was the outcome defined and determined in a similar way for all participants? | | Y |  |
| - 1. Was the outcome determined without knowledge of predictor information? | | PY |  |
| - 1. Was the time interval between predictor assessment and outcome determination appropriate? | | Y |  |
| **Risk of bias introduced by the outcome or its determination** | **RISK:**  *(low/ high/ unclear)* | **Low** |  |
| *Rationale of bias rating:*  No issues emerged | | | |
| **B. Applicability** | | | |
| *At what time point was the outcome determined:*  Outcome (violent incident) could occur in the first 30 days of admission  *If a composite outcome was used, describe the relative frequency/distribution of each contributing outcome:*  N/A | | | |
| **Concern that the outcome, its definition, timing or determination do not match the review question** | **CONCERN:**  *(low/ high/ unclear)* | **Low** |  |
| *Rationale of applicability rating:*  The outcome of the primary study matches the outcome of interest of the review. | | | |

| **DOMAIN 4: Analysis** | | | |
| --- | --- | --- | --- |
| **Risk of Bias** | | | |
| *Describe numbers of participants, number of candidate predictors, outcome events and events per candidate predictor:*  Admissions from all six units between 2013 and 2016 were included in the dataset, resulting in a total of 2521 admissions from 1796 unique patients, including readmissions and transfers between different units.  Predictors: The complete corpus of doctor and nurse notes (i.e., all notes written before, during or after admission) in the same time period was also made available, totaling 1,015,931 doctor and nurse notes combined  In the relevant time period, a total of 1267 violent incidents were reported. | | | |
| *Describe how the model was developed (for example in regards to modelling technique (e.g. survival or logistic modelling), predictor selection, and risk group definition):*  The subset of notes that was available at the start of admission served as input for the prediction problem, while the entire corpus of notes was used to learn representations | | | |
| *Describe whether and how the model was validated, either internally (e.g. bootstrapping, cross validation, random split sample) or externally (e.g. temporal validation, geographical validation, different setting, different type of participants):*  {Bibliography} | | | |
| *Describe the performance measures of the model, e.g. (re)calibration, discrimination, (re)classification, net benefit, and whether they were adjusted for optimism:*  The performance was measured in AUC, along with its standard deviation | | | |
| *Describe any participants who were excluded from the analysis:*  After excluding incidents that did not involve a patient that was admitted at the time of the incident (n = 19), for example incidents that involved visitors rather than the patient or incidents that happened after dismissal of a patient, a total of 1248 incidents remained. | | | |
| *Describe missing data on predictors and outcomes as well as methods used for missing data:*  No information on missing data | | | |
|  | | Dev | Val |
| - 1. Were there a reasonable number of participants with the outcome? | | Y |  |
| - 1. Were continuous and categorical predictors handled appropriately? | | Y |  |
| - 1. Were all enrolled participants included in the analysis? | | Y |  |
| - 1. Were participants with missing data handled appropriately? | | NI |  |
| - 1. Was selection of predictors based on univariable analysis avoided? | | Y |  |
| - 1. Were complexities in the data (e.g. censoring, competing risks, sampling of controls) accounted for appropriately? | | Y |  |
| - 1. Were relevant model performance measures evaluated appropriately? | | Y |  |
| - 1. Were model overfitting and optimism in model performance accounted for? | | Y |  |
| - 1. Do predictors and their assigned weights in the final model correspond to the results from multivariable analysis? | | NI |  |
| **Risk of bias introduced by the analysis** | **RISK:**  *(low/ high/ unclear)* | **Low** |  |
| *Rationale of bias rating:*  No problematic issues emerged | | | |

**Step 4: Overall assessment**

| Use the following tables to reach overall judgements about risk of bias and concerns regarding applicability of the prediction model evaluation (development and/or validation) across all assessed domains.  *Complete for each evaluation of a distinct model.*   \| **Reaching an overall judgement about risk of bias of the prediction model evaluation** \| \| \| --- \| --- \| \| **Low risk of bias** \| If all domains were rated low risk of bias.  If a prediction model was developed without any external validation, and it was rated as low risk of bias for all domains, consider downgrading to **high risk of bias**. Such a model can only be considered as low risk of bias, if the development was based on a very large data set and included some form of internal validation. \| \| **High risk of bias** \| If at least one domain is judged to be at **high risk of bias**. \| \| **Unclear risk of bias** \| If an unclear risk of bias was noted in at least one domain and it was low risk for all other domains. \|  \| **Reaching an overall judgement about applicability of the prediction model evaluation** \| \| \| --- \| --- \| \| **Low concerns regarding applicability** \| If low concerns regarding applicability for all domains, the prediction model evaluation is judged to have **low concerns regarding applicability**. \| \| **High concerns regarding applicability** \| If high concerns regarding applicability for at least one domain, the prediction model evaluation is judged to have **high concerns regarding applicability**. \| \| **Unclear concerns regarding applicability** \| If unclear concerns (but no “high concern”) regarding applicability for at least one domain, the prediction model evaluation is judged to have **unclear concerns regarding applicability** overall. \| |
| --- | --- | --- | --- | --- | --- | --- | --- | --- | --- | --- | --- | --- | --- | --- | --- | --- |

| **Overall judgement about risk of bias and applicability of the prediction model evaluation** | | |
| --- | --- | --- |
| **Overall judgement of risk of bias** | **RISK:**  *(low/ high/ unclear)* | **High** |
| *Summary of sources of potential bias:*  The design is retrospective, consequently data were collected for other purposes. | | |
| **Overall judgement of applicability** | **CONCERN:**  *(low/ high/ unclear)* | **Low** |
| *Summary of applicability concerns:*  No issues emerged | | |

**PROBAST – First evaluator**

(Prediction model study Risk Of Bias Assessment Tool)

Published in Annals of Internal Medicine (freely available):

1. [PROBAST: A Tool to Assess the Risk of Bias and Applicability of Prediction Model Studies](https://annals.org/aim/fullarticle/2719961/probast-tool-assess-risk-bias-applicability-prediction-model-studies)
2. [PROBAST: A Tool to Assess Risk of Bias and Applicability of Prediction Model Studies: Explanation and Elaboration](https://annals.org/aim/fullarticle/2719962/probast-tool-assess-risk-bias-applicability-prediction-model-studies-explanation)

| **What does PROBAST assess?**  PROBAST assesses both the *risk of bias* and *concerns regarding applicability* of a study that evaluates (develops, validates or updates) a multivariable diagnostic or prognostic prediction model. It is designed to assess primary studies included in a systematic review.  *Bias* occurs if systematic flaws or limitations in the design, conduct or analysis of a primary study distort the results. For the purpose of prediction modelling studies, we have defined *risk of bias* to occur when shortcomings in the study design, conduct or analysis lead to systematically distorted estimates of a model’s predictive performance or to an inadequate model to address the research question. Model predictive performance is typically evaluated using calibration, discrimination and sometimes classification measures, and these are likely inaccurately estimated in studies with high risk of bias. *Applicability* refers to the extent to which the prediction model from the primary study matches your systematic review question, for example in terms of the participants, predictors or outcome of interest.  A primary study may include the development and/or validation or update of more than one prediction model. A PROBAST assessment should be completed for each distinct model that is developed, validated or updated (extended) for making individualised predictions. Where a publication assesses multiple prediction models, only complete a PROBAST assessment for those models that meet the inclusion criteria for your systematic review. Please note that subsequent use of the term “model” includes derivatives of models, such as simplified risk scores, nomograms, or recalibrations of models.  PROBAST is not designed for all multivariable diagnostic or prognostic studies. For example, studies using multivariable models to identify predictors associated with an outcome but not attempting to develop a model for making individualised predictions are not covered by PROBAST.  PROBAST includes four steps.   \| **Step** \| **Task** \| **When to complete** \| \| --- \| --- \| --- \| \| **1** \| Specify your systematic review question(s) \| Once per systematic review \| \| **2** \| Classify the type of prediction model evaluation \| Once for each model of interest in each publication being assessed, for each relevant outcome \| \| **3** \| Assess risk of bias and applicability \| Once for each development and validation of each distinct prediction model in a publication \| \| **4** \| Overall judgment \| Once for each development and validation of each distinct prediction model in a publication \|   If this is your first time using PROBAST, we strongly recommend reading the detailed explanation and elaboration (E&E, see link above) paper and to check the examples on www.probast.org |
| --- | --- | --- | --- | --- | --- | --- | --- | --- | --- | --- | --- | --- | --- | --- | --- |

**Step 1: Specify your systematic review question**

| State your systematic review question to facilitate the assessment of the applicability of the evaluated models to your question. *The following table should be completed once per systematic review.* |
| --- |

| **Criteria** | **Specify your systematic review question** |
| --- | --- |
| *Intended use of model:* | ML models for predicting risk of aggression and/or violence |
| ***Participants*** *including selection criteria and setting:* | Psychiatric patients in clinical and forensic settings |
| ***Predictors*** *(used in prediction modelling), including types of predictors (e.g. history, clinical examination, biochemical markers, imaging tests), time of measurement, specific measurement issues (e.g., any requirements/ prohibitions for specialized equipment):* | Clinical, sociodemographic and historical characteristics |
| *Outcome to be predicted:* | Violent episode |

**Step 2: Classify the type of prediction model evaluation**

| Use the following table to classify the evaluation as model development, model validation or model update, or combination. Different signalling questions apply for different types of prediction model evaluation. If the evaluation does not fit one of these classifications then PROBAST should not be used. |
| --- |

| **Classify the evaluation based on its aim** | | | |
| --- | --- | --- | --- |
| **Type of prediction study** | **PROBAST boxes to complete** | **Tick as appropriate** | **Definition for type of prediction model study** |
| Development only | Development | X | Prediction model development without external validation. These studies may include internal validation methods, such as bootstrapping and cross-validation techniques. |
| Development and validation | Development and validation | 🗹 | Prediction model development combined with external validation in other participants in the same article. |
| Validation only | Validation | X | External validation of existing (previously developed) model in other participants. |

| *This table should be completed once for each publication being assessed and for each relevant outcome in your review.* | |  |
| --- | --- | --- |
| **Publication reference** | Menger et al. 2019. Machine Learning Approach to Inpatient Violence Risk Assessment Using Routinely Collected Clinical Notes in Electronic Health Records | |
| **Models of interest** | Support Vector Machine | |
| **Outcome of interest** | Inpatients violent incidents | |

**Step 3: Assess risk of bias and applicability**

| PROBAST is structured as four key domains. Each domain is judged for risk of bias (low, high or unclear) and includes signalling questions to help make judgements. Signalling questions are rated as yes (Y), probably yes (PY), probably no (PN), no (N) or no information (NI). All signalling questions are phrased so that “yes” indicates absence of bias. Any signalling question rated as “no” or “probably no” flags the potential for bias; you will need to use your judgement to determine whether the domain should be rated as “high”, “low” or “unclear” risk of bias. The guidance document contains further instructions and examples on rating signalling questions and risk of bias for each domain.  The first three domains are also rated for concerns regarding applicability (low/ high/ unclear) to your review question defined above.  *Complete all domains separately for each evaluation of a distinct model. Shaded boxes indicate where signalling questions do not apply and should not be answered.* |
| --- |

| **DOMAIN 1: Participants** | | | |
| --- | --- | --- | --- |
| **A. Risk of Bias** | | | |
| *Describe the sources of data and criteria for participant selection:*  Clinical notes recorded during a total of 3189 admissions of 2209 unique individuals at site 1 and 3253 admissions of 1919 unique individuals at site 2 were analyzed.  No exclusion criteria for individual patients were defined | | | |
|  | | Dev | Val |
| - 1. Were appropriate data sources used, e.g. cohort, RCT or nested case-control study data? | | PN | N |
| - 1. Were all inclusions and exclusions of participants appropriate? | | Y | Y |
| **Risk of bias introduced by selection of participants** | **RISK:**  *(low/ high/ unclear)* | **High** | **High** |
| *Rationale of bias rating:* | | | |
| 1.1 is N because the design is retrospective, consequently data were collected for other purposes. | | | |
| **B. Applicability** | | | |
| *Describe included participants, setting and dates:*  Clinical notes recorded during a total of 3189 admissions of 2209 unique individuals at site 1 and 3253 admissions of 1919 unique individuals at site 2 were analyzed.  No exclusion criteria for individual patients were defined | | | |
| **Concern that the included participants and setting do not match the review question** | **CONCERN:**  *(low/ high/ unclear)* | **Low** | **Low** |
| *Rationale of applicability rating:* | | | |
| Participants and setting seem to fit review question | | | |

| **DOMAIN 2: Predictors** | | | |
| --- | --- | --- | --- |
| **A. Risk of Bias** | | | |
| *List and describe predictors included in the final model, e.g. definition and timing of assessment:*  To examine the potential predictive power hidden in clinical notes, the 1000 most frequent terms in the clinical notes, including bigrams, as binary variables were extracted. The authors then assessed the strength of each term’s association with the outcome using a χ2 test and computed Matthews correlation coefficients to obtain the direction of the association. Then they selected the top 10% of predictors on the basis of their χ2 scores in 1000 repeated samples with replacement, computing the fraction of times a term was included among the top predictors as a measure of within–data set generalizability of predictors. | | | |
|  | | Dev | Val |
| - 1. Were predictors defined and assessed in a similar way for all participants? | | Y | Y |
| - 1. Were predictor assessments made without knowledge of outcome data? | | Y | Y |
| - 1. Are all predictors available at the time the model is intended to be used? | | Y | Y |
| **Risk of bias introduced by predictors or their assessment** | **RISK:**  *(low/ high/ unclear)* | **Low** | **Low** |
| *Rationale of bias rating:*  No issues emerged | | | |
| **B. Applicability** | | | |
| Concern that the definition, assessment or timing of predictors in the model do not match the review question | **CONCERN:**  *(low/ high/ unclear)* | **Low** | **Low** |
| *Rationale of applicability rating:*  Included predictors appear representative of the predictors specified in the review question. | | | |

| **DOMAIN 3: Outcome** | | | |
| --- | --- | --- | --- |
| **A. Risk of Bias** | | | |
| *Describe the outcome, how it was defined and determined, and the time interval between predictor assessment and outcome determination:*  Reports of violent incidents were used to determine the outcome for each admission. In both sites mandatory reporting of all violent incidents takes place, including patient-staff and patient-patient violence. On the incident form, staff members who were involved in the incident were required to fill in structured information, a textual description of the incident, and incident severity as measured by the Staff Observation Aggression Scale–Revised. The definition of a violent incident included all threatening and violent behavior of a verbal or physical nature directed at another person  A positive outcome was defined as the presence of at least 1 incident in the first 4 weeks of admission, excluding the first 24 hours. No distinction in incident severity was made | | | |
|  | | Dev | Val |
| - 1. Was the outcome determined appropriately? | | Y | Y |
| - 1. Was a pre-specified or standard outcome definition used? | | Y | Y |
| - 1. Were predictors excluded from the outcome definition? | | PY | PY |
| - 1. Was the outcome defined and determined in a similar way for all participants? | | Y | Y |
| - 1. Was the outcome determined without knowledge of predictor information? | | PY | PY |
| - 1. Was the time interval between predictor assessment and outcome determination appropriate? | | Y | Y |
| **Risk of bias introduced by the outcome or its determination** | **RISK:**  *(low/ high/ unclear)* | **Low** | **Low** |
| *Rationale of bias rating:*  No issues emerged | | | |
| **B. Applicability** | | | |
| *At what time point was the outcome determined:*  First 4 weeks after admission  *If a composite outcome was used, describe the relative frequency/distribution of each contributing outcome:*  N/A | | | |
| **Concern that the outcome, its definition, timing or determination do not match the review question** | **CONCERN:**  *(low/ high/ unclear)* | **Low** | **Low** |
| *Rationale of applicability rating:*  The outcome of the primary study matches the outcome of interest of the review. | | | |

| **DOMAIN 4: Analysis** | | | |
| --- | --- | --- | --- |
| **Risk of Bias** | | | |
| *Describe numbers of participants, number of candidate predictors, outcome events and events per candidate predictor:*  The final data sets consisted of 3189 admissions from 2209 unique patients in site 1 and 3253 admissions from 1919 unique patients in site 2.  Similar admissions with a violent incidence (290 [9.1%] and 247 [7.7%]) were registered in both sites. | | | |
| *Describe how the model was developed (for example in regards to modelling technique (e.g. survival or logistic modelling), predictor selection, and risk group definition):*  The machine learning approach applied to textual data comprised 2 steps: transforming clinical notes into a suitable numerical representation and subsequently feeding these numerical representations into a classification model | | | |
| *Describe whether and how the model was validated, either internally (e.g. bootstrapping, cross validation, random split sample) or externally (e.g. temporal validation, geographical validation, different setting, different type of participants):*  Model training and estimation of model predictive validity were done in a nested cross-validation setup, ensuring that admissions used for learning models were never used to simultaneously determine predictive validity.  After finalizing the results in site 1, an external validation of the machine learning approach was performed in site 2 by training a new model with equal experimental setup. | | | |
| *Describe the performance measures of the model, e.g. (re)calibration, discrimination, (re)classification, net benefit, and whether they were adjusted for optimism:*  AUC, specificity, sensitivity | | | |
| *Describe any participants who were excluded from the analysis:*  Admissions with fewer than 100 words registered after 24 hours (12 admissions in site 1 and 24 admissions in site 2) were excluded from the data set | | | |
| *Describe missing data on predictors and outcomes as well as methods used for missing data:*  No information on missing data | | | |
|  | | Dev | Val |
| - 1. Were there a reasonable number of participants with the outcome? | | Y | Y |
| - 1. Were continuous and categorical predictors handled appropriately? | | Y | Y |
| - 1. Were all enrolled participants included in the analysis? | | Y | Y |
| - 1. Were participants with missing data handled appropriately? | | NI | NI |
| - 1. Was selection of predictors based on univariable analysis avoided? | | Y |  |
| - 1. Were complexities in the data (e.g. censoring, competing risks, sampling of controls) accounted for appropriately? | | Y | Y |
| - 1. Were relevant model performance measures evaluated appropriately? | | Y | Y |
| - 1. Were model overfitting and optimism in model performance accounted for? | | Y |  |
| - 1. Do predictors and their assigned weights in the final model correspond to the results from multivariable analysis? | | NI |  |
| **Risk of bias introduced by the analysis** | **RISK:**  *(low/ high/ unclear)* | **Low** | **low** |
| *Rationale of bias rating:*  No problematic issues emerged | | | |

**Step 4: Overall assessment**

| Use the following tables to reach overall judgements about risk of bias and concerns regarding applicability of the prediction model evaluation (development and/or validation) across all assessed domains.  *Complete for each evaluation of a distinct model.*   \| **Reaching an overall judgement about risk of bias of the prediction model evaluation** \| \| \| --- \| --- \| \| **Low risk of bias** \| If all domains were rated low risk of bias.  If a prediction model was developed without any external validation, and it was rated as low risk of bias for all domains, consider downgrading to **high risk of bias**. Such a model can only be considered as low risk of bias, if the development was based on a very large data set and included some form of internal validation. \| \| **High risk of bias** \| If at least one domain is judged to be at **high risk of bias**. \| \| **Unclear risk of bias** \| If an unclear risk of bias was noted in at least one domain and it was low risk for all other domains. \|  \| **Reaching an overall judgement about applicability of the prediction model evaluation** \| \| \| --- \| --- \| \| **Low concerns regarding applicability** \| If low concerns regarding applicability for all domains, the prediction model evaluation is judged to have **low concerns regarding applicability**. \| \| **High concerns regarding applicability** \| If high concerns regarding applicability for at least one domain, the prediction model evaluation is judged to have **high concerns regarding applicability**. \| \| **Unclear concerns regarding applicability** \| If unclear concerns (but no “high concern”) regarding applicability for at least one domain, the prediction model evaluation is judged to have **unclear concerns regarding applicability** overall. \| |
| --- | --- | --- | --- | --- | --- | --- | --- | --- | --- | --- | --- | --- | --- | --- | --- | --- |

| **Overall judgement about risk of bias and applicability of the prediction model evaluation** | | |
| --- | --- | --- |
| **Overall judgement of risk of bias** | **RISK:**  *(low/ high/ unclear)* | **High** |
| *Summary of sources of potential bias:*  The design is retrospective, consequently data were collected for other purposes. | | |
| **Overall judgement of applicability** | **CONCERN:**  *(low/ high/ unclear)* | **Low** |
| *Summary of applicability concerns:*  No issues emerged | | |

**PROBAST – First evaluator**

(Prediction model study Risk Of Bias Assessment Tool)

Published in Annals of Internal Medicine (freely available):

1. [PROBAST: A Tool to Assess the Risk of Bias and Applicability of Prediction Model Studies](https://annals.org/aim/fullarticle/2719961/probast-tool-assess-risk-bias-applicability-prediction-model-studies)
2. [PROBAST: A Tool to Assess Risk of Bias and Applicability of Prediction Model Studies: Explanation and Elaboration](https://annals.org/aim/fullarticle/2719962/probast-tool-assess-risk-bias-applicability-prediction-model-studies-explanation)

| **What does PROBAST assess?**  PROBAST assesses both the *risk of bias* and *concerns regarding applicability* of a study that evaluates (develops, validates or updates) a multivariable diagnostic or prognostic prediction model. It is designed to assess primary studies included in a systematic review.  *Bias* occurs if systematic flaws or limitations in the design, conduct or analysis of a primary study distort the results. For the purpose of prediction modelling studies, we have defined *risk of bias* to occur when shortcomings in the study design, conduct or analysis lead to systematically distorted estimates of a model’s predictive performance or to an inadequate model to address the research question. Model predictive performance is typically evaluated using calibration, discrimination and sometimes classification measures, and these are likely inaccurately estimated in studies with high risk of bias. *Applicability* refers to the extent to which the prediction model from the primary study matches your systematic review question, for example in terms of the participants, predictors or outcome of interest.  A primary study may include the development and/or validation or update of more than one prediction model. A PROBAST assessment should be completed for each distinct model that is developed, validated or updated (extended) for making individualised predictions. Where a publication assesses multiple prediction models, only complete a PROBAST assessment for those models that meet the inclusion criteria for your systematic review. Please note that subsequent use of the term “model” includes derivatives of models, such as simplified risk scores, nomograms, or recalibrations of models.  PROBAST is not designed for all multivariable diagnostic or prognostic studies. For example, studies using multivariable models to identify predictors associated with an outcome but not attempting to develop a model for making individualised predictions are not covered by PROBAST.  PROBAST includes four steps.   \| **Step** \| **Task** \| **When to complete** \| \| --- \| --- \| --- \| \| **1** \| Specify your systematic review question(s) \| Once per systematic review \| \| **2** \| Classify the type of prediction model evaluation \| Once for each model of interest in each publication being assessed, for each relevant outcome \| \| **3** \| Assess risk of bias and applicability \| Once for each development and validation of each distinct prediction model in a publication \| \| **4** \| Overall judgment \| Once for each development and validation of each distinct prediction model in a publication \|   If this is your first time using PROBAST, we strongly recommend reading the detailed explanation and elaboration (E&E, see link above) paper and to check the examples on www.probast.org |
| --- | --- | --- | --- | --- | --- | --- | --- | --- | --- | --- | --- | --- | --- | --- | --- |

**Step 1: Specify your systematic review question**

| State your systematic review question to facilitate the assessment of the applicability of the evaluated models to your question. *The following table should be completed once per systematic review.* |
| --- |

| **Criteria** | **Specify your systematic review question** |
| --- | --- |
| *Intended use of model:* | ML models for predicting risk of aggression and/or violence |
| ***Participants*** *including selection criteria and setting:* | Psychiatric patients in clinical and forensic settings |
| ***Predictors*** *(used in prediction modelling), including types of predictors (e.g. history, clinical examination, biochemical markers, imaging tests), time of measurement, specific measurement issues (e.g., any requirements/ prohibitions for specialized equipment):* | Clinical, sociodemographic and historical characteristics |
| *Outcome to be predicted:* | Violent episode |

**Step 2: Classify the type of prediction model evaluation**

| Use the following table to classify the evaluation as model development, model validation or model update, or combination. Different signalling questions apply for different types of prediction model evaluation. If the evaluation does not fit one of these classifications then PROBAST should not be used. |
| --- |

| **Classify the evaluation based on its aim** | | | |
| --- | --- | --- | --- |
| **Type of prediction study** | **PROBAST boxes to complete** | **Tick as appropriate** | **Definition for type of prediction model study** |
| Development only | Development | 🗹 | Prediction model development without external validation. These studies may include internal validation methods, such as bootstrapping and cross-validation techniques. |
| Development and validation | Development and validation | X | Prediction model development combined with external validation in other participants in the same article. |
| Validation only | Validation | X | External validation of existing (previously developed) model in other participants. |

| *This table should be completed once for each publication being assessed and for each relevant outcome in your review.* | |  |
| --- | --- | --- |
| **Publication reference** | Suchting et al. 2018. A data science approach to predicting patient aggressive events in a psychiatric hospital | |
| **Models of interest** | penalized Generalized Linear Modeling, Random Forest, Gradient Boosting Machine, Deep Neural Networks | |
| **Outcome of interest** | aggressive event toward staff or patients | |

**Step 3: Assess risk of bias and applicability**

| PROBAST is structured as four key domains. Each domain is judged for risk of bias (low, high or unclear) and includes signalling questions to help make judgements. Signalling questions are rated as yes (Y), probably yes (PY), probably no (PN), no (N) or no information (NI). All signalling questions are phrased so that “yes” indicates absence of bias. Any signalling question rated as “no” or “probably no” flags the potential for bias; you will need to use your judgement to determine whether the domain should be rated as “high”, “low” or “unclear” risk of bias. The guidance document contains further instructions and examples on rating signalling questions and risk of bias for each domain.  The first three domains are also rated for concerns regarding applicability (low/ high/ unclear) to your review question defined above.  *Complete all domains separately for each evaluation of a distinct model. Shaded boxes indicate where signalling questions do not apply and should not be answered.* |
| --- |

| **DOMAIN 1: Participants** | | | |
| --- | --- | --- | --- |
| **A. Risk of Bias** | | | |
| *Describe the sources of data and criteria for participant selection:*  Electronic health records(N=29,841) collected between January 2010 and December 2015 at Harris County Psychiatric Center.  Data were not differentially included or excluded based on any specific diagnostic or admission criteria. | | | |
|  | | Dev | Val |
| - 1. Were appropriate data sources used, e.g. cohort, RCT or nested case-control study data? | | N |  |
| - 1. Were all inclusions and exclusions of participants appropriate? | | Y |  |
| **Risk of bias introduced by selection of participants** | **RISK:**  *(low/ high/ unclear)* | **High** |  |
| *Rationale of bias rating:*  1.1 is N because the design is retrospective, consequently data were collected for other purposes. | | | |
| **B. Applicability** | | | |
| *Describe included participants, setting and dates:*  Electronic health records(N=29,841) collected between January 2010 and December 2015 at Harris County Psychiatric Center | | | |
| **Concern that the included participants and setting do not match the review question** | **CONCERN:**  *(low/ high/ unclear)* | **Low** |  |
| *Rationale of applicability rating:* | | | |
| Participants and setting seem to fit review question | | | |

| **DOMAIN 2: Predictors** | | | |
| --- | --- | --- | --- |
| **A. Risk of Bias** | | | |
| *List and describe predictors included in the final model, e.g. definition and timing of assessment:*  328 predictor variables among which full demographic profile, patient vitals (i.e., height, weight, blood pressure), a comprehensive psychosocial assessment, including histories of early development, education, military service, vocation/work, medical status, psychiatric status, drug/substance use and treatment, nicotine/tobacco use and counseling, abuse (victim or perpetrated physical/verbal/emotional/ sexual abuse), legal status, marital status, religious beliefs, financial status, and living situation. Sleep habits, pain status, patient behavior during interview, a risk assessment, and evaluation of patient mood (via the Affective Disorders Rating Scale. General appearance (i.e., hygiene), musculoskeletal system, speech pattern, thought processes and content, perception, depression, affect, insight, judgment, skin integrity, head trauma, suicidal/homicidal/assault ideation, deterioration in function, chemical dependency, hallucinations, and delusions. | | | |
|  | | Dev | Val |
| - 1. Were predictors defined and assessed in a similar way for all participants? | | Y |  |
| - 1. Were predictor assessments made without knowledge of outcome data? | | PY |  |
| - 1. Are all predictors available at the time the model is intended to be used? | | Y |  |
| **Risk of bias introduced by predictors or their assessment** | **RISK:**  *(low/ high/ unclear)* | **Low** |  |
| *Rationale of bias rating:*  No issues emerged | | | |
| **B. Applicability** | | | |
| Concern that the definition, assessment or timing of predictors in the model do not match the review question | **CONCERN:**  *(low/ high/ unclear)* | **Low** |  |
| *Rationale of applicability rating:*  Included predictors appear representative of the predictors specified in the review question. | | | |

| **DOMAIN 3: Outcome** | | | |
| --- | --- | --- | --- |
| **A. Risk of Bias** | | | |
| *Describe the outcome, how it was defined and determined, and the time interval between predictor assessment and outcome determination:*  Outcome measure = positive/negative for an aggressive incident. An aggressive event is coded into the hospital medical record following any episode of uncontrolled verbal or physical aggression that required intervention by and assistance from additional hospital staff to manage the event | | | |
|  | | Dev | Val |
| - 1. Was the outcome determined appropriately? | | Y |  |
| - 1. Was a pre-specified or standard outcome definition used? | | Y |  |
| - 1. Were predictors excluded from the outcome definition? | | Y |  |
| - 1. Was the outcome defined and determined in a similar way for all participants? | | Y |  |
| - 1. Was the outcome determined without knowledge of predictor information? | | PY |  |
| - 1. Was the time interval between predictor assessment and outcome determination appropriate? | | Y |  |
| **Risk of bias introduced by the outcome or its determination** | **RISK:**  *(low/ high/ unclear)* | **Low** |  |
| *Rationale of bias rating:*  No issues emerged | | | |
| **B. Applicability** | | | |
| *At what time point was the outcome determined:*  No information  *If a composite outcome was used, describe the relative frequency/distribution of each contributing outcome:*  N/A | | | |
| **Concern that the outcome, its definition, timing or determination do not match the review question** | **CONCERN:**  *(low/ high/ unclear)* | **Low** |  |
| *Rationale of applicability rating:*  The outcome of the primary study matches the outcome of interest of the review. | | | |

| **DOMAIN 4: Analysis** | | | |
| --- | --- | --- | --- |
| **Risk of Bias** | | | |
| *Describe numbers of participants, number of candidate predictors, outcome events and events per candidate predictor:*  This retrospective study utilized electronic health records (N=29,841) collected between January 2010 and December 2015 at Harris County Psychiatric Center  328 predictor variables  In the dataset of 29,841 patients, 424 (1.4%) had an identified aggressive incident during the inpatient stay and 29,417 (98.6%) did not. | | | |
| *Describe how the model was developed (for example in regards to modelling technique (e.g. survival or logistic modelling), predictor selection, and risk group definition):*  Four machine learning algorithms were utilized to provide probabilities of an aggressive incident during the inpatient stay: penalized generalized linear modeling (GLM), random forest (RF), gradient boosting machine (GBM), and deep neural network (DNN). | | | |
| *Describe whether and how the model was validated, either internally (e.g. bootstrapping, cross validation, random split sample) or externally (e.g. temporal validation, geographical validation, different setting, different type of participants):*  5-fold cross validation | | | |
| *Describe the performance measures of the model, e.g. (re)calibration, discrimination, (re)classification, net benefit, and whether they were adjusted for optimism:*  Overall classification success was measured using area under the receiver operating characteristic curve (AUC) | | | |
| *Describe any participants who were excluded from the analysis:*  No esclusion | | | |
| *Describe missing data on predictors and outcomes as well as methods used for missing data:*  Data were missing in approximately 10% of observations, with the majority of missingness occurring in the categorical predictors. Assuming that missingness is itself a potentially important factor, missingness in each categorical predictor was addressed through the creation of a new categorical level to indicate “missing.” Remaining missing data (less than 1%) in the continuous predictors were handled natively by each machine learning algorithm (e.g., imputation, data partitioning). | | | |
|  | | Dev | Val |
| - 1. Were there a reasonable number of participants with the outcome? | | Y |  |
| - 1. Were continuous and categorical predictors handled appropriately? | | Y |  |
| - 1. Were all enrolled participants included in the analysis? | | Y |  |
| - 1. Were participants with missing data handled appropriately? | | Y |  |
| - 1. Was selection of predictors based on univariable analysis avoided? | | Y |  |
| - 1. Were complexities in the data (e.g. censoring, competing risks, sampling of controls) accounted for appropriately? | | Y |  |
| - 1. Were relevant model performance measures evaluated appropriately? | | Y |  |
| - 1. Were model overfitting and optimism in model performance accounted for? | | NI |  |
| - 1. Do predictors and their assigned weights in the final model correspond to the results from multivariable analysis? | | NI |  |
| **Risk of bias introduced by the analysis** | **RISK:**  *(low/ high/ unclear)* | **Low** |  |
| *Rationale of bias rating:*  No issues emerged | | | |

**Step 4: Overall assessment**

| Use the following tables to reach overall judgements about risk of bias and concerns regarding applicability of the prediction model evaluation (development and/or validation) across all assessed domains.  *Complete for each evaluation of a distinct model.*   \| **Reaching an overall judgement about risk of bias of the prediction model evaluation** \| \| \| --- \| --- \| \| **Low risk of bias** \| If all domains were rated low risk of bias.  If a prediction model was developed without any external validation, and it was rated as low risk of bias for all domains, consider downgrading to **high risk of bias**. Such a model can only be considered as low risk of bias, if the development was based on a very large data set and included some form of internal validation. \| \| **High risk of bias** \| If at least one domain is judged to be at **high risk of bias**. \| \| **Unclear risk of bias** \| If an unclear risk of bias was noted in at least one domain and it was low risk for all other domains. \|  \| **Reaching an overall judgement about applicability of the prediction model evaluation** \| \| \| --- \| --- \| \| **Low concerns regarding applicability** \| If low concerns regarding applicability for all domains, the prediction model evaluation is judged to have **low concerns regarding applicability**. \| \| **High concerns regarding applicability** \| If high concerns regarding applicability for at least one domain, the prediction model evaluation is judged to have **high concerns regarding applicability**. \| \| **Unclear concerns regarding applicability** \| If unclear concerns (but no “high concern”) regarding applicability for at least one domain, the prediction model evaluation is judged to have **unclear concerns regarding applicability** overall. \| |
| --- | --- | --- | --- | --- | --- | --- | --- | --- | --- | --- | --- | --- | --- | --- | --- | --- |

| **Overall judgement about risk of bias and applicability of the prediction model evaluation** | | |
| --- | --- | --- |
| **Overall judgement of risk of bias** | **RISK:**  *(low/ high/ unclear)* | **high** |
| *Summary of sources of potential bias:*  The design is retrospective, consequently data were collected for other purposes. | | |
| **Overall judgement of applicability** | **CONCERN:**  *(low/ high/ unclear)* | **low** |
| *Summary of applicability concerns:*  No issues emerged | | |

**PROBAST – First evaluator**

(Prediction model study Risk Of Bias Assessment Tool)

Published in Annals of Internal Medicine (freely available):

1. [PROBAST: A Tool to Assess the Risk of Bias and Applicability of Prediction Model Studies](https://annals.org/aim/fullarticle/2719961/probast-tool-assess-risk-bias-applicability-prediction-model-studies)
2. [PROBAST: A Tool to Assess Risk of Bias and Applicability of Prediction Model Studies: Explanation and Elaboration](https://annals.org/aim/fullarticle/2719962/probast-tool-assess-risk-bias-applicability-prediction-model-studies-explanation)

| **What does PROBAST assess?**  PROBAST assesses both the *risk of bias* and *concerns regarding applicability* of a study that evaluates (develops, validates or updates) a multivariable diagnostic or prognostic prediction model. It is designed to assess primary studies included in a systematic review.  *Bias* occurs if systematic flaws or limitations in the design, conduct or analysis of a primary study distort the results. For the purpose of prediction modelling studies, we have defined *risk of bias* to occur when shortcomings in the study design, conduct or analysis lead to systematically distorted estimates of a model’s predictive performance or to an inadequate model to address the research question. Model predictive performance is typically evaluated using calibration, discrimination and sometimes classification measures, and these are likely inaccurately estimated in studies with high risk of bias. *Applicability* refers to the extent to which the prediction model from the primary study matches your systematic review question, for example in terms of the participants, predictors or outcome of interest.  A primary study may include the development and/or validation or update of more than one prediction model. A PROBAST assessment should be completed for each distinct model that is developed, validated or updated (extended) for making individualised predictions. Where a publication assesses multiple prediction models, only complete a PROBAST assessment for those models that meet the inclusion criteria for your systematic review. Please note that subsequent use of the term “model” includes derivatives of models, such as simplified risk scores, nomograms, or recalibrations of models.  PROBAST is not designed for all multivariable diagnostic or prognostic studies. For example, studies using multivariable models to identify predictors associated with an outcome but not attempting to develop a model for making individualised predictions are not covered by PROBAST.  PROBAST includes four steps.   \| **Step** \| **Task** \| **When to complete** \| \| --- \| --- \| --- \| \| **1** \| Specify your systematic review question(s) \| Once per systematic review \| \| **2** \| Classify the type of prediction model evaluation \| Once for each model of interest in each publication being assessed, for each relevant outcome \| \| **3** \| Assess risk of bias and applicability \| Once for each development and validation of each distinct prediction model in a publication \| \| **4** \| Overall judgment \| Once for each development and validation of each distinct prediction model in a publication \|   If this is your first time using PROBAST, we strongly recommend reading the detailed explanation and elaboration (E&E, see link above) paper and to check the examples on www.probast.org |
| --- | --- | --- | --- | --- | --- | --- | --- | --- | --- | --- | --- | --- | --- | --- | --- |

**Step 1: Specify your systematic review question**

| State your systematic review question to facilitate the assessment of the applicability of the evaluated models to your question. *The following table should be completed once per systematic review.* |
| --- |

| **Criteria** | **Specify your systematic review question** |
| --- | --- |
| *Intended use of model:* | ML models for predicting risk of aggression and/or violence |
| ***Participants*** *including selection criteria and setting:* | Psychiatric patients in clinical and forensic settings |
| ***Predictors*** *(used in prediction modelling), including types of predictors (e.g. history, clinical examination, biochemical markers, imaging tests), time of measurement, specific measurement issues (e.g., any requirements/ prohibitions for specialized equipment):* | Clinical, sociodemographic and historical characteristics |
| *Outcome to be predicted:* | Violent episode |

**Step 2: Classify the type of prediction model evaluation**

| Use the following table to classify the evaluation as model development, model validation or model update, or combination. Different signalling questions apply for different types of prediction model evaluation. If the evaluation does not fit one of these classifications then PROBAST should not be used. |
| --- |

| **Classify the evaluation based on its aim** | | | |
| --- | --- | --- | --- |
| **Type of prediction study** | **PROBAST boxes to complete** | **Tick as appropriate** | **Definition for type of prediction model study** |
| Development only | Development | 🗹 | Prediction model development without external validation. These studies may include internal validation methods, such as bootstrapping and cross-validation techniques. |
| Development and validation | Development and validation | X | Prediction model development combined with external validation in other participants in the same article. |
| Validation only | Validation | X | External validation of existing (previously developed) model in other participants. |

| *This table should be completed once for each publication being assessed and for each relevant outcome in your review.* | |  |
| --- | --- | --- |
| **Publication reference** | Wang 2020. Prediction of physical violence in schizophrenia with machine learning algorithms | |
| **Models of interest** | LASSO, elastic net, Random Forest, GBRT, Support Vector Machine, Support Vector Machine with Radial Basis Function kernels | |
| **Outcome of interest** | Prediction of physical violence | |

**Step 3: Assess risk of bias and applicability**

| PROBAST is structured as four key domains. Each domain is judged for risk of bias (low, high or unclear) and includes signalling questions to help make judgements. Signalling questions are rated as yes (Y), probably yes (PY), probably no (PN), no (N) or no information (NI). All signalling questions are phrased so that “yes” indicates absence of bias. Any signalling question rated as “no” or “probably no” flags the potential for bias; you will need to use your judgement to determine whether the domain should be rated as “high”, “low” or “unclear” risk of bias. The guidance document contains further instructions and examples on rating signalling questions and risk of bias for each domain.  The first three domains are also rated for concerns regarding applicability (low/ high/ unclear) to your review question defined above.  *Complete all domains separately for each evaluation of a distinct model. Shaded boxes indicate where signalling questions do not apply and should not be answered.* |
| --- |

| **DOMAIN 1: Participants** | | | |
| --- | --- | --- | --- |
| **A. Risk of Bias** | | | |
| *Describe the sources of data and criteria for participant selection:*  A total of 275 participants with a diagnosis of schizophrenia spectrum disorder were recruited from the Centre for Addiction and Mental Health (CAMH), located in Toronto, Canada  Inclusion criteria of being between the ages of 18 and 75; and the diagnosis of schizophrenia spectrum disorder was confirmed by the Structured Clinical Interview for the DSM-IV (SCID-I/P). Exclusion criteria included having any diagnosis of an intellectual disability, major neurological disorder, or substance- induced psychosis; or having a history of head trauma resulting in loss of consciousness. | | | |
|  | | Dev | Val |
| - 1. Were appropriate data sources used, e.g. cohort, RCT or nested case-control study data? | | PY |  |
| - 1. Were all inclusions and exclusions of participants appropriate? | | Y |  |
| **Risk of bias introduced by selection of participants** | **RISK:**  *(low/ high/ unclear)* | **Low** |  |
| *Rationale of bias rating:* | | | |
| No issue emerged | | | |
| **B. Applicability** | | | |
| *Describe included participants, setting and dates:* | | | |
| **Concern that the included participants and setting do not match the review question** | **CONCERN:**  *(low/ high/ unclear)* | **High** |  |
| *Rationale of applicability rating:* | | | |
| Included patients appear not fully representative of the population specified in the review question (general psychiatric and forensic patients, and not only affected by schizophrenia spectrum disorders. | | | |

| **DOMAIN 2: Predictors** | | | |
| --- | --- | --- | --- |
| **A. Risk of Bias** | | | |
| *List and describe predictors included in the final model, e.g. definition and timing of assessment:*  28 predictive variables based on previously identified factors associated with violence or schizophrenia.  Among them were: age, sex, age of onset of psychosis, number of previous psychiatric hospitalizations, comorbid diagnoses of lifetime alcohol, drug, and marijuana abuse or dependence, family histories of psychosis, mood disorders, suicide, ethnicity, primary language, religious identity, age of immigration, childhood trauma and five-factor personality traits from the NEO Five Factor Inventory (NEO-FFI) | | | |
|  | | Dev | Val |
| - 1. Were predictors defined and assessed in a similar way for all participants? | | Y |  |
| - 1. Were predictor assessments made without knowledge of outcome data? | | PY |  |
| - 1. Are all predictors available at the time the model is intended to be used? | | Y |  |
| **Risk of bias introduced by predictors or their assessment** | **RISK:**  *(low/ high/ unclear)* | **Low** |  |
| *Rationale of bias rating:*  No issues emerged | | | |
| **B. Applicability** | | | |
| Concern that the definition, assessment or timing of predictors in the model do not match the review question | **CONCERN:**  *(low/ high/ unclear)* | **Low** |  |
| *Rationale of applicability rating:*  Included predictors appear representative of the predictors specified in the review question. | | | |

| **DOMAIN 3: Outcome** | | | |
| --- | --- | --- | --- |
| **A. Risk of Bias** | | | |
| *Describe the outcome, how it was defined and determined, and the time interval between predictor assessment and outcome determination:*  The presence of violence was determined retrospectively through a review of all available patient electronic medical records for any documentation of incidences of physically violent behavior. We assigned severity scores for each participant ranging from an absence of physical violence to assault resulting in serious bodily injury, as outlined in the Modified Overt Aggression Scale (MOAS). For the purpose of this study, participants were simply classified as violent or non-violent. | | | |
|  | | Dev | Val |
| - 1. Was the outcome determined appropriately? | | NI |  |
| - 1. Was a pre-specified or standard outcome definition used? | | NI |  |
| - 1. Were predictors excluded from the outcome definition? | | NI |  |
| - 1. Was the outcome defined and determined in a similar way for all participants? | | Y |  |
| - 1. Was the outcome determined without knowledge of predictor information? | | N |  |
| - 1. Was the time interval between predictor assessment and outcome determination appropriate? | | N |  |
| **Risk of bias introduced by the outcome or its determination** | **RISK:**  *(low/ high/ unclear)* | **unclear** |  |
| *Rationale of bias rating:*  It is not clearly defined how the patients were divided in violent/non violent | | | |
| **B. Applicability** | | | |
| *At what time point was the outcome determined:*  Not clearly specified  *If a composite outcome was used, describe the relative frequency/distribution of each contributing outcome:*  N/A | | | |
| **Concern that the outcome, its definition, timing or determination do not match the review question** | **CONCERN:**  *(low/ high/ unclear)* | **Low** |  |
| *Rationale of applicability rating:*  The outcome of the primary study matches the outcome of interest of the review. | | | |

| **DOMAIN 4: Analysis** | | | |
| --- | --- | --- | --- |
| **Risk of Bias** | | | |
| *Describe numbers of participants, number of candidate predictors, outcome events and events per candidate predictor:*  A total of 275 participants with a diagnosis of schizophrenia spectrum disorder were recruited from the Centre for Addiction and Mental Health (CAMH), located in Toronto, Canada  28 predictors  103 patients were classified as violent | | | |
| *Describe how the model was developed (for example in regards to modelling technique (e.g. survival or logistic modelling), predictor selection, and risk group definition):*  Six classification algorithms were performed for model prediction comparison: the least absolute shrinkage and selection operator (lasso), elastic net, random forest, gradient boosted regression trees (GBRT), support vector machine (SVM) classifier, and SVM classifiers with radial basis function (RBF) kernels. | | | |
| *Describe whether and how the model was validated, either internally (e.g. bootstrapping, cross validation, random split sample) or externally (e.g. temporal validation, geographical validation, different setting, different type of participants):*  5-fold cross validation technique. | | | |
| *Describe the performance measures of the model, e.g. (re)calibration, discrimination, (re)classification, net benefit, and whether they were adjusted for optimism:*  Measures of algorithm performance include the overall predictive accuracy, area under the receiver operator characteristic (ROC) curve (AUROC), sensitivity, specificity, positive predictive value (PPV), and negative predictive value (NPV). | | | |
| *Describe any participants who were excluded from the analysis:*  No information | | | |
| *Describe missing data on predictors and outcomes as well as methods used for missing data:*  No information | | | |
|  | | Dev | Val |
| - 1. Were there a reasonable number of participants with the outcome? | | Y |  |
| - 1. Were continuous and categorical predictors handled appropriately? | | PY |  |
| - 1. Were all enrolled participants included in the analysis? | | Y |  |
| - 1. Were participants with missing data handled appropriately? | | NI |  |
| - 1. Was selection of predictors based on univariable analysis avoided? | | Y |  |
| - 1. Were complexities in the data (e.g. censoring, competing risks, sampling of controls) accounted for appropriately? | | NI |  |
| - 1. Were relevant model performance measures evaluated appropriately? | | Y |  |
| - 1. Were model overfitting and optimism in model performance accounted for? | | Y |  |
| - 1. Do predictors and their assigned weights in the final model correspond to the results from multivariable analysis? | | NI |  |
| **Risk of bias introduced by the analysis** | **RISK:**  *(low/ high/ unclear)* | **Unclear** |  |
| *Rationale of bias rating:*  No information on missing data | | | |

**Step 4: Overall assessment**

| Use the following tables to reach overall judgements about risk of bias and concerns regarding applicability of the prediction model evaluation (development and/or validation) across all assessed domains.  *Complete for each evaluation of a distinct model.*   \| **Reaching an overall judgement about risk of bias of the prediction model evaluation** \| \| \| --- \| --- \| \| **Low risk of bias** \| If all domains were rated low risk of bias.  If a prediction model was developed without any external validation, and it was rated as low risk of bias for all domains, consider downgrading to **high risk of bias**. Such a model can only be considered as low risk of bias, if the development was based on a very large data set and included some form of internal validation. \| \| **High risk of bias** \| If at least one domain is judged to be at **high risk of bias**. \| \| **Unclear risk of bias** \| If an unclear risk of bias was noted in at least one domain and it was low risk for all other domains. \|  \| **Reaching an overall judgement about applicability of the prediction model evaluation** \| \| \| --- \| --- \| \| **Low concerns regarding applicability** \| If low concerns regarding applicability for all domains, the prediction model evaluation is judged to have **low concerns regarding applicability**. \| \| **High concerns regarding applicability** \| If high concerns regarding applicability for at least one domain, the prediction model evaluation is judged to have **high concerns regarding applicability**. \| \| **Unclear concerns regarding applicability** \| If unclear concerns (but no “high concern”) regarding applicability for at least one domain, the prediction model evaluation is judged to have **unclear concerns regarding applicability** overall. \| |
| --- | --- | --- | --- | --- | --- | --- | --- | --- | --- | --- | --- | --- | --- | --- | --- | --- |

| **Overall judgement about risk of bias and applicability of the prediction model evaluation** | | |
| --- | --- | --- |
| **Overall judgement of risk of bias** | **RISK:**  *(low/ high/ unclear)* | **High** |
| *Summary of sources of potential bias:*  It is not clearly specified how they divided patients in volent/non violent | | |
| **Overall judgement of applicability** | **CONCERN:**  *(low/ high/ unclear)* | **High** |
| *Summary of applicability concerns:*  Included patients appear not fully representative of the population specified in the review question (general psychiatric and forensic patients, and not only affected by schizophrenia spectrum disorders. | | |

**PROBAST – First evaluator**

(Prediction model study Risk Of Bias Assessment Tool)

Published in Annals of Internal Medicine (freely available):

1. [PROBAST: A Tool to Assess the Risk of Bias and Applicability of Prediction Model Studies](https://annals.org/aim/fullarticle/2719961/probast-tool-assess-risk-bias-applicability-prediction-model-studies)
2. [PROBAST: A Tool to Assess Risk of Bias and Applicability of Prediction Model Studies: Explanation and Elaboration](https://annals.org/aim/fullarticle/2719962/probast-tool-assess-risk-bias-applicability-prediction-model-studies-explanation)

| **What does PROBAST assess?**  PROBAST assesses both the *risk of bias* and *concerns regarding applicability* of a study that evaluates (develops, validates or updates) a multivariable diagnostic or prognostic prediction model. It is designed to assess primary studies included in a systematic review.  *Bias* occurs if systematic flaws or limitations in the design, conduct or analysis of a primary study distort the results. For the purpose of prediction modelling studies, we have defined *risk of bias* to occur when shortcomings in the study design, conduct or analysis lead to systematically distorted estimates of a model’s predictive performance or to an inadequate model to address the research question. Model predictive performance is typically evaluated using calibration, discrimination and sometimes classification measures, and these are likely inaccurately estimated in studies with high risk of bias. *Applicability* refers to the extent to which the prediction model from the primary study matches your systematic review question, for example in terms of the participants, predictors or outcome of interest.  A primary study may include the development and/or validation or update of more than one prediction model. A PROBAST assessment should be completed for each distinct model that is developed, validated or updated (extended) for making individualised predictions. Where a publication assesses multiple prediction models, only complete a PROBAST assessment for those models that meet the inclusion criteria for your systematic review. Please note that subsequent use of the term “model” includes derivatives of models, such as simplified risk scores, nomograms, or recalibrations of models.  PROBAST is not designed for all multivariable diagnostic or prognostic studies. For example, studies using multivariable models to identify predictors associated with an outcome but not attempting to develop a model for making individualised predictions are not covered by PROBAST.  PROBAST includes four steps.   \| **Step** \| **Task** \| **When to complete** \| \| --- \| --- \| --- \| \| **1** \| Specify your systematic review question(s) \| Once per systematic review \| \| **2** \| Classify the type of prediction model evaluation \| Once for each model of interest in each publication being assessed, for each relevant outcome \| \| **3** \| Assess risk of bias and applicability \| Once for each development and validation of each distinct prediction model in a publication \| \| **4** \| Overall judgment \| Once for each development and validation of each distinct prediction model in a publication \|   If this is your first time using PROBAST, we strongly recommend reading the detailed explanation and elaboration (E&E, see link above) paper and to check the examples on www.probast.org |
| --- | --- | --- | --- | --- | --- | --- | --- | --- | --- | --- | --- | --- | --- | --- | --- |

**Step 1: Specify your systematic review question**

| State your systematic review question to facilitate the assessment of the applicability of the evaluated models to your question. *The following table should be completed once per systematic review.* |
| --- |

| **Criteria** | **Specify your systematic review question** |
| --- | --- |
| *Intended use of model:* | ML models for predicting risk of aggression and/or violence |
| ***Participants*** *including selection criteria and setting:* | Psychiatric patients in clinical and forensic settings |
| ***Predictors*** *(used in prediction modelling), including types of predictors (e.g. history, clinical examination, biochemical markers, imaging tests), time of measurement, specific measurement issues (e.g., any requirements/ prohibitions for specialized equipment):* | Clinical, sociodemographic and historical characteristics |
| *Outcome to be predicted:* | Violent episode |

**Step 2: Classify the type of prediction model evaluation**

| Use the following table to classify the evaluation as model development, model validation or model update, or combination. Different signalling questions apply for different types of prediction model evaluation. If the evaluation does not fit one of these classifications then PROBAST should not be used. |
| --- |

| **Classify the evaluation based on its aim** | | | |
| --- | --- | --- | --- |
| **Type of prediction study** | **PROBAST boxes to complete** | **Tick as appropriate** | **Definition for type of prediction model study** |
| Development only | Development | 🗹 | Prediction model development without external validation. These studies may include internal validation methods, such as bootstrapping and cross-validation techniques. |
| Development and validation | Development and validation | X | Prediction model development combined with external validation in other participants in the same article. |
| Validation only | Validation | X | External validation of existing (previously developed) model in other participants. |

| *This table should be completed once for each publication being assessed and for each relevant outcome in your review.* | |  |
| --- | --- | --- |
| **Publication reference** | Watts 2021. Predicting offenses among individuals with psychiatric disorders - A machine learning approach | |
| **Models of interest** | Machine learning algorithms (Random Forest, Elastic Net, SVM) | |
| **Outcome of interest** | Predicting offenses | |

**Step 3: Assess risk of bias and applicability**

| PROBAST is structured as four key domains. Each domain is judged for risk of bias (low, high or unclear) and includes signalling questions to help make judgements. Signalling questions are rated as yes (Y), probably yes (PY), probably no (PN), no (N) or no information (NI). All signalling questions are phrased so that “yes” indicates absence of bias. Any signalling question rated as “no” or “probably no” flags the potential for bias; you will need to use your judgement to determine whether the domain should be rated as “high”, “low” or “unclear” risk of bias. The guidance document contains further instructions and examples on rating signalling questions and risk of bias for each domain.  The first three domains are also rated for concerns regarding applicability (low/ high/ unclear) to your review question defined above.  *Complete all domains separately for each evaluation of a distinct model. Shaded boxes indicate where signalling questions do not apply and should not be answered.* |
| --- |

| **DOMAIN 1: Participants** | | | |
| --- | --- | --- | --- |
| **A. Risk of Bias** | | | |
| *Describe the sources of data and criteria for participant selection:*  The present study consisted of 1240 individuals charged with a criminal offense, and subsequently deemed either Unfit to Stand Trial (UST) or Not Criminally Responsible (NCR) as a result of serious mental illness. That comprised a diverse sample of patients from 10 forensic psychiatry facilities, representing patients who were subject to oversight by the Ontario Review Board (ORB) between 2014 and 2015. | | | |
|  | | Dev | Val |
| - 1. Were appropriate data sources used, e.g. cohort, RCT or nested case-control study data? | | N |  |
| - 1. Were all inclusions and exclusions of participants appropriate? | | NI |  |
| **Risk of bias introduced by selection of participants** | **RISK:**  *(low/ high/ unclear)* | **High** |  |
| *Rationale of bias rating:*  1.1 is N because the design is retrospective, consequently data were collected for other purposes. | | | |
| **B. Applicability** | | | |
| *Describe included participants, setting and dates:*  The present study consisted of 1240 individuals charged with a criminal offense, and subsequently deemed either Unfit to Stand Trial (UST) or Not Criminally Responsible (NCR) as a result of serious mental illness. That comprised a diverse sample of patients from 10 forensic psychiatry facilities, representing patients who were subject to oversight by the Ontario Review Board (ORB) between 2014 and 2015. | | | |
| **Concern that the included participants and setting do not match the review question** | **CONCERN:**  *(low/ high/ unclear)* | **Low** |  |
| *Rationale of applicability rating:*  Participants are representative of the population specified in the review question | | | |

| **DOMAIN 2: Predictors** | | | |
| --- | --- | --- | --- |
| **A. Risk of Bias** | | | |
| *List and describe predictors included in the final model, e.g. definition and timing of assessment:*  **138 variables** among which adverse events in childhood, income, housing, comorbidities, family history, prescribed medications, substance use, and presumed indicators of risk. Variables were transformed via one-hot encoding into new binary variables. This resulted in 156 candidate features. Only variables occurring prior to the index offense were considered as potential predictors | | | |
|  | | Dev | Val |
| - 1. Were predictors defined and assessed in a similar way for all participants? | | Y |  |
| - 1. Were predictor assessments made without knowledge of outcome data? | | PY |  |
| - 1. Are all predictors available at the time the model is intended to be used? | | Y |  |
| **Risk of bias introduced by predictors or their assessment** | **RISK:**  *(low/ high/ unclear)* | **Low** |  |
| *Rationale of bias rating:*  No issues emerged | | | |
| **B. Applicability** | | | |
| Concern that the definition, assessment or timing of predictors in the model do not match the review question | **CONCERN:**  *(low/ high/ unclear)* | **Low** |  |
| *Rationale of applicability rating:*  Included predictors appear representative of the predictors specified in the review question. | | | |

| **DOMAIN 3: Outcome** | | | |
| --- | --- | --- | --- |
| **A. Risk of Bias** | | | |
| *Describe the outcome, how it was defined and determined, and the time interval between predictor assessment and outcome determination:*  Patients were divided into Violent, Nonviolent and Sexual offenses according to the most recent criminal offense for which they were found not criminally responsible. In cases where multiple crimes were committed, patients were divided according to the most serious offense committed | | | |
|  | | Dev | Val |
| - 1. Was the outcome determined appropriately? | | Y |  |
| - 1. Was a pre-specified or standard outcome definition used? | | Y |  |
| - 1. Were predictors excluded from the outcome definition? | | NI |  |
| - 1. Was the outcome defined and determined in a similar way for all participants? | | Y |  |
| - 1. Was the outcome determined without knowledge of predictor information? | | PY |  |
| - 1. Was the time interval between predictor assessment and outcome determination appropriate? | | Y |  |
| **Risk of bias introduced by the outcome or its determination** | **RISK:**  *(low/ high/ unclear)* | **Low** |  |
| *Rationale of bias rating:*  Only variables occurring prior to the index offense were considered as potential predictors | | | |
| **B. Applicability** | | | |
| *At what time point was the outcome determined:*  Before the study  *If a composite outcome was used, describe the relative frequency/distribution of each contributing outcome:*  N/A | | | |
| **Concern that the outcome, its definition, timing or determination do not match the review question** | **CONCERN:**  *(low/ high/ unclear)* | **Low** |  |
| *Rationale of applicability rating:*  No issues emerged | | | |

| **DOMAIN 4: Analysis** | | | |
| --- | --- | --- | --- |
| **Risk of Bias** | | | |
| *Describe numbers of participants, number of candidate predictors, outcome events and events per candidate predictor:*  The present study consisted of 1240 individuals charged with a criminal offense, and subsequently deemed either Unfit to Stand Trial (UST) or Not Criminally Responsible (NCR) as a result of serious mental illness. That comprised a diverse sample of patients from 10 forensic psychiatry facilities, representing patients who were subject to oversight by the Ontario Review Board (ORB) between 2014 and 2015.  156 predictors  Violent = 863  Non violent = 253  Sexual = 124 | | | |
| *Describe how the model was developed (for example in regards to modelling technique (e.g. survival or logistic modelling), predictor selection, and risk group definition):*  In the present study, a data-driven approach to feature selection was used. This encompasses a series of feature selection methods that do not rely on preconceived notions as to which variables will be the most important in the model (Chandrashekar and Sahin, 2014). Specifically, three methods were compared. This included Recursive Feature Elimination (RFE), Ensemble Feature Selection (EFS), and selecting the top 20 weighting factors using variable importance plots. In all methods, feature selection was performed on training data only (70%). | | | |
| *Describe whether and how the model was validated, either internally (e.g. bootstrapping, cross validation, random split sample) or externally (e.g. temporal validation, geographical validation, different setting, different type of participants):*  The ORB dataset was divided into training and testing sets, comprising 70% and 30% of the data, respectively. In order to estimate prediction error, 10-fold cross-validation was used | | | |
| *Describe the performance measures of the model, e.g. (re)calibration, discrimination, (re)classification, net benefit, and whether they were adjusted for optimism:*  A receiver operating characteristic (ROC) curve and ‘confusion matrix’ were used to calculate the sensitivity, specificity, balanced accuracy, AUC, and 95% confidence intervals of each of these models. | | | |
| *Describe any participants who were excluded from the analysis:*  No information | | | |
| *Describe missing data on predictors and outcomes as well as methods used for missing data:*  Variables with 15% or more missing data were excluded, considering both the impact of missing values in model performance, and the limitations of available imputation strategies | | | |
|  | | Dev | Val |
| - 1. Were there a reasonable number of participants with the outcome? | | Y |  |
| - 1. Were continuous and categorical predictors handled appropriately? | | Y |  |
| - 1. Were all enrolled participants included in the analysis? | | Y |  |
| - 1. Were participants with missing data handled appropriately? | | Y |  |
| - 1. Was selection of predictors based on univariable analysis avoided? | | Y |  |
| - 1. Were complexities in the data (e.g. censoring, competing risks, sampling of controls) accounted for appropriately? | | Y |  |
| - 1. Were relevant model performance measures evaluated appropriately? | | Y |  |
| - 1. Were model overfitting and optimism in model performance accounted for? | | PY |  |
| - 1. Do predictors and their assigned weights in the final model correspond to the results from multivariable analysis? | | NI |  |
| **Risk of bias introduced by the analysis** | **RISK:**  *(low/ high/ unclear)* | **Low** |  |
| *Rationale of bias rating:*  No issues emerged | | | |

**Step 4: Overall assessment**

| Use the following tables to reach overall judgements about risk of bias and concerns regarding applicability of the prediction model evaluation (development and/or validation) across all assessed domains.  *Complete for each evaluation of a distinct model.*   \| **Reaching an overall judgement about risk of bias of the prediction model evaluation** \| \| \| --- \| --- \| \| **Low risk of bias** \| If all domains were rated low risk of bias.  If a prediction model was developed without any external validation, and it was rated as low risk of bias for all domains, consider downgrading to **high risk of bias**. Such a model can only be considered as low risk of bias, if the development was based on a very large data set and included some form of internal validation. \| \| **High risk of bias** \| If at least one domain is judged to be at **high risk of bias**. \| \| **Unclear risk of bias** \| If an unclear risk of bias was noted in at least one domain and it was low risk for all other domains. \|  \| **Reaching an overall judgement about applicability of the prediction model evaluation** \| \| \| --- \| --- \| \| **Low concerns regarding applicability** \| If low concerns regarding applicability for all domains, the prediction model evaluation is judged to have **low concerns regarding applicability**. \| \| **High concerns regarding applicability** \| If high concerns regarding applicability for at least one domain, the prediction model evaluation is judged to have **high concerns regarding applicability**. \| \| **Unclear concerns regarding applicability** \| If unclear concerns (but no “high concern”) regarding applicability for at least one domain, the prediction model evaluation is judged to have **unclear concerns regarding applicability** overall. \| |
| --- | --- | --- | --- | --- | --- | --- | --- | --- | --- | --- | --- | --- | --- | --- | --- | --- |

| **Overall judgement about risk of bias and applicability of the prediction model evaluation** | | |
| --- | --- | --- |
| **Overall judgement of risk of bias** | **RISK:**  *(low/ high/ unclear)* | **High** |
| *Summary of sources of potential bias:*  The design is retrospective, consequently data were collected for other purposes | | |
| **Overall judgement of applicability** | **CONCERN:**  *(low/ high/ unclear)* | **Low** |
| *Summary of applicability concerns:*  No issues emerged | | |

**PROBAST – Second evaluator**

(Prediction model study Risk Of Bias Assessment Tool)

Published in Annals of Internal Medicine (freely available):

1. [PROBAST: A Tool to Assess the Risk of Bias and Applicability of Prediction Model Studies](https://annals.org/aim/fullarticle/2719961/probast-tool-assess-risk-bias-applicability-prediction-model-studies)
2. [PROBAST: A Tool to Assess Risk of Bias and Applicability of Prediction Model Studies: Explanation and Elaboration](https://annals.org/aim/fullarticle/2719962/probast-tool-assess-risk-bias-applicability-prediction-model-studies-explanation)

| **What does PROBAST assess?**  PROBAST assesses both the *risk of bias* and *concerns regarding applicability* of a study that evaluates (develops, validates or updates) a multivariable diagnostic or prognostic prediction model. It is designed to assess primary studies included in a systematic review.  *Bias* occurs if systematic flaws or limitations in the design, conduct or analysis of a primary study distort the results. For the purpose of prediction modelling studies, we have defined *risk of bias* to occur when shortcomings in the study design, conduct or analysis lead to systematically distorted estimates of a model’s predictive performance or to an inadequate model to address the research question. Model predictive performance is typically evaluated using calibration, discrimination and sometimes classification measures, and these are likely inaccurately estimated in studies with high risk of bias. *Applicability* refers to the extent to which the prediction model from the primary study matches your systematic review question, for example in terms of the participants, predictors or outcome of interest.  A primary study may include the development and/or validation or update of more than one prediction model. A PROBAST assessment should be completed for each distinct model that is developed, validated or updated (extended) for making individualised predictions. Where a publication assesses multiple prediction models, only complete a PROBAST assessment for those models that meet the inclusion criteria for your systematic review. Please note that subsequent use of the term “model” includes derivatives of models, such as simplified risk scores, nomograms, or recalibrations of models.  PROBAST is not designed for all multivariable diagnostic or prognostic studies. For example, studies using multivariable models to identify predictors associated with an outcome but not attempting to develop a model for making individualised predictions are not covered by PROBAST.  PROBAST includes four steps.   \| **Step** \| **Task** \| **When to complete** \| \| --- \| --- \| --- \| \| **1** \| Specify your systematic review question(s) \| Once per systematic review \| \| **2** \| Classify the type of prediction model evaluation \| Once for each model of interest in each publication being assessed, for each relevant outcome \| \| **3** \| Assess risk of bias and applicability \| Once for each development and validation of each distinct prediction model in a publication \| \| **4** \| Overall judgment \| Once for each development and validation of each distinct prediction model in a publication \|   If this is your first time using PROBAST, we strongly recommend reading the detailed explanation and elaboration (E&E, see link above) paper and to check the examples on www.probast.org |
| --- | --- | --- | --- | --- | --- | --- | --- | --- | --- | --- | --- | --- | --- | --- | --- |

**Step 1: Specify your systematic review question**

| State your systematic review question to facilitate the assessment of the applicability of the evaluated models to your question. *The following table should be completed once per systematic review.* |
| --- |

| **Criteria** | **Specify your systematic review question** |
| --- | --- |
| *Intended use of model:* | Machine learning tecniques for predicting risk of aggression/violent behaviour |
| ***Participants*** *including selection criteria and setting:* | Psychiatric patients in clinical and forensic settings |
| ***Predictors*** *(used in prediction modelling), including types of predictors (e.g. history, clinical examination, biochemical markers, imaging tests), time of measurement, specific measurement issues (e.g., any requirements/ prohibitions for specialized equipment):* | Clinical, sociodemographic, and historical characteristics.  No specific measurement issues. |
| *Outcome to be predicted:* | Patients with violent behaviours and patients with no violent  behaviour as an outcome |

**Step 2: Classify the type of prediction model evaluation**

| Use the following table to classify the evaluation as model development, model validation or model update, or combination. Different signalling questions apply for different types of prediction model evaluation. If the evaluation does not fit one of these classifications then PROBAST should not be used. |
| --- |

| **Classify the evaluation based on its aim** | | | |
| --- | --- | --- | --- |
| **Type of prediction study** | **PROBAST boxes to complete** | **Tick as appropriate** | **Definition for type of prediction model study** |
| Development only | Development | V | Prediction model development without external validation. These studies may include internal validation methods, such as bootstrapping and cross-validation techniques. |
| Development and validation | Development and validation | X | Prediction model development combined with external validation in other participants in the same article. |
| Validation only | Validation | x | External validation of existing (previously developed) model in other participants. |

| *This table should be completed once for each publication being assessed and for each relevant outcome in your review.* | |  |
| --- | --- | --- |
| **Publication reference** | Gou, N., Xiang, Y., Zhou, J., Zhang, S., Zhong, S., Lu, J., Liang, X., Liu, J., & Wang, X. (2021). Identification of violent patients with schizophrenia using a hybrid machine learning approach at the individual level. Psychiatry research, 306, 114294. https://doi.org/10.1016/j.psychres.2021.114294 | |
| **Models of interest** | LASSO, SVM | |
| **Outcome of interest** | History of violence or not violence | |

**Step 3: Assess risk of bias and applicability**

| PROBAST is structured as four key domains. Each domain is judged for risk of bias (low, high or unclear) and includes signalling questions to help make judgements. Signalling questions are rated as yes (Y), probably yes (PY), probably no (PN), no (N) or no information (NI). All signalling questions are phrased so that “yes” indicates absence of bias. Any signalling question rated as “no” or “probably no” flags the potential for bias; you will need to use your judgement to determine whether the domain should be rated as “high”, “low” or “unclear” risk of bias. The guidance document contains further instructions and examples on rating signalling questions and risk of bias for each domain.  The first three domains are also rated for concerns regarding applicability (low/ high/ unclear) to your review question defined above.  *Complete all domains separately for each evaluation of a distinct model. Shaded boxes indicate where signalling questions do not apply and should not be answered.* |
| --- |

| **DOMAIN 1: Participants** | | | |
| --- | --- | --- | --- |
| **A. Risk of Bias** | | | |
| *Describe the sources of data and criteria for participant selection:*  Participants were recruited from the general psychiatric wards of forensic psychiatry department of the Second Xiangya Hospital of Central South University and from the general psychiatric wards of the psychiatry department. All the participants met the inclusion criteria: (1) male; (2) aged between 18 and 50 years; (3) with an IQ higher than 70; (4) with a diagnosis of schizophrenia confirmed using the International Classification of Diseases Version 10 (ICD-10). Exclusion criteria included comorbidity with any other diagnosis according to the ICD-10 diagnostic criteria, such as intellectual disability, major neurological disorders, history of head trauma (loss of consciousness for more than 5 min), and any MRI contraindication. | | | |
|  | | Dev | Val |
| - 1. Were appropriate data sources used, e.g. cohort, RCT or nested case-control study data? | | PY |  |
| - 1. Were all inclusions and exclusions of participants appropriate? | | Y |  |
| **Risk of bias introduced by selection of participants** | **RISK:**  *(low/ high/ unclear)* | **Low** |  |
| *Rationale of bias rating:* | | | |
| A cross-sectional approach was used in which study subjects were included based on their presence history of violence or not violence. Exclusions and inclusions criteria are appropriate. | | | |
| **B. Applicability** | | | |
| *Describe included participants, setting and dates:*  Participants with schizophrenia who were violent offenders (*n* = 42) and non-violent patients with schizophrenia (*n* = 32) were recruited from November 2011 to November 2020 from psychiatric departments. | | | |
| **Concern that the included participants and setting do not match the review question** | **CONCERN:**  *(low/ high/ unclear)* | **High** |  |
| *Rationale of applicability rating:* | | | |
| Participants and setting don’t seem to fit review question; Small sample size, female patients with schizophrenia were not included in this study, the dataset was derived from a single inpatient facility. | | | |

| **DOMAIN 2: Predictors** | | | |
| --- | --- | --- | --- |
| **A. Risk of Bias** | | | |
| *List and describe predictors included in the final model, e.g. definition and timing of assessment:*  Clinical characteristics, including times of psychiatric hospitalizations, medications, duration of illness and history of violent offences were recorded using a self-developed standard form. The Brief Psychiatric Rating Scale (BPRS) (Woerner et al., 1988) was used to estimate psychiatric symptoms. The Psychopathy Checklist: Screening Version (PCL-SV) (Hare et al., 1995) was used to estimate the patients’ _psychopathic personality. The Barratt Impulsiveness Scale version 11 (BIS-11) (Stanford et al., 2009) was used to measure individual impulsivity. The Historical, Clinical and Risk Management (HCR-20) (Telles et al., 2009) was used for violence risk assessment.  Neuroimaging data were collected using a 3-tesla (3T) MRI scanner (Philips Medical Systems). | | | |
|  | | Dev | Val |
| - 1. Were predictors defined and assessed in a similar way for all participants? | | Y |  |
| - 1. Were predictor assessments made without knowledge of outcome data? | | PN |  |
| - 1. Are all predictors available at the time the model is intended to be used? | | Y |  |
| **Risk of bias introduced by predictors or their assessment** | **RISK:**  *(low/ high/ unclear)* | **High** |  |
| *Rationale of bias rating:*  The items (predictors) were assessed similarly for all study subjects. We assigned 2.2. = PN because predictors and outcomes are assessed within a similar time frame. | | | |
| **B. Applicability** | | | |
| Concern that the definition, assessment or timing of predictors in the model do not match the review question | **CONCERN:**  *(low/ high/ unclear)* | **Low** |  |
| *Rationale of applicability rating:*  The definition and measurement of the predictors matches the review question and targeted context | | | |

| **DOMAIN 3: Outcome** | | | |
| --- | --- | --- | --- |
| **A. Risk of Bias** | | | |
| *Describe the outcome, how it was defined and determined, and the time interval between predictor assessment and outcome determination:*  “Violent offenders” referred to those who had committed violent crimes, including killing or assaulting other people (interpersonal violence), and were undergoing forensic psychiatric evaluation. The Modified Overt Aggression Scale (MOAS) (Margari et al., 2005) was used to quantify the overall degree of violence around 1 month before the assessment. The forensic patients had a score of ≥ 3 for item 4 (physical aggression scale); while the non-violent patients had a score of < 2 for item 4 and were free of any severe aggressive act against property and/or themselves. | | | |
|  | | Dev | Val |
| - 1. Was the outcome determined appropriately? | | Y |  |
| - 1. Was a pre-specified or standard outcome definition used? | | Y |  |
| - 1. Were predictors excluded from the outcome definition? | | PY |  |
| - 1. Was the outcome defined and determined in a similar way for all participants? | | Y |  |
| - 1. Was the outcome determined without knowledge of predictor information? | | PY |  |
| - 1. Was the time interval between predictor assessment and outcome determination appropriate? | | PY |  |
| **Risk of bias introduced by the outcome or its determination** | **RISK:**  *(low/ high/ unclear)* | **Low** |  |
| *Rationale of bias rating:*  All items of this domain were explicitly or implicitly addressed but the data are Retrospective | | | |
| **B. Applicability** | | | |
| *At what time point was the outcome determined:*  Discrimination between patients with a history of violence and non-violence. Predictors and outcomes are assessed within a similar time frame.  *If a composite outcome was used, describe the relative frequency/distribution of each contributing outcome:*  N/A | | | |
| **Concern that the outcome, its definition, timing or determination do not match the review question** | **CONCERN:**  *(low/ high/ unclear)* | **Low** |  |
| *Rationale of applicability rating:*  The definition, measurement and timing of the outcome matches the review question and targeted context | | | |

| **DOMAIN 4: Analysis** | | | |
| --- | --- | --- | --- |
| **Risk of Bias** | | | |
| *Describe numbers of participants, number of candidate predictors, outcome events and events per candidate predictor:*  *74 participants, n=9 candidate predictors,* violent offenders (*n* = 42) and non-violent patients with schizophrenia (*n* = 32). | | | |
| *Describe how the model was developed (for example in regards to modelling technique (e.g. survival or logistic modelling), predictor selection, and risk group definition):*  In order to achieve a superior prediction performance, fusion at both feature level and decision-making level were applied to obtain the most relevant individualized information of multi-level features. At the feature fusion level, a multi-stage feature fusion framework was constructed. At the decision-making fusion level, the three modality fusion features and sociodemographic-clinical features consisting of the scores on the BPRS hostility, the PCL-SV and HCR-20 total scores and education years were combined using a soft voting method to form a precise prediction model for violence in schizophrenia. Finally, we examined the capability of the model by combining the neuroimaging and sociodemographic-clinical features using a soft voting method, which suggested that the classifier was superior to other models, with an ACC of 90.67%, SEN of 90.91%, SPE of 90.48%, and AUC of 0.95 | | | |
| *Describe whether and how the model was validated, either internally (e.g. bootstrapping, cross validation, random split sample) or externally (e.g. temporal validation, geographical validation, different setting, different type of participants):*  Leave-One-Out Cross Validation (LOOCV) method was adopted. | | | |
| *Describe the performance measures of the model, e.g. (re)calibration, discrimination, (re)classification, net benefit, and whether they were adjusted for optimism:*  Four metrics (i.e., accuracy, sensitivity, specificity and area under the receiver operating characteristic (ROC) curve [AUC]) were further calculated to fairly measure the classification performance of the corresponding method. | | | |
| *Describe any participants who were excluded from the analysis:*  One patient was comorbid with substance abuse and this subject was excluded. | | | |
| *Describe missing data on predictors and outcomes as well as methods used for missing data:*  *MOAS-total score (n=62) for VSZ, data were missing for 1 patient; for NVSZ, data were missing for 12 patients.*  *Inpatient admission (n=69), for VSZ, data were missing for 5 patients; for NVSZ, data were missing for 1 patient. No explicit method used for missing data.* | | | |
|  | | Dev | Val |
| - 1. Were there a reasonable number of participants with the outcome? | | PN |  |
| - 1. Were continuous and categorical predictors handled appropriately? | | Y |  |
| - 1. Were all enrolled participants included in the analysis? | | Y |  |
| - 1. Were participants with missing data handled appropriately? | | NI |  |
| - 1. Was selection of predictors based on univariable analysis avoided? | | Y |  |
| - 1. Were complexities in the data (e.g. censoring, competing risks, sampling of controls) accounted for appropriately? | | PY |  |
| - 1. Were relevant model performance measures evaluated appropriately? | | Y |  |
| - 1. Were model overfitting and optimism in model performance accounted for? | | Y |  |
| - 1. Do predictors and their assigned weights in the final model correspond to the results from multivariable analysis? | | NI |  |
| **Risk of bias introduced by the analysis** | **RISK:**  *(low/ high/ unclear)* | **High** |  |
| *Rationale of bias rating:*  At least 9 different candidate predictors considered but only 42 participants with an event. | | | |

**Step 4: Overall assessment**

| Use the following tables to reach overall judgements about risk of bias and concerns regarding applicability of the prediction model evaluation (development and/or validation) across all assessed domains.  *Complete for each evaluation of a distinct model.*   \| **Reaching an overall judgement about risk of bias of the prediction model evaluation** \| \| \| --- \| --- \| \| **Low risk of bias** \| If all domains were rated low risk of bias.  If a prediction model was developed without any external validation, and it was rated as low risk of bias for all domains, consider downgrading to **high risk of bias**. Such a model can only be considered as low risk of bias, if the development was based on a very large data set and included some form of internal validation. \| \| **High risk of bias** \| If at least one domain is judged to be at **high risk of bias**. \| \| **Unclear risk of bias** \| If an unclear risk of bias was noted in at least one domain and it was low risk for all other domains. \|  \| **Reaching an overall judgement about applicability of the prediction model evaluation** \| \| \| --- \| --- \| \| **Low concerns regarding applicability** \| If low concerns regarding applicability for all domains, the prediction model evaluation is judged to have **low concerns regarding applicability**. \| \| **High concerns regarding applicability** \| If high concerns regarding applicability for at least one domain, the prediction model evaluation is judged to have **high concerns regarding applicability**. \| \| **Unclear concerns regarding applicability** \| If unclear concerns (but no “high concern”) regarding applicability for at least one domain, the prediction model evaluation is judged to have **unclear concerns regarding applicability** overall. \| |
| --- | --- | --- | --- | --- | --- | --- | --- | --- | --- | --- | --- | --- | --- | --- | --- | --- |

| **Overall judgement about risk of bias and applicability of the prediction model evaluation** | | |
| --- | --- | --- |
| **Overall judgement of risk of bias** | **RISK:**  *(low/ high/ unclear)* | **High** |
| *Summary of sources of potential bias:*  Any external validation. Opportunity sampling. Retrospective outcome. | | |
| **Overall judgement of applicability** | **CONCERN:**  *(low/ high/ unclear)* | **High** |
| *Summary of applicability concerns:*  Participants and setting don’t seem to fit review question; Small sample size, female patients with schizophrenia were not included in this study, the dataset was derived from a single inpatient facility. | | |

**PROBAST – Second evaluator**

(Prediction model study Risk Of Bias Assessment Tool)

Published in Annals of Internal Medicine (freely available):

1. [PROBAST: A Tool to Assess the Risk of Bias and Applicability of Prediction Model Studies](https://annals.org/aim/fullarticle/2719961/probast-tool-assess-risk-bias-applicability-prediction-model-studies)
2. [PROBAST: A Tool to Assess Risk of Bias and Applicability of Prediction Model Studies: Explanation and Elaboration](https://annals.org/aim/fullarticle/2719962/probast-tool-assess-risk-bias-applicability-prediction-model-studies-explanation)

| **What does PROBAST assess?**  PROBAST assesses both the *risk of bias* and *concerns regarding applicability* of a study that evaluates (develops, validates or updates) a multivariable diagnostic or prognostic prediction model. It is designed to assess primary studies included in a systematic review.  *Bias* occurs if systematic flaws or limitations in the design, conduct or analysis of a primary study distort the results. For the purpose of prediction modelling studies, we have defined *risk of bias* to occur when shortcomings in the study design, conduct or analysis lead to systematically distorted estimates of a model’s predictive performance or to an inadequate model to address the research question. Model predictive performance is typically evaluated using calibration, discrimination and sometimes classification measures, and these are likely inaccurately estimated in studies with high risk of bias. *Applicability* refers to the extent to which the prediction model from the primary study matches your systematic review question, for example in terms of the participants, predictors or outcome of interest.  A primary study may include the development and/or validation or update of more than one prediction model. A PROBAST assessment should be completed for each distinct model that is developed, validated or updated (extended) for making individualised predictions. Where a publication assesses multiple prediction models, only complete a PROBAST assessment for those models that meet the inclusion criteria for your systematic review. Please note that subsequent use of the term “model” includes derivatives of models, such as simplified risk scores, nomograms, or recalibrations of models.  PROBAST is not designed for all multivariable diagnostic or prognostic studies. For example, studies using multivariable models to identify predictors associated with an outcome but not attempting to develop a model for making individualised predictions are not covered by PROBAST.  PROBAST includes four steps.   \| **Step** \| **Task** \| **When to complete** \| \| --- \| --- \| --- \| \| **1** \| Specify your systematic review question(s) \| Once per systematic review \| \| **2** \| Classify the type of prediction model evaluation \| Once for each model of interest in each publication being assessed, for each relevant outcome \| \| **3** \| Assess risk of bias and applicability \| Once for each development and validation of each distinct prediction model in a publication \| \| **4** \| Overall judgment \| Once for each development and validation of each distinct prediction model in a publication \|   If this is your first time using PROBAST, we strongly recommend reading the detailed explanation and elaboration (E&E, see link above) paper and to check the examples on www.probast.org |
| --- | --- | --- | --- | --- | --- | --- | --- | --- | --- | --- | --- | --- | --- | --- | --- |

**Step 1: Specify your systematic review question**

| State your systematic review question to facilitate the assessment of the applicability of the evaluated models to your question. *The following table should be completed once per systematic review.* |
| --- |

| **Criteria** | **Specify your systematic review question** |
| --- | --- |
| *Intended use of model:* | Machine learning tecniques for predicting risk of aggression/violent behaviour |
| ***Participants*** *including selection criteria and setting:* | Psychiatric patients in clinical and forensic settings |
| ***Predictors*** *(used in prediction modelling), including types of predictors (e.g. history, clinical examination, biochemical markers, imaging tests), time of measurement, specific measurement issues (e.g., any requirements/ prohibitions for specialized equipment):* | Clinical, sociodemographic, and historical characteristics.  No specific measurement issues. |
| *Outcome to be predicted:* | Patients with violent behaviours and patients with no violent  behaviour as an outcome |

**Step 2: Classify the type of prediction model evaluation**

| Use the following table to classify the evaluation as model development, model validation or model update, or combination. Different signalling questions apply for different types of prediction model evaluation. If the evaluation does not fit one of these classifications then PROBAST should not be used. |
| --- |

| **Classify the evaluation based on its aim** | | | |
| --- | --- | --- | --- |
| **Type of prediction study** | **PROBAST boxes to complete** | **Tick as appropriate** | **Definition for type of prediction model study** |
| Development only | Development | V | Prediction model development without external validation. These studies may include internal validation methods, such as bootstrapping and cross-validation techniques. |
| Development and validation | Development and validation | X | Prediction model development combined with external validation in other participants in the same article. |
| Validation only | Validation | x | External validation of existing (previously developed) model in other participants. |

| *This table should be completed once for each publication being assessed and for each relevant outcome in your review.* | |  |
| --- | --- | --- |
| **Publication reference** | Kirchebner, J., Sonnweber, M., Nater, U. M., Günther, M., & Lau, S. (2022). Stress, Schizophrenia, and Violence: A Machine Learning Approach. Journal of interpersonal violence, 37(1-2), 602–622. | |
| **Models of interest** | SVM, KNN, logistic regression, trees | |
| **Outcome of interest** | Analyze the impact of accumulation and type of stressor on committing an offense in patients with schizophrenia spectrum disorders | |

**Step 3: Assess risk of bias and applicability**

| PROBAST is structured as four key domains. Each domain is judged for risk of bias (low, high or unclear) and includes signalling questions to help make judgements. Signalling questions are rated as yes (Y), probably yes (PY), probably no (PN), no (N) or no information (NI). All signalling questions are phrased so that “yes” indicates absence of bias. Any signalling question rated as “no” or “probably no” flags the potential for bias; you will need to use your judgement to determine whether the domain should be rated as “high”, “low” or “unclear” risk of bias. The guidance document contains further instructions and examples on rating signalling questions and risk of bias for each domain.  The first three domains are also rated for concerns regarding applicability (low/ high/ unclear) to your review question defined above.  *Complete all domains separately for each evaluation of a distinct model. Shaded boxes indicate where signalling questions do not apply and should not be answered.* |
| --- |

| **DOMAIN 1: Participants** | | | |
| --- | --- | --- | --- |
| **A. Risk of Bias** | | | |
| *Describe the sources of data and criteria for participant selection:*  Directed qualitative content analysis was conducted on inpatients’ medical records from 1982 to 2016, the content analysis employed an objective coding protocol based on an extended set of criteria by Seifert and Leygraf and was augmented both by previous research findings and by consultation among the authors regarding the factors to be included. | | | |
|  | | Dev | Val |
| - 1. Were appropriate data sources used, e.g. cohort, RCT or nested case-control study data? | | PN |  |
| - 1. Were all inclusions and exclusions of participants appropriate? | | NI |  |
| **Risk of bias introduced by selection of participants** | **RISK:**  *(low/ high/ unclear)* | **High** |  |
| *Rationale of bias rating:* | | | |
| The analysis was based on retrospectively collected data. No criteria for inpatients’ medical records. | | | |
| **B. Applicability** | | | |
| *Describe included participants, setting and dates:*  The sample comprised offenders (n = 370) who were hospitalized with a diagnosis of a SSD including schizophrenia, schizoaffective disorder, and delusional disorder given by their psychiatrist at discharge.  Setting: The Centre for Inpatient Forensic Therapy at the Zurich University Hospital of Psychiatry is an institution for the treatment of mentally ill offenders. Patients are admitted either for the treatment of acute syndromes or for longterm treatment to reduce the risk of reoffending due to their mental illness.  The data were collected from the inpatients’ medical records from 1982 to 2016, with the majority being recorded after the year 2000 (296 cases) | | | |
| **Concern that the included participants and setting do not match the review question** | **CONCERN:**  *(low/ high/ unclear)* | **High** |  |
| *Rationale of applicability rating:* | | | |
| Included patients don’t appear representative of the population specified in the review question.  Small size | | | |

| **DOMAIN 2: Predictors** | | | |
| --- | --- | --- | --- |
| **A. Risk of Bias** | | | |
| *List and describe predictors included in the final model, e.g. definition and timing of assessment:*  1) Divorce / Separation of caregivers in patient's childhood / youth  2) Severe physical illness of the patient in childhood / youth  3) Separation from the family/caregivers in the patient's childhood / youth  4) Physical or sexual abuse by the caregiver against the patient in his childhood/youth  5) Impairment of parent-child relationship  6) Rejection / being ignored by the caregiver  7) Active devaluation by the caregiver  8) Physical abuse by the caregiver  9) Poor parenting methods  10) Poverty in the patient's childhood / youth  11) Bullying by peer group in patient's childhood / youth  12) Failure in school  13) At least three previous psychiatric hospitalizations before investigated offense  14) Homelessness in adulthood  15) Conflicts in the workplace  16) Social isolation in adulthood  17) Violent victimization in adulthood  18) Coercive psychiatric treatment  19) Compulsory placement  20) Positive symptoms during criminal offense | | | |
|  | | Dev | Val |
| - 1. Were predictors defined and assessed in a similar way for all participants? | | Y |  |
| - 1. Were predictor assessments made without knowledge of outcome data? | | PY |  |
| - 1. Are all predictors available at the time the model is intended to be used? | | Y |  |
| **Risk of bias introduced by predictors or their assessment** | **RISK:**  *(low/ high/ unclear)* | **Low** |  |
| *Rationale of bias rating:*  No major issues identified. | | | |
| **B. Applicability** | | | |
| Concern that the definition, assessment or timing of predictors in the model do not match the review question | **CONCERN:**  *(low/ high/ unclear)* | **Low** |  |
| *Rationale of applicability rating:*  Predictors were assessed at presentation and defined in a standard way. They appear to match the review question. | | | |

| **DOMAIN 3: Outcome** | | | |
| --- | --- | --- | --- |
| **A. Risk of Bias** | | | |
| *Describe the outcome, how it was defined and determined, and the time interval between predictor assessment and outcome determination:*  The dependent variable—severity of the index offense—was dichotomized into (a) violent offense and (b) nonviolent offense. The following offenses were considered as violent based on Swiss law: homicide and attempted homicide, assault, rape, robbery, arson, and child abuse. The category nonviolent offense included threat, theft, damage to property, minor sexual offenses (e.g., exhibitionism), drug offenses, illegal gun possession, and other minor offenses (e.g., triggering false alarms or emergency brakes). One patient showed missing data on his index offense and was therefore excluded from the study. Of the remaining patients, 294  (79.7%) had committed a violent index offense and 75 (20.3%) had committed a nonviolent index offense | | | |
|  | | Dev | Val |
| - 1. Was the outcome determined appropriately? | | Y |  |
| - 1. Was a pre-specified or standard outcome definition used? | | Y |  |
| - 1. Were predictors excluded from the outcome definition? | | Y |  |
| - 1. Was the outcome defined and determined in a similar way for all participants? | | Y |  |
| - 1. Was the outcome determined without knowledge of predictor information? | | NI |  |
| - 1. Was the time interval between predictor assessment and outcome determination appropriate? | | PY |  |
| **Risk of bias introduced by the outcome or its determination** | **RISK:**  *(low/ high/ unclear)* | **Low** |  |
| *Rationale of bias rating:*  No concerns. | | | |
| **B. Applicability** | | | |
| *At what time point was the outcome determined:*  Discrimination between patients that had committed a violent index offense or a nonviolent index offense. Predictors and outcomes are assessed within a similar time frame.  *If a composite outcome was used, describe the relative frequency/distribution of each contributing outcome:*  N/A | | | |
| **Concern that the outcome, its definition, timing or determination do not match the review question** | **CONCERN:**  *(low/ high/ unclear)* | **Low** |  |
| *Rationale of applicability rating:*  The definition, measurement and timing of the outcome matches the review question and targeted context | | | |

| **DOMAIN 4: Analysis** | | | |
| --- | --- | --- | --- |
| **Risk of Bias** | | | |
| *Describe numbers of participants, number of candidate predictors, outcome events and events per candidate predictor:*  370 participants, 21candidate predictors, 294 (79.7%) had committed a violent index offense and 75 (20.3%) had committed a nonviolent index offense. | | | |
| *Describe how the model was developed (for example in regards to modelling technique (e.g. survival or logistic modelling), predictor selection, and risk group definition):*  Algorithm selection and performance testing were conducted using MATLAB (The MathWorks, 2012). Binary logistic regression and evaluation of the variable importance via the selected algorithm were both performed in R Studio version 1.1.383. To identify stressors that have explanatory power regarding offense severity, a supervised ML with the outcome variable violent offense/nonviolent offense and all 21 possible predictor variables to identify the most accurate algorithm (logistic regression, support vector machine [SVM], trees, and KNN algorithms) was performed | | | |
| *Describe whether and how the model was validated, either internally (e.g. bootstrapping, cross validation, random split sample) or externally (e.g. temporal validation, geographical validation, different setting, different type of participants):*  Cross-validation: The training set was divided into five equal-sized subsets, with one subset being used to train a model and the other four being used to evaluate the accuracy of the learned model (fivefold cross-validation). | | | |
| *Describe the performance measures of the model, e.g. (re)calibration, discrimination, (re)classification, net benefit, and whether they were adjusted for optimism:*  The goodness of fit was assessed using the receiver operating characteristic (ROC) curve method. The AUC served as the criterion to determine the level of discrimination again. In addition, the specificity and sensitivity, positive predictive value (PPV), and negative predictive value (NPV) were calculated. | | | |
| *Describe any participants who were excluded from the analysis:*  One patient showed missing data on his index offense and was therefore excluded from the study. | | | |
| *Describe missing data on predictors and outcomes as well as methods used for missing data:*  They used Boosted Classification Trees that are accommodate well to missing data. There is no specific information about missing data. | | | |
|  | | Dev | Val |
| - 1. Were there a reasonable number of participants with the outcome? | | Y |  |
| - 1. Were continuous and categorical predictors handled appropriately? | | PY |  |
| - 1. Were all enrolled participants included in the analysis? | | N |  |
| - 1. Were participants with missing data handled appropriately? | | PY |  |
| - 1. Was selection of predictors based on univariable analysis avoided? | | Y |  |
| - 1. Were complexities in the data (e.g. censoring, competing risks, sampling of controls) accounted for appropriately? | | PY |  |
| - 1. Were relevant model performance measures evaluated appropriately? | | Y |  |
| - 1. Were model overfitting and optimism in model performance accounted for? | | NI |  |
| - 1. Do predictors and their assigned weights in the final model correspond to the results from multivariable analysis? | | PY |  |
| **Risk of bias introduced by the analysis** | **RISK:**  *(low/ high/ unclear)* | **Low** |  |
| *Rationale of bias rating:*  All items of this domain were explicitly or implicitly addressed | | | |

**Step 4: Overall assessment**

| Use the following tables to reach overall judgements about risk of bias and concerns regarding applicability of the prediction model evaluation (development and/or validation) across all assessed domains.  *Complete for each evaluation of a distinct model.*   \| **Reaching an overall judgement about risk of bias of the prediction model evaluation** \| \| \| --- \| --- \| \| **Low risk of bias** \| If all domains were rated low risk of bias.  If a prediction model was developed without any external validation, and it was rated as low risk of bias for all domains, consider downgrading to **high risk of bias**. Such a model can only be considered as low risk of bias, if the development was based on a very large data set and included some form of internal validation. \| \| **High risk of bias** \| If at least one domain is judged to be at **high risk of bias**. \| \| **Unclear risk of bias** \| If an unclear risk of bias was noted in at least one domain and it was low risk for all other domains. \|  \| **Reaching an overall judgement about applicability of the prediction model evaluation** \| \| \| --- \| --- \| \| **Low concerns regarding applicability** \| If low concerns regarding applicability for all domains, the prediction model evaluation is judged to have **low concerns regarding applicability**. \| \| **High concerns regarding applicability** \| If high concerns regarding applicability for at least one domain, the prediction model evaluation is judged to have **high concerns regarding applicability**. \| \| **Unclear concerns regarding applicability** \| If unclear concerns (but no “high concern”) regarding applicability for at least one domain, the prediction model evaluation is judged to have **unclear concerns regarding applicability** overall. \| |
| --- | --- | --- | --- | --- | --- | --- | --- | --- | --- | --- | --- | --- | --- | --- | --- | --- |

| **Overall judgement about risk of bias and applicability of the prediction model evaluation** | | |
| --- | --- | --- |
| **Overall judgement of risk of bias** | **RISK:**  *(low/ high/ unclear)* | **High** |
| *Summary of sources of potential bias:*  The present analysis was based on retrospectively collected data, and it was not possible to assess the subjective perspectives of patients and physicians in a standardized manner or the time courses of the respective stressors, as there was no indication of their duration. The fact that the data were not derived from a longitudinal sample limits the ability to make causal inferences. Our sample consisted mainly of male subjects, which reduces the generalizability of the results. | | |
| **Overall judgement of applicability** | **CONCERN:**  *(low/ high/ unclear)* | **High** |
| *Summary of applicability concerns:*  Included patients don’t appear representative of the population specified in the review question. Small size. | | |

**PROBAST – Second evaluator**

(Prediction model study Risk Of Bias Assessment Tool)

Published in Annals of Internal Medicine (freely available):

1. [PROBAST: A Tool to Assess the Risk of Bias and Applicability of Prediction Model Studies](https://annals.org/aim/fullarticle/2719961/probast-tool-assess-risk-bias-applicability-prediction-model-studies)
2. [PROBAST: A Tool to Assess Risk of Bias and Applicability of Prediction Model Studies: Explanation and Elaboration](https://annals.org/aim/fullarticle/2719962/probast-tool-assess-risk-bias-applicability-prediction-model-studies-explanation)

| **What does PROBAST assess?**  PROBAST assesses both the *risk of bias* and *concerns regarding applicability* of a study that evaluates (develops, validates or updates) a multivariable diagnostic or prognostic prediction model. It is designed to assess primary studies included in a systematic review.  *Bias* occurs if systematic flaws or limitations in the design, conduct or analysis of a primary study distort the results. For the purpose of prediction modelling studies, we have defined *risk of bias* to occur when shortcomings in the study design, conduct or analysis lead to systematically distorted estimates of a model’s predictive performance or to an inadequate model to address the research question. Model predictive performance is typically evaluated using calibration, discrimination and sometimes classification measures, and these are likely inaccurately estimated in studies with high risk of bias. *Applicability* refers to the extent to which the prediction model from the primary study matches your systematic review question, for example in terms of the participants, predictors or outcome of interest.  A primary study may include the development and/or validation or update of more than one prediction model. A PROBAST assessment should be completed for each distinct model that is developed, validated or updated (extended) for making individualised predictions. Where a publication assesses multiple prediction models, only complete a PROBAST assessment for those models that meet the inclusion criteria for your systematic review. Please note that subsequent use of the term “model” includes derivatives of models, such as simplified risk scores, nomograms, or recalibrations of models.  PROBAST is not designed for all multivariable diagnostic or prognostic studies. For example, studies using multivariable models to identify predictors associated with an outcome but not attempting to develop a model for making individualised predictions are not covered by PROBAST.  PROBAST includes four steps.   \| **Step** \| **Task** \| **When to complete** \| \| --- \| --- \| --- \| \| **1** \| Specify your systematic review question(s) \| Once per systematic review \| \| **2** \| Classify the type of prediction model evaluation \| Once for each model of interest in each publication being assessed, for each relevant outcome \| \| **3** \| Assess risk of bias and applicability \| Once for each development and validation of each distinct prediction model in a publication \| \| **4** \| Overall judgment \| Once for each development and validation of each distinct prediction model in a publication \|   If this is your first time using PROBAST, we strongly recommend reading the detailed explanation and elaboration (E&E, see link above) paper and to check the examples on www.probast.org |
| --- | --- | --- | --- | --- | --- | --- | --- | --- | --- | --- | --- | --- | --- | --- | --- |

**Step 1: Specify your systematic review question**

| State your systematic review question to facilitate the assessment of the applicability of the evaluated models to your question. *The following table should be completed once per systematic review.* |
| --- |

| **Criteria** | **Specify your systematic review question** |
| --- | --- |
| *Intended use of model:* | Machine learning tecniques for predicting risk of aggression/violent behaviour |
| ***Participants*** *including selection criteria and setting:* | Psychiatric patients in clinical and forensic settings |
| ***Predictors*** *(used in prediction modelling), including types of predictors (e.g. history, clinical examination, biochemical markers, imaging tests), time of measurement, specific measurement issues (e.g., any requirements/ prohibitions for specialized equipment):* | Clinical, sociodemographic, and historical characteristics.  No specific measurement issues. |
| *Outcome to be predicted:* | Patients with violent behaviours and patients with no violent  behaviour as an outcome |

**Step 2: Classify the type of prediction model evaluation**

| Use the following table to classify the evaluation as model development, model validation or model update, or combination. Different signalling questions apply for different types of prediction model evaluation. If the evaluation does not fit one of these classifications then PROBAST should not be used. |
| --- |

| **Classify the evaluation based on its aim** | | | |
| --- | --- | --- | --- |
| **Type of prediction study** | **PROBAST boxes to complete** | **Tick as appropriate** | **Definition for type of prediction model study** |
| Development only | Development | V | Prediction model development without external validation. These studies may include internal validation methods, such as bootstrapping and cross-validation techniques. |
| Development and validation | Development and validation | X | Prediction model development combined with external validation in other participants in the same article. |
| Validation only | Validation | x | External validation of existing (previously developed) model in other participants. |

| *This table should be completed once for each publication being assessed and for each relevant outcome in your review.* | |  |
| --- | --- | --- |
| **Publication reference** | Lu, H., Xie, C., Lian, P., Yu, C., & Xie, Y. (2021). Psychosocial Factors Predict the Level of Aggression of People with Drug Addiction: A Machine Learning Approach. Psychology, Health & Medicine, 1–8. | |
| **Models of interest** | Gradient Boosting Regression Tree (GBRT) | |
| **Outcome of interest** | This study aimed to identify the relevant psychosocial factors that can predict the aggression in people with drug addiction. | |

**Step 3: Assess risk of bias and applicability**

| PROBAST is structured as four key domains. Each domain is judged for risk of bias (low, high or unclear) and includes signalling questions to help make judgements. Signalling questions are rated as yes (Y), probably yes (PY), probably no (PN), no (N) or no information (NI). All signalling questions are phrased so that “yes” indicates absence of bias. Any signalling question rated as “no” or “probably no” flags the potential for bias; you will need to use your judgement to determine whether the domain should be rated as “high”, “low” or “unclear” risk of bias. The guidance document contains further instructions and examples on rating signalling questions and risk of bias for each domain.  The first three domains are also rated for concerns regarding applicability (low/ high/ unclear) to your review question defined above.  *Complete all domains separately for each evaluation of a distinct model. Shaded boxes indicate where signalling questions do not apply and should not be answered.* |
| --- |

| **DOMAIN 1: Participants** | | | |
| --- | --- | --- | --- |
| **A. Risk of Bias** | | | |
| *Describe the sources of data and criteria for participant selection:*  The participants in this study were recruited from Zengcheng Compulsory Isolated Detoxification Center in Guangdong province, southern China, using cluster sampling. People who meet the following criteria are not eligible for the study: Unable to understand the terms in the questionnaire and leaving more than 30% items uncompleted. | | | |
|  | | Dev | Val |
| - 1. Were appropriate data sources used, e.g. cohort, RCT or nested case-control study data? | | PN |  |
| - 1. Were all inclusions and exclusions of participants appropriate? | | PN |  |
| **Risk of bias introduced by selection of participants** | **RISK:**  *(low/ high/ unclear)* | **High** |  |
| *Rationale of bias rating:* | | | |
| Cluster sampling is prone to bias. Generally, the samples drawn using the cluster method are prone to higher sampling error than the samples formed using other sampling methods. Exclusion criteria may be overstated | | | |
| **B. Applicability** | | | |
| *Describe included participants, setting and dates:*  A total of 896 participants (Mean*age* = 38.30, SD = 8.38) participated in the study. Clinical setting. | | | |
| **Concern that the included participants and setting do not match the review question** | **CONCERN:**  *(low/ high/ unclear)* | **High** |  |
| *Rationale of applicability rating:* | | | |
| Participants don’t seem to representative of the population of review question | | | |

| **DOMAIN 2: Predictors** | | | |
| --- | --- | --- | --- |
| **A. Risk of Bias** | | | |
| *List and describe predictors included in the final model, e.g. definition and timing of assessment:*  *Predictors are unclear* | | | |
|  | | Dev | Val |
| - 1. Were predictors defined and assessed in a similar way for all participants? | | NI |  |
| - 1. Were predictor assessments made without knowledge of outcome data? | | NI |  |
| - 1. Are all predictors available at the time the model is intended to be used? | | Y |  |
| **Risk of bias introduced by predictors or their assessment** | **RISK:**  *(low/ high/ unclear)* | **Unclear** |  |
| *Rationale of bias rating:*  Predictors weren’t defined and they weren’t’ blinded for the outcome | | | |
| **B. Applicability** | | | |
| Concern that the definition, assessment or timing of predictors in the model do not match the review question | **CONCERN:**  *(low/ high/ unclear)* | **Low** |  |
| *Rationale of applicability rating:*  No concerns | | | |

| **DOMAIN 3: Outcome** | | | |
| --- | --- | --- | --- |
| **A. Risk of Bias** | | | |
| *Describe the outcome, how it was defined and determined, and the time interval between predictor assessment and outcome determination:*  *It’s not clear the measure of the level of aggression.* | | | |
|  | | Dev | Val |
| - 1. Was the outcome determined appropriately? | | NI |  |
| - 1. Was a pre-specified or standard outcome definition used? | | NI |  |
| - 1. Were predictors excluded from the outcome definition? | | NI |  |
| - 1. Was the outcome defined and determined in a similar way for all participants? | | PY |  |
| - 1. Was the outcome determined without knowledge of predictor information? | | PY |  |
| - 1. Was the time interval between predictor assessment and outcome determination appropriate? | | NI |  |
| **Risk of bias introduced by the outcome or its determination** | **RISK:**  *(low/ high/ unclear)* | **Unclear** |  |
| *Rationale of bias rating:*  The outcome *wasn’t determined appropriately.* | | | |
| **B. Applicability** | | | |
| *At what time point was the outcome determined:*  Predictors and outcomes aren’t clear  *If a composite outcome was used, describe the relative frequency/distribution of each contributing outcome:* | | | |
| **Concern that the outcome, its definition, timing or determination do not match the review question** | **CONCERN:**  *(low/ high/ unclear)* | **High** |  |
| *Rationale of applicability rating:*  Outcome *wasn’t defined.* | | | |

| **DOMAIN 4: Analysis** | | | |
| --- | --- | --- | --- |
| **Risk of Bias** | | | |
| *Describe numbers of participants, number of candidate predictors, outcome events and events per candidate predictor:*  896 participants, 20 predictors, not specified event. | | | |
| *Describe how the model was developed (for example in regards to modelling technique (e.g. survival or logistic modelling), predictor selection, and risk group definition):*  The present study utilizes the Gradient Boosting Regression Tree (GBRT), a machine learning algorithm, to predict aggression with several psychosocial factors as well as Factor Importance to assess the relative importance of a specific feature according to the predicting contribution of the target variable. | | | |
| *Describe whether and how the model was validated, either internally (e.g. bootstrapping, cross validation, random split sample) or externally (e.g. temporal validation, geographical validation, different setting, different type of participants):*  The authors utilize fivefold cross-validation or out-of-sample testing techniques to estimate the accuracy of the predictive model. Cross-validation divides the data into the given number of subsets, performs the algorithm on the training dataset and then validates its performance on the testing dataset. The validation results are averaged over five rounds to provide an estimate of the model’s predictive performance. | | | |
| *Describe the performance measures of the model, e.g. (re)calibration, discrimination, (re)classification, net benefit, and whether they were adjusted for optimism:* | | | |
| *Describe any participants who were excluded from the analysis:*  NA | | | |
| *Describe missing data on predictors and outcomes as well as methods used for missing data:*  Missing values of the included data will be replaced with averages. | | | |
|  | | Dev | Val |
| - 1. Were there a reasonable number of participants with the outcome? | | NI |  |
| - 1. Were continuous and categorical predictors handled appropriately? | | NI |  |
| - 1. Were all enrolled participants included in the analysis? | | Y |  |
| - 1. Were participants with missing data handled appropriately? | | Y |  |
| - 1. Was selection of predictors based on univariable analysis avoided? | | Y |  |
| - 1. Were complexities in the data (e.g. censoring, competing risks, sampling of controls) accounted for appropriately? | | NI |  |
| - 1. Were relevant model performance measures evaluated appropriately? | | N |  |
| - 1. Were model overfitting and optimism in model performance accounted for? | | NI |  |
| - 1. Do predictors and their assigned weights in the final model correspond to the results from multivariable analysis? | | NI |  |
| **Risk of bias introduced by the analysis** | **RISK:**  *(low/ high/ unclear)* | **High** |  |
| *Rationale of bias rating:*  No information about event. Model performance measures were not evaluated. | | | |

**Step 4: Overall assessment**

| Use the following tables to reach overall judgements about risk of bias and concerns regarding applicability of the prediction model evaluation (development and/or validation) across all assessed domains.  *Complete for each evaluation of a distinct model.*   \| **Reaching an overall judgement about risk of bias of the prediction model evaluation** \| \| \| --- \| --- \| \| **Low risk of bias** \| If all domains were rated low risk of bias.  If a prediction model was developed without any external validation, and it was rated as low risk of bias for all domains, consider downgrading to **high risk of bias**. Such a model can only be considered as low risk of bias, if the development was based on a very large data set and included some form of internal validation. \| \| **High risk of bias** \| If at least one domain is judged to be at **high risk of bias**. \| \| **Unclear risk of bias** \| If an unclear risk of bias was noted in at least one domain and it was low risk for all other domains. \|  \| **Reaching an overall judgement about applicability of the prediction model evaluation** \| \| \| --- \| --- \| \| **Low concerns regarding applicability** \| If low concerns regarding applicability for all domains, the prediction model evaluation is judged to have **low concerns regarding applicability**. \| \| **High concerns regarding applicability** \| If high concerns regarding applicability for at least one domain, the prediction model evaluation is judged to have **high concerns regarding applicability**. \| \| **Unclear concerns regarding applicability** \| If unclear concerns (but no “high concern”) regarding applicability for at least one domain, the prediction model evaluation is judged to have **unclear concerns regarding applicability** overall. \| |
| --- | --- | --- | --- | --- | --- | --- | --- | --- | --- | --- | --- | --- | --- | --- | --- | --- |

| **Overall judgement about risk of bias and applicability of the prediction model evaluation** | | |
| --- | --- | --- |
| **Overall judgement of risk of bias** | **RISK:**  *(low/ high/ unclear)* | **High** |
| *Summary of sources of potential bias:*  No external validity. Cross-sectional approach to collect data. No longitudinal data. | | |
| **Overall judgement of applicability** | **CONCERN:**  *(low/ high/ unclear)* | **High** |
| *Summary of applicability concerns:*  Outcome *wasn’t defined.* | | |

**PROBAST – Second evaluator**

(Prediction model study Risk Of Bias Assessment Tool)

Published in Annals of Internal Medicine (freely available):

1. [PROBAST: A Tool to Assess the Risk of Bias and Applicability of Prediction Model Studies](https://annals.org/aim/fullarticle/2719961/probast-tool-assess-risk-bias-applicability-prediction-model-studies)
2. [PROBAST: A Tool to Assess Risk of Bias and Applicability of Prediction Model Studies: Explanation and Elaboration](https://annals.org/aim/fullarticle/2719962/probast-tool-assess-risk-bias-applicability-prediction-model-studies-explanation)

| **What does PROBAST assess?**  PROBAST assesses both the *risk of bias* and *concerns regarding applicability* of a study that evaluates (develops, validates or updates) a multivariable diagnostic or prognostic prediction model. It is designed to assess primary studies included in a systematic review.  *Bias* occurs if systematic flaws or limitations in the design, conduct or analysis of a primary study distort the results. For the purpose of prediction modelling studies, we have defined *risk of bias* to occur when shortcomings in the study design, conduct or analysis lead to systematically distorted estimates of a model’s predictive performance or to an inadequate model to address the research question. Model predictive performance is typically evaluated using calibration, discrimination and sometimes classification measures, and these are likely inaccurately estimated in studies with high risk of bias. *Applicability* refers to the extent to which the prediction model from the primary study matches your systematic review question, for example in terms of the participants, predictors or outcome of interest.  A primary study may include the development and/or validation or update of more than one prediction model. A PROBAST assessment should be completed for each distinct model that is developed, validated or updated (extended) for making individualised predictions. Where a publication assesses multiple prediction models, only complete a PROBAST assessment for those models that meet the inclusion criteria for your systematic review. Please note that subsequent use of the term “model” includes derivatives of models, such as simplified risk scores, nomograms, or recalibrations of models.  PROBAST is not designed for all multivariable diagnostic or prognostic studies. For example, studies using multivariable models to identify predictors associated with an outcome but not attempting to develop a model for making individualised predictions are not covered by PROBAST.  PROBAST includes four steps.   \| **Step** \| **Task** \| **When to complete** \| \| --- \| --- \| --- \| \| **1** \| Specify your systematic review question(s) \| Once per systematic review \| \| **2** \| Classify the type of prediction model evaluation \| Once for each model of interest in each publication being assessed, for each relevant outcome \| \| **3** \| Assess risk of bias and applicability \| Once for each development and validation of each distinct prediction model in a publication \| \| **4** \| Overall judgment \| Once for each development and validation of each distinct prediction model in a publication \|   If this is your first time using PROBAST, we strongly recommend reading the detailed explanation and elaboration (E&E, see link above) paper and to check the examples on www.probast.org |
| --- | --- | --- | --- | --- | --- | --- | --- | --- | --- | --- | --- | --- | --- | --- | --- |

**Step 1: Specify your systematic review question**

| State your systematic review question to facilitate the assessment of the applicability of the evaluated models to your question. *The following table should be completed once per systematic review.* |
| --- |

| **Criteria** | **Specify your systematic review question** |
| --- | --- |
| *Intended use of model:* | Machine learning tecniques for predicting risk of aggression/violent behaviour |
| ***Participants*** *including selection criteria and setting:* | Psychiatric patients in clinical and forensic settings |
| ***Predictors*** *(used in prediction modelling), including types of predictors (e.g. history, clinical examination, biochemical markers, imaging tests), time of measurement, specific measurement issues (e.g., any requirements/ prohibitions for specialized equipment):* | Clinical, sociodemographic, and historical characteristics.  No specific measurement issues. |
| *Outcome to be predicted:* | Patients with violent behaviours and patients with no violent  behaviour as an outcome |

**Step 2: Classify the type of prediction model evaluation**

| Use the following table to classify the evaluation as model development, model validation or model update, or combination. Different signalling questions apply for different types of prediction model evaluation. If the evaluation does not fit one of these classifications then PROBAST should not be used. |
| --- |

| **Classify the evaluation based on its aim** | | | |
| --- | --- | --- | --- |
| **Type of prediction study** | **PROBAST boxes to complete** | **Tick as appropriate** | **Definition for type of prediction model study** |
| Development only | Development | V | Prediction model development without external validation. These studies may include internal validation methods, such as bootstrapping and cross-validation techniques. |
| Development and validation | Development and validation | X | Prediction model development combined with external validation in other participants in the same article. |
| Validation only | Validation | x | External validation of existing (previously developed) model in other participants. |

| *This table should be completed once for each publication being assessed and for each relevant outcome in your review.* | |  |
| --- | --- | --- |
| **Publication reference** | Menger, V., Scheepers, F., & Spruit, M. (2018). Comparing deep learning and classical machine learning approaches for predicting inpatient violence incidents from clinical text. *Applied Sciences*, *8*(6), 981. | |
| **Models of interest** | RNN, CNN, NN, NB, SM, DT | |
| **Outcome of interest** | Predict for which admissions a violence incident will occur in the first 30 days, based on clinical texts that were written up to and including the first day of admission | |

**Step 3: Assess risk of bias and applicability**

| PROBAST is structured as four key domains. Each domain is judged for risk of bias (low, high or unclear) and includes signalling questions to help make judgements. Signalling questions are rated as yes (Y), probably yes (PY), probably no (PN), no (N) or no information (NI). All signalling questions are phrased so that “yes” indicates absence of bias. Any signalling question rated as “no” or “probably no” flags the potential for bias; you will need to use your judgement to determine whether the domain should be rated as “high”, “low” or “unclear” risk of bias. The guidance document contains further instructions and examples on rating signalling questions and risk of bias for each domain.  The first three domains are also rated for concerns regarding applicability (low/ high/ unclear) to your review question defined above.  *Complete all domains separately for each evaluation of a distinct model. Shaded boxes indicate where signalling questions do not apply and should not be answered.* |
| --- |

| **DOMAIN 1: Participants** | | | |
| --- | --- | --- | --- |
| **A. Risk of Bias** | | | |
| *Describe the sources of data and criteria for participant selection:*  A relevant dataset for the prediction task was obtained from the Psychiatry Department of the University Medical Center Utrecht (UMCU) in The Netherlands. This psychiatry department consist of six inpatient units, where patients with different medical histories are admitted, each with their own focus on different patient populations, diagnoses, and treatments.  Admissions from all six units between 2013 and 2016 were included in the dataset, resulting in a total of 2521 admissions from 1796 unique patients, including readmissions and transfers between different units. In all six units, mandatory reporting of violence incidents by one of the health care professionals involved in the incident took place. In the relevant time period, a total of 1267 violent incidents were reported. | | | |
|  | | Dev | Val |
| - 1. Were appropriate data sources used, e.g. cohort, RCT or nested case-control study data? | | PN |  |
| - 1. Were all inclusions and exclusions of participants appropriate? | | PY |  |
| **Risk of bias introduced by selection of participants** | **RISK:**  *(low/ high/ unclear)* | **High** |  |
| *Rationale of bias rating:* | | | |
| Data are collected for a clinical purpose other than development and validation of prediction models and are without a protocol. In routine care registries, data relating to inclusion and exclusion criteria are often inconsistently measured and recorded. | | | |
| **B. Applicability** | | | |
| *Describe included participants, setting and dates:*  See above | | | |
| **Concern that the included participants and setting do not match the review question** | **CONCERN:**  *(low/ high/ unclear)* | **Low** |  |
| *Rationale of applicability rating:* | | | |
| Included patients appear representative of the population specified in the review question. | | | |

| **DOMAIN 2: Predictors** | | | |
| --- | --- | --- | --- |
| **A. Risk of Bias** | | | |
| *List and describe predictors included in the final model, e.g. definition and timing of assessment:*  Clinical texts that were written up to and including the first day of admission. Since in many admissions, relevant information was not discovered and written down until the day of admission, especially in acute admissions, the prediction task did not include violence incidents on the day of admission. Although an number of incidents that was not negligible occurred on the day of admission, exploratory analysis showed that at that point in time, insufficient data was available to make a prediction. The interval of 30 days was furthermore chosen so that the prediction was specific, while the majority of incidents were included in the prediction, given that the mean duration of admission was 40.3 days. | | | |
|  | | Dev | Val |
| - 1. Were predictors defined and assessed in a similar way for all participants? | | PY |  |
| - 1. Were predictor assessments made without knowledge of outcome data? | | Y |  |
| - 1. Are all predictors available at the time the model is intended to be used? | | Y |  |
| **Risk of bias introduced by predictors or their assessment** | **RISK:**  *(low/ high/ unclear)* | **Low** |  |
| *Rationale of bias rating:*  We don’t be sure that the predictors were evaluated in the same way because different doctors and nurses wrote the various clinical notes. | | | |
| **B. Applicability** | | | |
| Concern that the definition, assessment or timing of predictors in the model do not match the review question | **CONCERN:**  *(low/ high/ unclear)* | **Low** |  |
| *Rationale of applicability rating:*  The definition, measurement and timing of the predictors matches the review question and targeted context | | | |

| **DOMAIN 3: Outcome** | | | |
| --- | --- | --- | --- |
| **A. Risk of Bias** | | | |
| *Describe the outcome, how it was defined and determined, and the time interval between predictor assessment and outcome determination:*  Incidents concerned violence from patients directed at staff or at other patients, including both verbal and physical aggression. In the relevant time period, a total of 1267 violent incidents were reported. After excluding incidents that did not involve a patient that was  admitted at the time of the incident (n = 19), for example incidents that involved visitors rather than the patient or incidents that happened after dismissal of a patient, a total of 1248 incidents remained. | | | |
|  | | Dev | Val |
| - 1. Was the outcome determined appropriately? | | Y |  |
| - 1. Was a pre-specified or standard outcome definition used? | | Y |  |
| - 1. Were predictors excluded from the outcome definition? | | Y |  |
| - 1. Was the outcome defined and determined in a similar way for all participants? | | Y |  |
| - 1. Was the outcome determined without knowledge of predictor information? | | Y |  |
| - 1. Was the time interval between predictor assessment and outcome determination appropriate? | | Y |  |
| **Risk of bias introduced by the outcome or its determination** | **RISK:**  *(low/ high/ unclear)* | **Low** |  |
| *Rationale of bias rating:*  All items of this domain were explicitly or implicitly addressed | | | |
| **B. Applicability** | | | |
| *At what time point was the outcome determined:*  Predictors and outcomes are assessed within a same time frame.  *If a composite outcome was used, describe the relative frequency/distribution of each contributing outcome:*  N/A | | | |
| **Concern that the outcome, its definition, timing or determination do not match the review question** | **CONCERN:**  *(low/ high/ unclear)* | **Low** |  |
| *Rationale of applicability rating:*  No concerns | | | |

| **DOMAIN 4: Analysis** | | | |
| --- | --- | --- | --- |
| **Risk of Bias** | | | |
| *Describe numbers of participants, number of candidate predictors, outcome events and events per candidate predictor:*  1796 unique patients; 1248 events | | | |
| *Describe how the model was developed (for example in regards to modelling technique (e.g. survival or logistic modelling), predictor selection, and risk group definition):*  As described in Section 2.2, the input data of the machine learning problem was a sequence of notes that is present in a patient’s EHR. We applied several models to the prediction task, which required a representation as described in Section 2.3 as input, either as a sequence or as a single representation. For each model we used a setup or architecture that is relatively straightforward and used in other literature. An overview of all models is presented in Table 3, along with the hyperparameters that are optimized. All other hyperparameters were fixed, for which a rationale is provided below. The most commonly used statistical models that are applied to text classification include Neural Networks, Bayesian Classifiers, Support Vector Machines, and Decision Trees [61,62]. Although pattern-based classifiers and k-nearest-neighbors type classifiers have also been applied to text classification, we did not apply them in this work because of their difficulties with imbalanced  datasets and difficulties with estimating probabilities that are needed for computing the AUC, respectively. For Neural Networks, we considered a three-layer feed-forward Neural Network as used, for example by Rajan et al. [63]. The Naive Bayes algorithm is very commonly used and has been shown to obtain good results, for example by Deshpande et al. [64], providing a good instantiation of a Bayesian Classifier. For Support Vector Machines, we considered a standard model with either a linear or radial kernel, which typically obtained the best results for text classification [65,66]. We used the CART algorithm finally to obtain a Decision Tree of which the depth and the number of features to consider when splitting could be controlled [67]. | | | |
| *Describe whether and how the model was validated, either internally (e.g. bootstrapping, cross validation, random split sample) or externally (e.g. temporal validation, geographical validation, different setting, different type of participants):*  5-fold cross validation strategy | | | |
| *Describe the performance measures of the model, e.g. (re)calibration, discrimination, (re)classification, net benefit, and whether they were adjusted for optimism:*  Discrimination(AUC); Deep Learning model (classification) | | | |
| *Describe any participants who were excluded from the analysis:*  After excluding incidents that did not involve a patient that was admitted at the time of the incident (n = 19), for example incidents that involved visitors rather than the patient or incidents that happened after dismissal of a patient, a total of 1248 incidents remained | | | |
| *Describe missing data on predictors and outcomes as well as methods used for missing data:*  *NA* | | | |
|  | | Dev | Val |
| - 1. Were there a reasonable number of participants with the outcome? | | Y |  |
| - 1. Were continuous and categorical predictors handled appropriately? | | Y |  |
| - 1. Were all enrolled participants included in the analysis? | | Y |  |
| - 1. Were participants with missing data handled appropriately? | | NI |  |
| - 1. Was selection of predictors based on univariable analysis avoided? | | Y |  |
| - 1. Were complexities in the data (e.g. censoring, competing risks, sampling of controls) accounted for appropriately? | | Y |  |
| - 1. Were relevant model performance measures evaluated appropriately? | | Y |  |
| - 1. Were model overfitting and optimism in model performance accounted for? | | Y |  |
| - 1. Do predictors and their assigned weights in the final model correspond to the results from multivariable analysis? | | NI |  |
| **Risk of bias introduced by the analysis** | **RISK:**  *(low/ high/ unclear)* | **Low** |  |
| *Rationale of bias rating:*  One possible limitation of the experimental evaluation was the 5-fold cross validation strategy for validating the model performances. Since several combinations of text representations, classification models and parameter settings have been evaluated, some degree of overfitting cannot be prevented. | | | |

**Step 4: Overall assessment**

| Use the following tables to reach overall judgements about risk of bias and concerns regarding applicability of the prediction model evaluation (development and/or validation) across all assessed domains.  *Complete for each evaluation of a distinct model.*   \| **Reaching an overall judgement about risk of bias of the prediction model evaluation** \| \| \| --- \| --- \| \| **Low risk of bias** \| If all domains were rated low risk of bias.  If a prediction model was developed without any external validation, and it was rated as low risk of bias for all domains, consider downgrading to **high risk of bias**. Such a model can only be considered as low risk of bias, if the development was based on a very large data set and included some form of internal validation. \| \| **High risk of bias** \| If at least one domain is judged to be at **high risk of bias**. \| \| **Unclear risk of bias** \| If an unclear risk of bias was noted in at least one domain and it was low risk for all other domains. \|  \| **Reaching an overall judgement about applicability of the prediction model evaluation** \| \| \| --- \| --- \| \| **Low concerns regarding applicability** \| If low concerns regarding applicability for all domains, the prediction model evaluation is judged to have **low concerns regarding applicability**. \| \| **High concerns regarding applicability** \| If high concerns regarding applicability for at least one domain, the prediction model evaluation is judged to have **high concerns regarding applicability**. \| \| **Unclear concerns regarding applicability** \| If unclear concerns (but no “high concern”) regarding applicability for at least one domain, the prediction model evaluation is judged to have **unclear concerns regarding applicability** overall. \| |
| --- | --- | --- | --- | --- | --- | --- | --- | --- | --- | --- | --- | --- | --- | --- | --- | --- |

| **Overall judgement about risk of bias and applicability of the prediction model evaluation** | | |
| --- | --- | --- |
| **Overall judgement of risk of bias** | **RISK:**  *(low/ high/ unclear)* | **High** |
| *Summary of sources of potential bias:*  Data are collected for a clinical purpose other than development and validation of prediction models and are without a protocol. In routine care registries, data relating to inclusion and exclusion criteria are often inconsistently measured and recorded. | | |
| **Overall judgement of applicability** | **CONCERN:**  *(low/ high/ unclear)* | **Low** |
| *Summary of applicability concerns:*  No concerns | | |

**PROBAST – Second evaluator**

(Prediction model study Risk Of Bias Assessment Tool)

Published in Annals of Internal Medicine (freely available):

1. [PROBAST: A Tool to Assess the Risk of Bias and Applicability of Prediction Model Studies](https://annals.org/aim/fullarticle/2719961/probast-tool-assess-risk-bias-applicability-prediction-model-studies)
2. [PROBAST: A Tool to Assess Risk of Bias and Applicability of Prediction Model Studies: Explanation and Elaboration](https://annals.org/aim/fullarticle/2719962/probast-tool-assess-risk-bias-applicability-prediction-model-studies-explanation)

| **What does PROBAST assess?**  PROBAST assesses both the *risk of bias* and *concerns regarding applicability* of a study that evaluates (develops, validates or updates) a multivariable diagnostic or prognostic prediction model. It is designed to assess primary studies included in a systematic review.  *Bias* occurs if systematic flaws or limitations in the design, conduct or analysis of a primary study distort the results. For the purpose of prediction modelling studies, we have defined *risk of bias* to occur when shortcomings in the study design, conduct or analysis lead to systematically distorted estimates of a model’s predictive performance or to an inadequate model to address the research question. Model predictive performance is typically evaluated using calibration, discrimination and sometimes classification measures, and these are likely inaccurately estimated in studies with high risk of bias. *Applicability* refers to the extent to which the prediction model from the primary study matches your systematic review question, for example in terms of the participants, predictors or outcome of interest.  A primary study may include the development and/or validation or update of more than one prediction model. A PROBAST assessment should be completed for each distinct model that is developed, validated or updated (extended) for making individualised predictions. Where a publication assesses multiple prediction models, only complete a PROBAST assessment for those models that meet the inclusion criteria for your systematic review. Please note that subsequent use of the term “model” includes derivatives of models, such as simplified risk scores, nomograms, or recalibrations of models.  PROBAST is not designed for all multivariable diagnostic or prognostic studies. For example, studies using multivariable models to identify predictors associated with an outcome but not attempting to develop a model for making individualised predictions are not covered by PROBAST.  PROBAST includes four steps.   \| **Step** \| **Task** \| **When to complete** \| \| --- \| --- \| --- \| \| **1** \| Specify your systematic review question(s) \| Once per systematic review \| \| **2** \| Classify the type of prediction model evaluation \| Once for each model of interest in each publication being assessed, for each relevant outcome \| \| **3** \| Assess risk of bias and applicability \| Once for each development and validation of each distinct prediction model in a publication \| \| **4** \| Overall judgment \| Once for each development and validation of each distinct prediction model in a publication \|   If this is your first time using PROBAST, we strongly recommend reading the detailed explanation and elaboration (E&E, see link above) paper and to check the examples on www.probast.org |
| --- | --- | --- | --- | --- | --- | --- | --- | --- | --- | --- | --- | --- | --- | --- | --- |

**Step 1: Specify your systematic review question**

| State your systematic review question to facilitate the assessment of the applicability of the evaluated models to your question. *The following table should be completed once per systematic review.* |
| --- |

| **Criteria** | **Specify your systematic review question** |
| --- | --- |
| *Intended use of model:* | Machine learning tecniques for predicting risk of aggression/violent behaviour |
| ***Participants*** *including selection criteria and setting:* | Psychiatric patients in clinical and forensic settings |
| ***Predictors*** *(used in prediction modelling), including types of predictors (e.g. history, clinical examination, biochemical markers, imaging tests), time of measurement, specific measurement issues (e.g., any requirements/ prohibitions for specialized equipment):* | Clinical, sociodemographic, and historical characteristics.  No specific measurement issues. |
| *Outcome to be predicted:* | Patients with violent behaviours and patients with no violent  behaviour as an outcome |

**Step 2: Classify the type of prediction model evaluation**

| Use the following table to classify the evaluation as model development, model validation or model update, or combination. Different signalling questions apply for different types of prediction model evaluation. If the evaluation does not fit one of these classifications then PROBAST should not be used. |
| --- |

| **Classify the evaluation based on its aim** | | | |
| --- | --- | --- | --- |
| **Type of prediction study** | **PROBAST boxes to complete** | **Tick as appropriate** | **Definition for type of prediction model study** |
| Development only | Development | X | Prediction model development without external validation. These studies may include internal validation methods, such as bootstrapping and cross-validation techniques. |
| Development and validation | Development and validation | V | Prediction model development combined with external validation in other participants in the same article. |
| Validation only | Validation | X | External validation of existing (previously developed) model in other participants. |

| *This table should be completed once for each publication being assessed and for each relevant outcome in your review.* | |  |
| --- | --- | --- |
| **Publication reference** | Menger, V., Spruit, M., van Est, R., Nap, E., & Scheepers, F. (2019). Machine Learning Approach to Inpatient Violence Risk Assessment Using Routinely Collected Clinical Notes in Electronic Health Records. *JAMA network open*, *2*(7), e196709. https://doi.org/10.1001/jamanetworkopen.2019.6709 | |
| **Models of interest** | Machine learning approach to perform VRA. | |
| **Outcome of interest** | Predictive validity and generalizability of prognostic models measured using area under the curve (AUC). | |

**Step 3: Assess risk of bias and applicability**

| PROBAST is structured as four key domains. Each domain is judged for risk of bias (low, high or unclear) and includes signalling questions to help make judgements. Signalling questions are rated as yes (Y), probably yes (PY), probably no (PN), no (N) or no information (NI). All signalling questions are phrased so that “yes” indicates absence of bias. Any signalling question rated as “no” or “probably no” flags the potential for bias; you will need to use your judgement to determine whether the domain should be rated as “high”, “low” or “unclear” risk of bias. The guidance document contains further instructions and examples on rating signalling questions and risk of bias for each domain.  The first three domains are also rated for concerns regarding applicability (low/ high/ unclear) to your review question defined above.  *Complete all domains separately for each evaluation of a distinct model. Shaded boxes indicate where signalling questions do not apply and should not be answered.* |
| --- |

| **DOMAIN 1: Participants** | | | |
| --- | --- | --- | --- |
| **A. Risk of Bias** | | | |
| In the study, the authors used data extracted from EHRs of 2 independent psychiatric treatment centers in the Netherlands. Data sources were not connected to each other or to sources outside the separate hospitals. The authors used deidentified data sets by deidentifying clinical notes using the Deindentification Method for Dutch Medical Text (DEDUCE) method.  Site 1, used for internal method validation, was the psychiatry department of the academic medical center in Utrecht, the Netherlands. All adults admitted between January 2013 and August 2018 were included. The author defined no exclusion criteria according to diagnosis, comorbidity, or other psychopathological conditions to maximize the translational value of predictive models. The resulting data set consisted of 3201 admissions of 2211 unique patients. Site 2, used for external method validation, was a general psychiatric hospital that delivers secondary care, with an additional focus on addiction care. All adults admitted between June 2016 and August 2018 were included in the data set. The resulting data set consisted of 3277 admissions of 1937 unique patients.  Notes that were written in the 4 weeks before admission up to the first 24 hours of admission were included in the data sets. Admissions with fewer than 100 words registered after 24 hours (12 admissions in site 1 and 24 admissions in site 2) were excluded from the data set. | | | |
|  | | Dev | Val |
| - 1. Were appropriate data sources used, e.g. cohort, RCT or nested case-control study data? | | PN | PN |
| - 1. Were all inclusions and exclusions of participants appropriate? | | PY | PY |
| **Risk of bias introduced by selection of participants** | **RISK:**  *(low/ high/ unclear)* | **High** | **High** |
| *Rationale of bias rating:* | | | |
| Data are collected for a clinical purpose other than development and validation of prediction models and are without a protocol. In routine care registries, data relating to inclusion and exclusion criteria are often inconsistently measured and recorded. | | | |
| **B. Applicability** | | | |
| The final data sets consisted of 3189 admissions from 2209 unique patients in site 1 and 3253 admissions from 1919 unique patients in site 2. Populations differed in age (mean [SD] age, 34.0 [16.6] and 45.9 [16.6] years, respectively), sex (1536 [48.2%] and 2097 [64.5%] men, respectively), and distribution of diagnoses. In both sites, the most commonly occurring diagnosis was schizophrenia or other psychotic disorders, followed by mood disorders and personality disorders in site 1 and substance-related disorders and bipolar disorders in site 2. | | | |
| **Concern that the included participants and setting do not match the review question** | **CONCERN:**  *(low/ high/ unclear)* | **Low** | **Low** |
| *Rationale of applicability rating:* | | | |
| No major issues identified. | | | |

| **DOMAIN 2: Predictors** | | | |
| --- | --- | --- | --- |
| **A. Risk of Bias** | | | |
| *List and describe predictors included in the final model, e.g. definition and timing of assessment:*  The data used to develop the prediction and validation model came from clinical notes. | | | |
|  | | Dev | Val |
| - 1. Were predictors defined and assessed in a similar way for all participants? | | PY | PY |
| - 1. Were predictor assessments made without knowledge of outcome data? | | Y | Y |
| - 1. Are all predictors available at the time the model is intended to be used? | | Y | Y |
| **Risk of bias introduced by predictors or their assessment** | **RISK:**  *(low/ high/ unclear)* | **Low** | **Low** |
| *Rationale of bias rating:*  All items of this domain were explicitly or implicitly addressed | | | |
| **B. Applicability** | | | |
| Concern that the definition, assessment or timing of predictors in the model do not match the review question | **CONCERN:**  *(low/ high/ unclear)* | **Low** | **Low** |
| *Rationale of applicability rating:*  No major issues | | | |

| **DOMAIN 3: Outcome** | | | |
| --- | --- | --- | --- |
| **A. Risk of Bias** | | | |
| *Describe the outcome, how it was defined and determined, and the time interval between predictor assessment and outcome determination:*  Reports of violent incidents were used to determine the outcome for each admission. In both sites mandatory reporting of all violent incidents takes place, including patient-staff and patient-patient violence. On the incident form, staff members who were involved in the incident were required to fill in structured information, a textual description of the incident, and incident severity as measured by the Staff Observation Aggression Scale–Revised.22 Our definition of a violent incident included all threatening and violent behavior of a verbal or physical nature directed at another person but excluded self-harm and inappropriate behavior, such as substance use, sexual intimidation, or vandalism. A positive outcome was defined as the presence of at least 1 incident in the first 4 weeks of admission, excluding the first 24 hours. No distinction in incident severity was made. | | | |
|  | | Dev | Val |
| - 1. Was the outcome determined appropriately? | | Y | Y |
| - 1. Was a pre-specified or standard outcome definition used? | | Y | Y |
| - 1. Were predictors excluded from the outcome definition? | | PY | PY |
| - 1. Was the outcome defined and determined in a similar way for all participants? | | Y | Y |
| - 1. Was the outcome determined without knowledge of predictor information? | | PY | PY |
| - 1. Was the time interval between predictor assessment and outcome determination appropriate? | | Y | Y |
| **Risk of bias introduced by the outcome or its determination** | **RISK:**  *(low/ high/ unclear)* | **Low** | **Low** |
| *Rationale of bias rating:*  All items of this domain were explicitly or implicitly addressed | | | |
| **B. Applicability** | | | |
| *At what time point was the outcome determined:*  Predictors and outcomes are assessed within a same time frame.  *If a composite outcome was used, describe the relative frequency/distribution of each contributing outcome:*  N/A | | | |
| **Concern that the outcome, its definition, timing or determination do not match the review question** | **CONCERN:**  *(low/ high/ unclear)* | **Low** | **Low** |
| *Rationale of applicability rating:*  No concerns | | | |

| **DOMAIN 4: Analysis** | | | |
| --- | --- | --- | --- |
| **Risk of Bias** | | | |
| *Describe numbers of participants, number of candidate predictors, outcome events and events per candidate predictor:*  Dev: Clinical notes recorded during a total of 3189 admissions of 2209 unique individuals at site 1  Val: 3253 admissions of 1919 unique individuals at site 2 | | | |
| *Describe how the model was developed (for example in regards to modelling technique (e.g. survival or logistic modelling), predictor selection, and risk group definition):*  To examine the potential predictive power hidden in clinical notes, the authors extracted the 1000 most frequent terms in the clinical notes, including bigrams, as binary variables.They then assessed the strength of each term’s association with the outcome using a χ2 test and computed Matthews correlation coefficients to obtain the direction of the association. They selected the top 10% of predictors on the basis of their χ2 scores in 1000 repeated samples with replacement, computing the fraction of times a term was included among the top predictors as a measure of within–data set generalizability of predictors. | | | |
| *Describe whether and how the model was validated, either internally (e.g. bootstrapping, cross validation, random split sample) or externally (e.g. temporal validation, geographical validation, different setting, different type of participants):*  The authors used nested cross validation procedure, to simultaneously optimize, train and assess the predictive validity of a model on a single dataset while obtaining a reliable estimate of performance without bias.  External validation of the machine learning approach was performed in site 2 by training a new model with equal experimental setup. To further elucidate model performance, the authors investigated predictive validity for earlyviolence vs late-violence and short-admission vs long-admission subgroups. Finally, trained models were exchanged between sites to test their generalizability | | | |
| *Describe the performance measures of the model, e.g. (re)calibration, discrimination, (re)classification, net benefit, and whether they were adjusted for optimism:*  The classification model consists of a Support Vector Machine with a radial kernel. Discrimination (AUC) | | | |
| *Describe any participants who were excluded from the analysis:*  Admissions in the developmental disorder ward were excluded according to patient age and the nature of violence 2 admissions in site 1 and 24 admissions in site 2 were excluded from the data set. | | | |
| *Describe missing data on predictors and outcomes as well as methods used for missing data:*  *NA* | | | |
|  | | Dev | Val |
| - 1. Were there a reasonable number of participants with the outcome? | | Y | Y |
| - 1. Were continuous and categorical predictors handled appropriately? | | PY | PY |
| - 1. Were all enrolled participants included in the analysis? | | PY | PY |
| - 1. Were participants with missing data handled appropriately? | | NI | NI |
| - 1. Was selection of predictors based on univariable analysis avoided? | | Y |  |
| - 1. Were complexities in the data (e.g. censoring, competing risks, sampling of controls) accounted for appropriately? | | PY | PY |
| - 1. Were relevant model performance measures evaluated appropriately? | | Y | Y |
| - 1. Were model overfitting and optimism in model performance accounted for? | | Y |  |
| - 1. Do predictors and their assigned weights in the final model correspond to the results from multivariable analysis? | | NI |  |
| **Risk of bias introduced by the analysis** | **RISK:**  *(low/ high/ unclear)* | **Low** | **Low** |
| *Rationale of bias rating:*  No major issues. | | | |

**Step 4: Overall assessment**

| Use the following tables to reach overall judgements about risk of bias and concerns regarding applicability of the prediction model evaluation (development and/or validation) across all assessed domains.  *Complete for each evaluation of a distinct model.*   \| **Reaching an overall judgement about risk of bias of the prediction model evaluation** \| \| \| --- \| --- \| \| **Low risk of bias** \| If all domains were rated low risk of bias.  If a prediction model was developed without any external validation, and it was rated as low risk of bias for all domains, consider downgrading to **high risk of bias**. Such a model can only be considered as low risk of bias, if the development was based on a very large data set and included some form of internal validation. \| \| **High risk of bias** \| If at least one domain is judged to be at **high risk of bias**. \| \| **Unclear risk of bias** \| If an unclear risk of bias was noted in at least one domain and it was low risk for all other domains. \|  \| **Reaching an overall judgement about applicability of the prediction model evaluation** \| \| \| --- \| --- \| \| **Low concerns regarding applicability** \| If low concerns regarding applicability for all domains, the prediction model evaluation is judged to have **low concerns regarding applicability**. \| \| **High concerns regarding applicability** \| If high concerns regarding applicability for at least one domain, the prediction model evaluation is judged to have **high concerns regarding applicability**. \| \| **Unclear concerns regarding applicability** \| If unclear concerns (but no “high concern”) regarding applicability for at least one domain, the prediction model evaluation is judged to have **unclear concerns regarding applicability** overall. \| |
| --- | --- | --- | --- | --- | --- | --- | --- | --- | --- | --- | --- | --- | --- | --- | --- | --- |

| **Overall judgement about risk of bias and applicability of the prediction model evaluation** | | |
| --- | --- | --- |
| **Overall judgement of risk of bias** | **RISK:**  *(low/ high/ unclear)* | **High** |
| *Summary of sources of potential bias:*  Data are collected for a clinical purpose other than development and validation of prediction models and are without a protocol. In routine care registries, data relating to inclusion and exclusion criteria are often inconsistently measured and recorded. | | |
| **Overall judgement of applicability** | **CONCERN:**  *(low/ high/ unclear)* | **Low** |
| *Summary of applicability concerns:*  No concerns | | |

**PROBAST**

(Prediction model study Risk Of Bias Assessment Tool)

Published in Annals of Internal Medicine (freely available):

1. [PROBAST: A Tool to Assess the Risk of Bias and Applicability of Prediction Model Studies](https://annals.org/aim/fullarticle/2719961/probast-tool-assess-risk-bias-applicability-prediction-model-studies)
2. [PROBAST: A Tool to Assess Risk of Bias and Applicability of Prediction Model Studies: Explanation and Elaboration](https://annals.org/aim/fullarticle/2719962/probast-tool-assess-risk-bias-applicability-prediction-model-studies-explanation)

| **What does PROBAST assess?**  PROBAST assesses both the *risk of bias* and *concerns regarding applicability* of a study that evaluates (develops, validates or updates) a multivariable diagnostic or prognostic prediction model. It is designed to assess primary studies included in a systematic review.  *Bias* occurs if systematic flaws or limitations in the design, conduct or analysis of a primary study distort the results. For the purpose of prediction modelling studies, we have defined *risk of bias* to occur when shortcomings in the study design, conduct or analysis lead to systematically distorted estimates of a model’s predictive performance or to an inadequate model to address the research question. Model predictive performance is typically evaluated using calibration, discrimination and sometimes classification measures, and these are likely inaccurately estimated in studies with high risk of bias. *Applicability* refers to the extent to which the prediction model from the primary study matches your systematic review question, for example in terms of the participants, predictors or outcome of interest.  A primary study may include the development and/or validation or update of more than one prediction model. A PROBAST assessment should be completed for each distinct model that is developed, validated or updated (extended) for making individualised predictions. Where a publication assesses multiple prediction models, only complete a PROBAST assessment for those models that meet the inclusion criteria for your systematic review. Please note that subsequent use of the term “model” includes derivatives of models, such as simplified risk scores, nomograms, or recalibrations of models.  PROBAST is not designed for all multivariable diagnostic or prognostic studies. For example, studies using multivariable models to identify predictors associated with an outcome but not attempting to develop a model for making individualised predictions are not covered by PROBAST.  PROBAST includes four steps.   \| **Step** \| **Task** \| **When to complete** \| \| --- \| --- \| --- \| \| **1** \| Specify your systematic review question(s) \| Once per systematic review \| \| **2** \| Classify the type of prediction model evaluation \| Once for each model of interest in each publication being assessed, for each relevant outcome \| \| **3** \| Assess risk of bias and applicability \| Once for each development and validation of each distinct prediction model in a publication \| \| **4** \| Overall judgment \| Once for each development and validation of each distinct prediction model in a publication \|   If this is your first time using PROBAST, we strongly recommend reading the detailed explanation and elaboration (E&E, see link above) paper and to check the examples on www.probast.org |
| --- | --- | --- | --- | --- | --- | --- | --- | --- | --- | --- | --- | --- | --- | --- | --- |

**Step 1: Specify your systematic review question**

| State your systematic review question to facilitate the assessment of the applicability of the evaluated models to your question. *The following table should be completed once per systematic review.* |
| --- |

| **Criteria** | **Specify your systematic review question** |
| --- | --- |
| *Intended use of model:* | Machine learning tecniques for predicting risk of aggression/violent behaviour |
| ***Participants*** *including selection criteria and setting:* | Psychiatric patients in clinical and forensic settings |
| ***Predictors*** *(used in prediction modelling), including types of predictors (e.g. history, clinical examination, biochemical markers, imaging tests), time of measurement, specific measurement issues (e.g., any requirements/ prohibitions for specialized equipment):* | Clinical, sociodemographic, and historical characteristics.  No specific measurement issues. |
| *Outcome to be predicted:* | Patients with violent behaviours and patients with no violent  behaviour as an outcome |

**Step 2: Classify the type of prediction model evaluation**

| Use the following table to classify the evaluation as model development, model validation or model update, or combination. Different signalling questions apply for different types of prediction model evaluation. If the evaluation does not fit one of these classifications then PROBAST should not be used. |
| --- |

| **Classify the evaluation based on its aim** | | | |
| --- | --- | --- | --- |
| **Type of prediction study** | **PROBAST boxes to complete** | **Tick as appropriate** | **Definition for type of prediction model study** |
| Development only | Development | v | Prediction model development without external validation. These studies may include internal validation methods, such as bootstrapping and cross-validation techniques. |
| Development and validation | Development and validation | X | Prediction model development combined with external validation in other participants in the same article. |
| Validation only | Validation | X | External validation of existing (previously developed) model in other participants. |

| *This table should be completed once for each publication being assessed and for each relevant outcome in your review.* | |  |
| --- | --- | --- |
| **Publication reference** | Suchting, R., Green, C. E., Glazier, S. M., & Lane, S. D. (2018). A data science approach to predicting patient aggressive events in a psychiatric hospital. *Psychiatry research*, *268*, 217-222. | |
| **Models of interest** | GLM, RF, GBM, DNN | |
| **Outcome of interest** | The primary outcome of interest was the presence (1.4%) versus absence (98.6%) of an aggressive event toward staff or patients. | |

**Step 3: Assess risk of bias and applicability**

| PROBAST is structured as four key domains. Each domain is judged for risk of bias (low, high or unclear) and includes signalling questions to help make judgements. Signalling questions are rated as yes (Y), probably yes (PY), probably no (PN), no (N) or no information (NI). All signalling questions are phrased so that “yes” indicates absence of bias. Any signalling question rated as “no” or “probably no” flags the potential for bias; you will need to use your judgement to determine whether the domain should be rated as “high”, “low” or “unclear” risk of bias. The guidance document contains further instructions and examples on rating signalling questions and risk of bias for each domain.  The first three domains are also rated for concerns regarding applicability (low/ high/ unclear) to your review question defined above.  *Complete all domains separately for each evaluation of a distinct model. Shaded boxes indicate where signalling questions do not apply and should not be answered.* |
| --- |

| **DOMAIN 1: Participants** | | | |
| --- | --- | --- | --- |
| **A. Risk of Bias** | | | |
| *Describe the sources of data and criteria for participant selection:*  This retrospective study utilized electronic health records (N = 29,841) collected between January 2010 and December 2015 at Harris County Psychiatric Center, a 274- bed safety net community psychiatric facility | | | |
|  | | Dev | Val |
| - 1. Were appropriate data sources used, e.g. cohort, RCT or nested case-control study data? | | PN |  |
| - 1. Were all inclusions and exclusions of participants appropriate? | | PY |  |
| **Risk of bias introduced by selection of participants** | **RISK:**  *(low/ high/ unclear)* | **High** |  |
| *Rationale of bias rating:* | | | |
| Data are collected for a clinical purpose other than development and validation of prediction models and are without a protocol. In routine care registries, data relating to inclusion and exclusion criteria are often inconsistently measured and recorded. | | | |
| **B. Applicability** | | | |
| *Describe included participants, setting and dates:*  The hospital, based in a large urban setting with an average of >9000 admissions annually, representative of public inpatient psychiatric hospitals in many large U.S. metropolitan areas (geographic differences in diversity not withstanding). Patient  admission to HCPC occurred across a range of varying conditions (i.e., psychiatric symptom presentation, voluntary / involuntary status, time of day, referral sources). Data were not differentially included or excluded based on any specific diagnostic or admission criteria. Patients provided information as part of standardized hospital admitting and assessment procedures, summarized below. | | | |
| **Concern that the included participants and setting do not match the review question** | **CONCERN:**  *(low/ high/ unclear)* | **Low** |  |
| *Rationale of applicability rating:* | | | |
| The present dataset is that it was derived from a single inpatient facility best characterized as a large, urban safety-net community mental health hospital. | | | |

| **DOMAIN 2: Predictors** | | | |
| --- | --- | --- | --- |
| **A. Risk of Bias** | | | |
| *List and describe predictors included in the final model, e.g. definition and timing of assessment:*  This examination included a full demographic profile, patient vitals (i.e., height, weight, blood pressure), and a comprehensive psychosocial assessment, including histories of early development, education, military service, vocation/work, medical status, psychiatric status, drug/substance use and treatment, nicotine/tobacco use and counseling, abuse (victim or perpetrated physical/verbal/emotional/ sexual abuse), legal status, marital status, religious beliefs, financial status, and living situation. A nursing assessment collected information regarding sleep habits, pain status, patient behavior during interview, a risk assessment, and evaluation of patient mood (via the Affective Disorders Rating Scale (ADRS; Swann et al., 2004). The initial psychiatric evaluation/mental status exam assessed general appearance (i.e., hygiene), musculoskeletal system, speech pattern, thought pro- cesses and content, perception, depression, affect, insight, judgment, skin integrity, head trauma, suicidal/homicidal/assault ideation, deterioration in function, chemical dependency, hallucinations, and delusions.  From these initial assessments, the final set of 328 predictor variables was derived. Names, descriptions, and (where applicable) standardized instruments used to obtain the 328 predictor variables. | | | |
|  | | Dev | Val |
| - 1. Were predictors defined and assessed in a similar way for all participants? | | Y |  |
| - 1. Were predictor assessments made without knowledge of outcome data? | | PY |  |
| - 1. Are all predictors available at the time the model is intended to be used? | | Y |  |
| **Risk of bias introduced by predictors or their assessment** | **RISK:**  *(low/ high/ unclear)* | **Low** |  |
| *Rationale of bias rating:*  All items of this domain were explicitly or implicitly addressed | | | |
| **B. Applicability** | | | |
| Concern that the definition, assessment or timing of predictors in the model do not match the review question | **CONCERN:**  *(low/ high/ unclear)* | **Low** |  |
| *Rationale of applicability rating:*  No concerns | | | |

| **DOMAIN 3: Outcome** | | | |
| --- | --- | --- | --- |
| **A. Risk of Bias** | | | |
| *Describe the outcome, how it was defined and determined, and the time interval between predictor assessment and outcome determination:*  Outcome measure (positive/negative for an aggressive incident). An aggressive event is coded into the hospital medical record following any episode of uncontrolled verbal or physical aggression that required intervention by and assistance from additional hospital staff to manage the event. In cases of verbal aggression, such intervention would indicate that physical aggression was deemed imminent by staff. All episodes of aggression are mandatorily recorded by hospital staff and coded in the EMR as patient-on-patient or patient-on-staff; both types were included in the data analyses. All aggressive events included codes for the type of action taken in response to the aggressive incident (e.g., “Transferred to:”, “Family notified”, “Plan of care revised”, “Education/ Training”, etc.). Type of action taken in response to the incident was not included in the data analyses | | | |
|  | | Dev | Val |
| - 1. Was the outcome determined appropriately? | | Y |  |
| - 1. Was a pre-specified or standard outcome definition used? | | Y |  |
| - 1. Were predictors excluded from the outcome definition? | | PY |  |
| - 1. Was the outcome defined and determined in a similar way for all participants? | | Y |  |
| - 1. Was the outcome determined without knowledge of predictor information? | | PY |  |
| - 1. Was the time interval between predictor assessment and outcome determination appropriate? | | Y |  |
| **Risk of bias introduced by the outcome or its determination** | **RISK:**  *(low/ high/ unclear)* | **Low** |  |
| *Rationale of bias rating:*  All items of this domain were explicitly or implicitly addressed | | | |
| **B. Applicability** | | | |
| *At what time point was the outcome determined:*  Predictors and outcomes are assessed within a same time frame.  *If a composite outcome was used, describe the relative frequency/distribution of each contributing outcome:*  N/A | | | |
| **Concern that the outcome, its definition, timing or determination do not match the review question** | **CONCERN:**  *(low/ high/ unclear)* | **Low** |  |
| *Rationale of applicability rating:*  No concerns | | | |

| **DOMAIN 4: Analysis** | | | |
| --- | --- | --- | --- |
| **Risk of Bias** | | | |
| *Describe numbers of participants, number of candidate predictors, outcome events and events per candidate predictor:*  29,841 electronic health records; 328 predictors (313 categorical, 15 continuous) and one outcome measure (positive/negative for an aggressive incident).; outcome of interest was the presence (1.4%) versus absence (98.6%) of an aggressive event toward staff or patients. | | | |
| *Describe how the model was developed (for example in regards to modelling technique (e.g. survival or logistic modelling), predictor selection, and risk group definition):*  Four machine learning algorithms were utilized to provide probabilities of an aggressive incident during the inpatient stay: penalized generalized linear modeling (GLM), random forest (RF), gradient boosting machine (GBM), and deep neural network (DNN). | | | |
| *Describe whether and how the model was validated, either internally (e.g. bootstrapping, cross validation, random split sample) or externally (e.g. temporal validation, geographical validation, different setting, different type of participants):*  5-fold cross validation | | | |
| *Describe the performance measures of the model, e.g. (re)calibration, discrimination, (re)classification, net benefit, and whether they were adjusted for optimism:*  *Discrimination (AUC)* | | | |
| *Describe any participants who were excluded from the analysis:*  *NA* | | | |
| *Describe missing data on predictors and outcomes as well as methods used for missing data:*  Data were missing in approximately 10% of observations, wYith the majority of missingness occurring in the categorical predictors. Assuming that missingness is itself a potentially important factor, missingness in each categorical predictor was addressed through the creation of a new categorical level to indicate “missing.” Remaining missing data (less than 1%) in the continuous predictors were handled natively by each machine learning algorithm (e.g., imputation, data partitioning). | | | |
|  | | Dev | Val |
| - 1. Were there a reasonable number of participants with the outcome? | | Y |  |
| - 1. Were continuous and categorical predictors handled appropriately? | | Y |  |
| - 1. Were all enrolled participants included in the analysis? | | Y |  |
| - 1. Were participants with missing data handled appropriately? | | Y |  |
| - 1. Was selection of predictors based on univariable analysis avoided? | | Y |  |
| - 1. Were complexities in the data (e.g. censoring, competing risks, sampling of controls) accounted for appropriately? | | Y |  |
| - 1. Were relevant model performance measures evaluated appropriately? | | Y |  |
| - 1. Were model overfitting and optimism in model performance accounted for? | | NI |  |
| - 1. Do predictors and their assigned weights in the final model correspond to the results from multivariable analysis? | | NI |  |
| **Risk of bias introduced by the analysis** | **RISK:**  *(low/ high/ unclear)* | **Low** |  |
| *Rationale of bias rating:*  No concerns | | | |

**Step 4: Overall assessment**

| Use the following tables to reach overall judgements about risk of bias and concerns regarding applicability of the prediction model evaluation (development and/or validation) across all assessed domains.  *Complete for each evaluation of a distinct model.*   \| **Reaching an overall judgement about risk of bias of the prediction model evaluation** \| \| \| --- \| --- \| \| **Low risk of bias** \| If all domains were rated low risk of bias.  If a prediction model was developed without any external validation, and it was rated as low risk of bias for all domains, consider downgrading to **high risk of bias**. Such a model can only be considered as low risk of bias, if the development was based on a very large data set and included some form of internal validation. \| \| **High risk of bias** \| If at least one domain is judged to be at **high risk of bias**. \| \| **Unclear risk of bias** \| If an unclear risk of bias was noted in at least one domain and it was low risk for all other domains. \|  \| **Reaching an overall judgement about applicability of the prediction model evaluation** \| \| \| --- \| --- \| \| **Low concerns regarding applicability** \| If low concerns regarding applicability for all domains, the prediction model evaluation is judged to have **low concerns regarding applicability**. \| \| **High concerns regarding applicability** \| If high concerns regarding applicability for at least one domain, the prediction model evaluation is judged to have **high concerns regarding applicability**. \| \| **Unclear concerns regarding applicability** \| If unclear concerns (but no “high concern”) regarding applicability for at least one domain, the prediction model evaluation is judged to have **unclear concerns regarding applicability** overall. \| |
| --- | --- | --- | --- | --- | --- | --- | --- | --- | --- | --- | --- | --- | --- | --- | --- | --- |

| **Overall judgement about risk of bias and applicability of the prediction model evaluation** | | |
| --- | --- | --- |
| **Overall judgement of risk of bias** | **RISK:**  *(low/ high/ unclear)* | **High** |
| *Summary of sources of potential bias:*  Data are collected for a clinical purpose other than development and validation of prediction models and are without a protocol. In routine care registries, data relating to inclusion and exclusion criteria are often inconsistently measured and recorded. | | |
| **Overall judgement of applicability** | **CONCERN:**  *(low/ high/ unclear)* | **Low** |
| *Summary of applicability concerns:*  No concerns | | |

**PROBAST**

(Prediction model study Risk Of Bias Assessment Tool)

Published in Annals of Internal Medicine (freely available):

1. [PROBAST: A Tool to Assess the Risk of Bias and Applicability of Prediction Model Studies](https://annals.org/aim/fullarticle/2719961/probast-tool-assess-risk-bias-applicability-prediction-model-studies)
2. [PROBAST: A Tool to Assess Risk of Bias and Applicability of Prediction Model Studies: Explanation and Elaboration](https://annals.org/aim/fullarticle/2719962/probast-tool-assess-risk-bias-applicability-prediction-model-studies-explanation)

| **What does PROBAST assess?**  PROBAST assesses both the *risk of bias* and *concerns regarding applicability* of a study that evaluates (develops, validates or updates) a multivariable diagnostic or prognostic prediction model. It is designed to assess primary studies included in a systematic review.  *Bias* occurs if systematic flaws or limitations in the design, conduct or analysis of a primary study distort the results. For the purpose of prediction modelling studies, we have defined *risk of bias* to occur when shortcomings in the study design, conduct or analysis lead to systematically distorted estimates of a model’s predictive performance or to an inadequate model to address the research question. Model predictive performance is typically evaluated using calibration, discrimination and sometimes classification measures, and these are likely inaccurately estimated in studies with high risk of bias. *Applicability* refers to the extent to which the prediction model from the primary study matches your systematic review question, for example in terms of the participants, predictors or outcome of interest.  A primary study may include the development and/or validation or update of more than one prediction model. A PROBAST assessment should be completed for each distinct model that is developed, validated or updated (extended) for making individualised predictions. Where a publication assesses multiple prediction models, only complete a PROBAST assessment for those models that meet the inclusion criteria for your systematic review. Please note that subsequent use of the term “model” includes derivatives of models, such as simplified risk scores, nomograms, or recalibrations of models.  PROBAST is not designed for all multivariable diagnostic or prognostic studies. For example, studies using multivariable models to identify predictors associated with an outcome but not attempting to develop a model for making individualised predictions are not covered by PROBAST.  PROBAST includes four steps.   \| **Step** \| **Task** \| **When to complete** \| \| --- \| --- \| --- \| \| **1** \| Specify your systematic review question(s) \| Once per systematic review \| \| **2** \| Classify the type of prediction model evaluation \| Once for each model of interest in each publication being assessed, for each relevant outcome \| \| **3** \| Assess risk of bias and applicability \| Once for each development and validation of each distinct prediction model in a publication \| \| **4** \| Overall judgment \| Once for each development and validation of each distinct prediction model in a publication \|   If this is your first time using PROBAST, we strongly recommend reading the detailed explanation and elaboration (E&E, see link above) paper and to check the examples on www.probast.org |
| --- | --- | --- | --- | --- | --- | --- | --- | --- | --- | --- | --- | --- | --- | --- | --- |

**Step 1: Specify your systematic review question**

| State your systematic review question to facilitate the assessment of the applicability of the evaluated models to your question. *The following table should be completed once per systematic review.* |
| --- |

| **Criteria** | **Specify your systematic review question** |
| --- | --- |
| *Intended use of model:* | Machine learning tecniques for predicting risk of aggression/violent behaviour |
| ***Participants*** *including selection criteria and setting:* | Psychiatric patients in clinical and forensic settings |
| ***Predictors*** *(used in prediction modelling), including types of predictors (e.g. history, clinical examination, biochemical markers, imaging tests), time of measurement, specific measurement issues (e.g., any requirements/ prohibitions for specialized equipment):* | Clinical, sociodemographic, and historical characteristics.  No specific measurement issues. |
| *Outcome to be predicted:* | Patients with violent behaviours and patients with no violent  behaviour as an outcome |

**Step 2: Classify the type of prediction model evaluation**

| Use the following table to classify the evaluation as model development, model validation or model update, or combination. Different signalling questions apply for different types of prediction model evaluation. If the evaluation does not fit one of these classifications then PROBAST should not be used. |
| --- |

| **Classify the evaluation based on its aim** | | | |
| --- | --- | --- | --- |
| **Type of prediction study** | **PROBAST boxes to complete** | **Tick as appropriate** | **Definition for type of prediction model study** |
| Development only | Development | V | Prediction model development without external validation. These studies may include internal validation methods, such as bootstrapping and cross-validation techniques. |
| Development and validation | Development and validation | X | Prediction model development combined with external validation in other participants in the same article. |
| Validation only | Validation | x | External validation of existing (previously developed) model in other participants. |

| *This table should be completed once for each publication being assessed and for each relevant outcome in your review.* | |  |
| --- | --- | --- |
| **Publication reference** | Wang, K. Z., Bani-Fatemi, A., Adanty, C., Harripaul, R., Griffiths, J., Kolla, N., ... & De Luca, V. (2020). Prediction of physical violence in schizophrenia with machine learning algorithms. *Psychiatry research*, *289*, 112960. | |
| **Models of interest** | LASSO, elastic net, random forest, GBRT, SVM, SVM with RBF kernels | |
| **Outcome of interest** | Presence of violence | |

**Step 3: Assess risk of bias and applicability**

| PROBAST is structured as four key domains. Each domain is judged for risk of bias (low, high or unclear) and includes signalling questions to help make judgements. Signalling questions are rated as yes (Y), probably yes (PY), probably no (PN), no (N) or no information (NI). All signalling questions are phrased so that “yes” indicates absence of bias. Any signalling question rated as “no” or “probably no” flags the potential for bias; you will need to use your judgement to determine whether the domain should be rated as “high”, “low” or “unclear” risk of bias. The guidance document contains further instructions and examples on rating signalling questions and risk of bias for each domain.  The first three domains are also rated for concerns regarding applicability (low/ high/ unclear) to your review question defined above.  *Complete all domains separately for each evaluation of a distinct model. Shaded boxes indicate where signalling questions do not apply and should not be answered.* |
| --- |

| **DOMAIN 1: Participants** | | | |
| --- | --- | --- | --- |
| **A. Risk of Bias** | | | |
| *Describe the sources of data and criteria for participant selection:*  275 participants were recruited from the Centre for Addiction and Mental Health (CAMH), located in Toronto, Canada. All study participants met the inclusion criteria of being between the ages of 18 and 75; and the diagnosis of schizophrenia spectrum disorder was confirmed by the Structured Clinical Interview for  the DSM-IV (SCID-I/P). Exclusion criteria included having any diagnosis of an intellectual disability, major neurological disorder, or substance- induced psychosis; or having a history of head trauma resulting  in loss of consciousness. All participants provided written informed consent for study participation and disclosure of personal health records for electronic medical chart verification.  The presence of violence was determined retrospectively through a review of all available patient electronic medical records for any documentation of incidences of physically violent behavior. | | | |
|  | | Dev | Val |
| - 1. Were appropriate data sources used, e.g. cohort, RCT or nested case-control study data? | | PY |  |
| - 1. Were all inclusions and exclusions of participants appropriate? | | Y |  |
| **Risk of bias introduced by selection of participants** | **RISK:**  *(low/ high/ unclear)* | **Low** |  |
| *Rationale of bias rating:* | | | |
| Exclusions appear appropriate | | | |
| **B. Applicability** | | | |
| *Describe included participants, setting and dates:*  In final study sample of 275 patients with schizophrenia, there were 103 (37%) who had histories of violence and 172 (63%) without any violent tendencies. Clinical setting. | | | |
| **Concern that the included participants and setting do not match the review question** | **CONCERN:**  *(low/ high/ unclear)* | **High** |  |
| *Rationale of applicability rating:* | | | |
| Small sample size; the dataset was derived from a single inpatient facility best characterized as a large, urban safety-net community mental health hospital. Thus, the present findings may be limited in generalizability to other regions or mental health settings | | | |

| **DOMAIN 2: Predictors** | | | |
| --- | --- | --- | --- |
| **A. Risk of Bias** | | | |
| *List and describe predictors included in the final model, e.g. definition and timing of assessment:*  28 predictive variables based on previously identified factors associated with violence or schizophrenia. These variables were obtained by administering cross-sectional assessments in the form of structured interviews and self-report questionnaires and utilizing psychiatric rating scales and questions assessing medical history and demographic information. | | | |
|  | | Dev | Val |
| - 1. Were predictors defined and assessed in a similar way for all participants? | | Y |  |
| - 1. Were predictor assessments made without knowledge of outcome data? | | PY |  |
| - 1. Are all predictors available at the time the model is intended to be used? | | Y |  |
| **Risk of bias introduced by predictors or their assessment** | **RISK:**  *(low/ high/ unclear)* | **Low** |  |
| *Rationale of bias rating:*  The items (predictors) were assessed similarly for all study subjects, as it was implemented in the routine care procedures. | | | |
| **B. Applicability** | | | |
| Concern that the definition, assessment or timing of predictors in the model do not match the review question | **CONCERN:**  *(low/ high/ unclear)* | **Low** |  |
| *Rationale of applicability rating:*  Predictors were assessed at presentation and defined in a standard way. They appear to match the review question. | | | |

| **DOMAIN 3: Outcome** | | | |
| --- | --- | --- | --- |
| **A. Risk of Bias** | | | |
| *Describe the outcome, how it was defined and determined, and the time interval between predictor assessment and outcome determination:*  Violence status is the outcome variable. The presence of violence was determined retrospectively through a  review of all available patient electronic medical records for any documentation of incidences of physically violent behavior. The authors assigned severity scores for each participant ranging from an absence of physical violence to assault resulting in serious bodily injury, as outlined in the Modified Overt Aggression Scale (MOAS). | | | |
|  | | Dev | Val |
| - 1. Was the outcome determined appropriately? | | NI |  |
| - 1. Was a pre-specified or standard outcome definition used? | | NI |  |
| - 1. Were predictors excluded from the outcome definition? | | NI |  |
| - 1. Was the outcome defined and determined in a similar way for all participants? | | Y |  |
| - 1. Was the outcome determined without knowledge of predictor information? | | PN |  |
| - 1. Was the time interval between predictor assessment and outcome determination appropriate? | | PN |  |
| **Risk of bias introduced by the outcome or its determination** | **RISK:**  *(low/ high/ unclear)* | **unclear** |  |
| *Rationale of bias rating:*  It’s not clear the criteria to divide the patients in violent/non violent | | | |
| **B. Applicability** | | | |
| *At what time point was the outcome determined:*  Outcome (violence/no violence) was determined before the measurement of the predictors which is in line with the diagnostic nature of the study.  *If a composite outcome was used, describe the relative frequency/distribution of each contributing outcome:*  N/A | | | |
| **Concern that the outcome, its definition, timing or determination do not match the review question** | **CONCERN:**  *(low/ high/ unclear)* | **Low** |  |
| *Rationale of applicability rating:*  The outcome is the same as that specified in the review question. | | | |

| **DOMAIN 4: Analysis** | | | |
| --- | --- | --- | --- |
| **Risk of Bias** | | | |
| *Describe numbers of participants, number of candidate predictors, outcome events and events per candidate predictor:*  *275 patients with schizophrenia, 28 candidate predictors, 103 events* | | | |
| *Describe how the model was developed (for example in regards to modelling technique (e.g. survival or logistic modelling), predictor selection, and risk group definition):*  Six classification algorithms were performed for model prediction comparison: the least absolute shrinkage and selection operator (lasso), elastic net, random forest, gradient boosted regression trees (GBRT), support vector machine (SVM) classifier, and SVM classifiers with radial basis function (RBF) kernels. | | | |
| *Describe whether and how the model was validated, either internally (e.g. bootstrapping, cross validation, random split sample) or externally (e.g. temporal validation, geographical validation, different setting, different type of participants):*  Stratified k-fold cross validation technique, assigning k = 5. | | | |
| *Describe the performance measures of the model, e.g. (re)calibration, discrimination, (re)classification, net benefit, and whether they were adjusted for optimism:*  Accuracy, AUROC, sensitivity, specificity, PPV, and NPV | | | |
| *Describe any participants who were excluded from the analysis:*  *NA* | | | |
| *Describe missing data on predictors and outcomes as well as methods used for missing data:*  *NA* | | | |
|  | | Dev | Val |
| - 1. Were there a reasonable number of participants with the outcome? | | Y |  |
| - 1. Were continuous and categorical predictors handled appropriately? | | Y |  |
| - 1. Were all enrolled participants included in the analysis? | | Y |  |
| - 1. Were participants with missing data handled appropriately? | | NI |  |
| - 1. Was selection of predictors based on univariable analysis avoided? | | Y |  |
| - 1. Were complexities in the data (e.g. censoring, competing risks, sampling of controls) accounted for appropriately? | | NI |  |
| - 1. Were relevant model performance measures evaluated appropriately? | | Y |  |
| - 1. Were model overfitting and optimism in model performance accounted for? | | Y |  |
| - 1. Do predictors and their assigned weights in the final model correspond to the results from multivariable analysis? | | NI |  |
| **Risk of bias introduced by the analysis** | **RISK:**  *(low/ high/ unclear)* | **Unclear** |  |
| *Rationale of bias rating:*  Small sample size to train and validate the classification models. | | | |

**Step 4: Overall assessment**

| Use the following tables to reach overall judgements about risk of bias and concerns regarding applicability of the prediction model evaluation (development and/or validation) across all assessed domains.  *Complete for each evaluation of a distinct model.*   \| **Reaching an overall judgement about risk of bias of the prediction model evaluation** \| \| \| --- \| --- \| \| **Low risk of bias** \| If all domains were rated low risk of bias.  If a prediction model was developed without any external validation, and it was rated as low risk of bias for all domains, consider downgrading to **high risk of bias**. Such a model can only be considered as low risk of bias, if the development was based on a very large data set and included some form of internal validation. \| \| **High risk of bias** \| If at least one domain is judged to be at **high risk of bias**. \| \| **Unclear risk of bias** \| If an unclear risk of bias was noted in at least one domain and it was low risk for all other domains. \|  \| **Reaching an overall judgement about applicability of the prediction model evaluation** \| \| \| --- \| --- \| \| **Low concerns regarding applicability** \| If low concerns regarding applicability for all domains, the prediction model evaluation is judged to have **low concerns regarding applicability**. \| \| **High concerns regarding applicability** \| If high concerns regarding applicability for at least one domain, the prediction model evaluation is judged to have **high concerns regarding applicability**. \| \| **Unclear concerns regarding applicability** \| If unclear concerns (but no “high concern”) regarding applicability for at least one domain, the prediction model evaluation is judged to have **unclear concerns regarding applicability** overall. \| |
| --- | --- | --- | --- | --- | --- | --- | --- | --- | --- | --- | --- | --- | --- | --- | --- | --- |

| **Overall judgement about risk of bias and applicability of the prediction model evaluation** | | |
| --- | --- | --- |
| **Overall judgement of risk of bias** | **RISK:**  *(low/ high/ unclear)* | **High** |
| *Summary of sources of potential bias:*  No external validity, small size, missing data weren’t handled appropriately. | | |
| **Overall judgement of applicability** | **CONCERN:**  *(low/ high/ unclear)* | **High** |
| *Summary of applicability concerns:*  Small sample size: the dataset was derived from a single inpatient facility best characterized as a large, urban safety-net community mental health hospital. Thus, the present findings may be limited in generalizability to other regions or mental health settings. | | |

**PROBAST – Second evaluator**

(Prediction model study Risk Of Bias Assessment Tool)

Published in Annals of Internal Medicine (freely available):

1. [PROBAST: A Tool to Assess the Risk of Bias and Applicability of Prediction Model Studies](https://annals.org/aim/fullarticle/2719961/probast-tool-assess-risk-bias-applicability-prediction-model-studies)
2. [PROBAST: A Tool to Assess Risk of Bias and Applicability of Prediction Model Studies: Explanation and Elaboration](https://annals.org/aim/fullarticle/2719962/probast-tool-assess-risk-bias-applicability-prediction-model-studies-explanation)

| **What does PROBAST assess?**  PROBAST assesses both the *risk of bias* and *concerns regarding applicability* of a study that evaluates (develops, validates or updates) a multivariable diagnostic or prognostic prediction model. It is designed to assess primary studies included in a systematic review.  *Bias* occurs if systematic flaws or limitations in the design, conduct or analysis of a primary study distort the results. For the purpose of prediction modelling studies, we have defined *risk of bias* to occur when shortcomings in the study design, conduct or analysis lead to systematically distorted estimates of a model’s predictive performance or to an inadequate model to address the research question. Model predictive performance is typically evaluated using calibration, discrimination and sometimes classification measures, and these are likely inaccurately estimated in studies with high risk of bias. *Applicability* refers to the extent to which the prediction model from the primary study matches your systematic review question, for example in terms of the participants, predictors or outcome of interest.  A primary study may include the development and/or validation or update of more than one prediction model. A PROBAST assessment should be completed for each distinct model that is developed, validated or updated (extended) for making individualised predictions. Where a publication assesses multiple prediction models, only complete a PROBAST assessment for those models that meet the inclusion criteria for your systematic review. Please note that subsequent use of the term “model” includes derivatives of models, such as simplified risk scores, nomograms, or recalibrations of models.  PROBAST is not designed for all multivariable diagnostic or prognostic studies. For example, studies using multivariable models to identify predictors associated with an outcome but not attempting to develop a model for making individualised predictions are not covered by PROBAST.  PROBAST includes four steps.   \| **Step** \| **Task** \| **When to complete** \| \| --- \| --- \| --- \| \| **1** \| Specify your systematic review question(s) \| Once per systematic review \| \| **2** \| Classify the type of prediction model evaluation \| Once for each model of interest in each publication being assessed, for each relevant outcome \| \| **3** \| Assess risk of bias and applicability \| Once for each development and validation of each distinct prediction model in a publication \| \| **4** \| Overall judgment \| Once for each development and validation of each distinct prediction model in a publication \|   If this is your first time using PROBAST, we strongly recommend reading the detailed explanation and elaboration (E&E, see link above) paper and to check the examples on www.probast.org |
| --- | --- | --- | --- | --- | --- | --- | --- | --- | --- | --- | --- | --- | --- | --- | --- |

**Step 1: Specify your systematic review question**

| State your systematic review question to facilitate the assessment of the applicability of the evaluated models to your question. *The following table should be completed once per systematic review.* |
| --- |

| **Criteria** | **Specify your systematic review question** |
| --- | --- |
| *Intended use of model:* | Machine learning tecniques for predicting risk of aggression/violent behaviour |
| ***Participants*** *including selection criteria and setting:* | Psychiatric patients in clinical and forensic settings |
| ***Predictors*** *(used in prediction modelling), including types of predictors (e.g. history, clinical examination, biochemical markers, imaging tests), time of measurement, specific measurement issues (e.g., any requirements/ prohibitions for specialized equipment):* | Clinical, sociodemographic, and historical characteristics.  No specific measurement issues. |
| *Outcome to be predicted:* | Patients with violent behaviours and patients with no violent  behaviour as an outcome |

**Step 2: Classify the type of prediction model evaluation**

| Use the following table to classify the evaluation as model development, model validation or model update, or combination. Different signalling questions apply for different types of prediction model evaluation. If the evaluation does not fit one of these classifications then PROBAST should not be used. |
| --- |

| **Classify the evaluation based on its aim** | | | |
| --- | --- | --- | --- |
| **Type of prediction study** | **PROBAST boxes to complete** | **Tick as appropriate** | **Definition for type of prediction model study** |
| Development only | Development | V | Prediction model development without external validation. These studies may include internal validation methods, such as bootstrapping and cross-validation techniques. |
| Development and validation | Development and validation | X | Prediction model development combined with external validation in other participants in the same article. |
| Validation only | Validation | X | External validation of existing (previously developed) model in other participants. |

| *This table should be completed once for each publication being assessed and for each relevant outcome in your review.* | |  |
| --- | --- | --- |
| **Publication reference** | Watts, D., Moulden, H., Mamak, M., Upfold, C., Chaimowitz, G., & Kapczinski, F. (2021). Predicting offenses among individuals with psychiatric disorders - A machine learning approach. Journal of psychiatric research, 138, 146–154. https://doi.org/10.1016/j.jpsychires.2021.03.026 | |
| **Models of interest** | Random Forest, Elastic Net, SVM | |
| **Outcome of interest** | Aggressive event toward staff or patients | |

**Step 3: Assess risk of bias and applicability**

| PROBAST is structured as four key domains. Each domain is judged for risk of bias (low, high or unclear) and includes signalling questions to help make judgements. Signalling questions are rated as yes (Y), probably yes (PY), probably no (PN), no (N) or no information (NI). All signalling questions are phrased so that “yes” indicates absence of bias. Any signalling question rated as “no” or “probably no” flags the potential for bias; you will need to use your judgement to determine whether the domain should be rated as “high”, “low” or “unclear” risk of bias. The guidance document contains further instructions and examples on rating signalling questions and risk of bias for each domain.  The first three domains are also rated for concerns regarding applicability (low/ high/ unclear) to your review question defined above.  *Complete all domains separately for each evaluation of a distinct model. Shaded boxes indicate where signalling questions do not apply and should not be answered.* |
| --- |

| **DOMAIN 1: Participants** | | | |
| --- | --- | --- | --- |
| **A. Risk of Bias** | | | |
| *Describe the sources of data and criteria for participant selection:*  The present study consisted of 1240 individuals charged with a criminal offense, and subsequently deemed either Unfit to Stand Trial (UST) or Not Criminally Responsible (NCR) as a result of serious mental illness. That comprised a diverse sample of patients from 10 forensic psychiatry facilities, representing patients who were subject to oversight by the Ontario Review Board (ORB) between 2014 and 2015. | | | |
|  | | Dev | Val |
| - 1. Were appropriate data sources used, e.g. cohort, RCT or nested case-control study data? | | PN |  |
| - 1. Were all inclusions and exclusions of participants appropriate? | | NI |  |
| **Risk of bias introduced by selection of participants** | **RISK:**  *(low/ high/ unclear)* | **High** |  |
| *Rationale of bias rating:* | | | |
| Retrospective data. Informed consent was not required. | | | |
| **B. Applicability** | | | |
| *Describe included participants, setting and dates:*  Every participant in the sample was found not criminally responsible or unfit to stand trial for criminal offenses, between 2014 and 2015, 10 forensic psychiatric facilities. | | | |
| **Concern that the included participants and setting do not match the review question** | **CONCERN:**  *(low/ high/ unclear)* | **Low** |  |
| *Rationale of applicability rating:* | | | |
| The study population of the individual paper matches the targeted population of the review question. | | | |

| **DOMAIN 2: Predictors** | | | |
| --- | --- | --- | --- |
| **A. Risk of Bias** | | | |
| *List and describe predictors included in the final model, e.g. definition and timing of assessment:*  The ORB database comprised 246 variables available for analysis, including clinical, demographic, behavioural, and known risk factor variables. Among them, 138 met our threshold of 15% or less missing  data. This involved a diverse set of factors, such as adverse events in childhood, income, housing, comorbidities, family history, prescribed medications, substance use, and presumed indicators of risk. Categorical variables were transformed via one-hot encoding into new binary variables. This resulted in 156 candidate features. | | | |
|  | | Dev | Val |
| - 1. Were predictors defined and assessed in a similar way for all participants? | | Y |  |
| - 1. Were predictor assessments made without knowledge of outcome data? | | PY |  |
| - 1. Are all predictors available at the time the model is intended to be used? | | Y |  |
| **Risk of bias introduced by predictors or their assessment** | **RISK:**  *(low/ high/ unclear)* | **Low** |  |
| *Rationale of bias rating:*  All items of this domain were explicitly or implicitly addressed. | | | |
| **B. Applicability** | | | |
| Concern that the definition, assessment or timing of predictors in the model do not match the review question | **CONCERN:**  *(low/ high/ unclear)* | **Low** |  |
| *Rationale of applicability rating:*  The definition, measurement and timing of the predictors matches the review question and targeted context | | | |

| **DOMAIN 3: Outcome** | | | |
| --- | --- | --- | --- |
| **A. Risk of Bias** | | | |
| *Describe the outcome, how it was defined and determined, and the time interval between predictor assessment and outcome determination:*  Patients were divided into Violent, Nonviolent and Sexual offenses according to the most recent criminal offense for which they were found not criminally responsible. In cases where multiple crimes were committed, patients were divided according to the most serious offense committed. This was operationalized according to the method used within the National Trajectory Project, the largest study in Canada of individuals found not criminally responsible on account of a mental disorder | | | |
|  | | Dev | Val |
| - 1. Was the outcome determined appropriately? | | Y |  |
| - 1. Was a pre-specified or standard outcome definition used? | | Y |  |
| - 1. Were predictors excluded from the outcome definition? | | PY |  |
| - 1. Was the outcome defined and determined in a similar way for all participants? | | Y |  |
| - 1. Was the outcome determined without knowledge of predictor information? | | Y |  |
| - 1. Was the time interval between predictor assessment and outcome determination appropriate? | | PY |  |
| **Risk of bias introduced by the outcome or its determination** | **RISK:**  *(low/ high/ unclear)* | **Low** |  |
| *Rationale of bias rating:*  No major issues. | | | |
| **B. Applicability** | | | |
| *At what time point was the outcome determined:*  Predictors and outcomes are assessed in different time frame.  *If a composite outcome was used, describe the relative frequency/distribution of each contributing outcome:*  N/A | | | |
| **Concern that the outcome, its definition, timing or determination do not match the review question** | **CONCERN:**  *(low/ high/ unclear)* | **Low** |  |
| *Rationale of applicability rating:*  The definition, measurement and timing of the outcome matches the review question and targeted context | | | |

| **DOMAIN 4: Analysis** | | | |
| --- | --- | --- | --- |
| **Risk of Bias** | | | |
| *Describe numbers of participants, number of candidate predictors, outcome events and events per candidate predictor:*  1240 individuals charged with a criminal offense; 138 candidate predictors; 63 patients were charged for violent crimes, 253 patients for non-violent crime, and 124 for sexual crimes, respectively. | | | |
| *Describe how the model was developed (for example in regards to modelling technique (e.g. survival or logistic modelling), predictor selection, and risk group definition):*  In the present study, a data-driven approach to feature selection was used. This encompasses a series of feature selection methods that do not rely on preconceived notions as to which variables will be the most important in the model (Chandrashekar and Sahin, 2014). Specifically, three methods were compared. This included Recursive Feature Elimination (RFE), Ensemble Feature Selection (EFS), and selecting the top 20 weighting factors using variable importance plots. In all methods, feature selection was performed on training data only (70%). | | | |
| *Describe whether and how the model was validated, either internally (e.g. bootstrapping, cross validation, random split sample) or externally (e.g. temporal validation, geographical validation, different setting, different type of participants):*  10-fold cross-validation. | | | |
| *Describe the performance measures of the model, e.g. (re)calibration, discrimination, (re)classification, net benefit, and whether they were adjusted for optimism:*  confusionMatrix, receiver operating characteristic (ROC), sensitivity, specificity, PPV, and NPV. | | | |
| *Describe any participants who were excluded from the analysis:*  *NA* | | | |
| *Describe missing data on predictors and outcomes as well as methods used for missing data:*  Variables with missing data were excluded from the analysis. While some of these excluded variables may have proven to be useful, increasing the imputation threshold from 15% to 30% did not result in a significant change in accuracy in any of the models. | | | |
|  | | Dev | Val |
| - 1. Were there a reasonable number of participants with the outcome? | | PY |  |
| - 1. Were continuous and categorical predictors handled appropriately? | | Y |  |
| - 1. Were all enrolled participants included in the analysis? | | Y |  |
| - 1. Were participants with missing data handled appropriately? | | Y |  |
| - 1. Was selection of predictors based on univariable analysis avoided? | | Y |  |
| - 1. Were complexities in the data (e.g. censoring, competing risks, sampling of controls) accounted for appropriately? | | Y |  |
| - 1. Were relevant model performance measures evaluated appropriately? | | Y |  |
| - 1. Were model overfitting and optimism in model performance accounted for? | | PY |  |
| - 1. Do predictors and their assigned weights in the final model correspond to the results from multivariable analysis? | | NI |  |
| **Risk of bias introduced by the analysis** | **RISK:**  *(low/ high/ unclear)* | **Low** |  |
| *Rationale of bias rating:*  No concerns. | | | |

**Step 4: Overall assessment**

| Use the following tables to reach overall judgements about risk of bias and concerns regarding applicability of the prediction model evaluation (development and/or validation) across all assessed domains.  *Complete for each evaluation of a distinct model.*   \| **Reaching an overall judgement about risk of bias of the prediction model evaluation** \| \| \| --- \| --- \| \| **Low risk of bias** \| If all domains were rated low risk of bias.  If a prediction model was developed without any external validation, and it was rated as low risk of bias for all domains, consider downgrading to **high risk of bias**. Such a model can only be considered as low risk of bias, if the development was based on a very large data set and included some form of internal validation. \| \| **High risk of bias** \| If at least one domain is judged to be at **high risk of bias**. \| \| **Unclear risk of bias** \| If an unclear risk of bias was noted in at least one domain and it was low risk for all other domains. \|  \| **Reaching an overall judgement about applicability of the prediction model evaluation** \| \| \| --- \| --- \| \| **Low concerns regarding applicability** \| If low concerns regarding applicability for all domains, the prediction model evaluation is judged to have **low concerns regarding applicability**. \| \| **High concerns regarding applicability** \| If high concerns regarding applicability for at least one domain, the prediction model evaluation is judged to have **high concerns regarding applicability**. \| \| **Unclear concerns regarding applicability** \| If unclear concerns (but no “high concern”) regarding applicability for at least one domain, the prediction model evaluation is judged to have **unclear concerns regarding applicability** overall. \| |
| --- | --- | --- | --- | --- | --- | --- | --- | --- | --- | --- | --- | --- | --- | --- | --- | --- |

| **Overall judgement about risk of bias and applicability of the prediction model evaluation** | | |
| --- | --- | --- |
| **Overall judgement of risk of bias** | **RISK:**  *(low/ high/ unclear)* | **High** |
| *Summary of sources of potential bias:*  No external validity but it’s based on a very large data set. Data are retrospective. | | |
| **Overall judgement of applicability** | **CONCERN:**  *(low/ high/ unclear)* | **Low** |
| *Summary of applicability concerns:*  No concerns. | | |
